# Supplementary material for: Sex and APOE ε4 genotype modify the Alzheimer’s disease serum metabolome
Source: Nat Commun. 2020 Mar 2;11:1148. doi: 10.1038/s41467-020-14959-w (PMC7052223; doi:10.1038/s41467-020-14959-w)

Supplementary Information for:

Sex and *APOE* ε4 genotype modify the Alzheimer's disease serum metabolome

Matthias Arnold[1,2], Kwangsik Nho[3], Alexandra Kueider-Paisley[1], Tyler Massaro[4], Kevin Huynh[5], Barbara Brauner[2], Siamak MahmoudianDehkordi[1], Gregory Louie[1], M. Arthur Moseley[6], J. Will Thompson[6], Lisa St John Williams[6], Jessica D. Tenenbaum[7], Colette Blach[8], Rui Chang[9], Roberta D. Brinton[9,10,11], Rebecca Baillie[12], Xianlin Han[13], John Q. Trojanowski[14], Leslie M. Shaw[14], Ralph Martins[15,16], Michael W. Weiner[17], Eugenia Trushina[18,19], Jon B. Toledo[14,20], Peter J. Meikle[5], David A. Bennett[4], Jan Krumsiek[22], P. Murali Doraiswamy[1,23,24], Andrew J. Saykin[3], Rima Kaddurah-Daouk[1,23,24], and Gabi Kastenmüller[2,25]

[1] Department of Psychiatry and Behavioral Sciences, Duke University, Durham, NC, USA

[2] Institute of Bioinformatics and Systems Biology, Helmholtz Zentrum München, German Research Center for Environmental Health, Neuherberg, Germany

[3] Department of Radiology and Imaging Sciences and the Indiana Alzheimer Disease Center, Indiana University School of Medicine, Indianapolis, IN, USA

[4] Duke Clinical Research Institute, Duke University, Durham, NC, USA

[5] Metabolomics Laboratory, Baker Heart and Diabetes Institute, Melbourne, Australia

[6] Duke Proteomics and Metabolomics Shared Resource, Center for Genomic and Computational Biology, Duke University, Durham, NC, USA

[7] Department of Biostatistics and Bioinformatics, Duke University, Durham, NC, USA

[8] Duke Molecular Physiology Institute, Duke University, Durham, NC, USA

[9] Center for Innovation in Brain Science, University of Arizona, Tucson, AZ, USA

[10] Department of Pharmacology, College of Medicine, University of Arizona, Tucson, AZ, USA

[11] Department of Neurology, College of Medicine, University of Arizona, Tucson, AZ, USA

[12] Rosa & Co LLC, San Carlos, CA, USA

[13] University of Texas Health Science Center at San Antonio, San Antonio, TX, USA

[14] Department of Pathology & Laboratory Medicine, University of Pennsylvania, Philadelphia, PA, USA

[15] School of Medical and Health Sciences, Edith Cowan University, Joondalup, Australia

[16] Department of Biomedical Sciences, Macquarie University, North Ryde, Australia

[17] Center for Imaging of Neurodegenerative Diseases, Department of Radiology, San Francisco VA Medical Center/University of California San Francisco, San Francisco, CA, USA

[18] Department of Neurology, Mayo Clinic, Rochester, MN, USA

[19] Department of Molecular Pharmacology and Experimental Therapeutics, Mayo Clinic, Rochester, MN, USA

[20] Department of Neurology, Houston Methodist Hospital, Houston, TX, USA

[21] Rush Alzheimer's Disease Center, Rush University Medical Center, Chicago, IL, USA

[22] Institute for Computational Biomedicine, Englander Institute for Precision Medicine, Department of Physiology and Biophysics, Weill Cornell Medicine, New York, NY, USA

[23] Duke Institute of Brain Sciences, Duke University, Durham, NC, USA

[24] Department of Medicine, Duke University, Durham, NC, USA

[25] German Center for Diabetes Research (DZD), Neuherberg, Germany

Contents

Supplementary Notes 3

Supplementary Note 1: Replication Analysis in ROS/MAP 3

Supplementary Note 2: Replication Analysis in AIBL 3

Supplementary Tables 4

Supplementary Table 1: Post-hoc estimated sample sizes required to replicate findings reported in this study 4

Supplementary Figures 6

Supplementary Figure 1: Study rationale and workflow 6

Supplementary Figure 2: Peripheral metabolic sex-differences in the ADNI cohorts 8

Supplementary Figure 3: Estimation and significance of effect heterogeneity exemplified by the association of C2 with CSF p-tau 9

Supplementary Figure 4: Boxplots for all 21 metabolites identified in this study in relation to A-T-N biomarkers in 2-fold stratified analyses 11

Supplementary Figure 5: Quotient normalization for batch removal exemplified for PC ae C44:5 33

Supplementary Figure 6: Distributions of levels of all 139 metabolites included after QC. 34

Supplementary Notes

Supplementary Note 1: Replication Analysis in ROS/MAP

We used metabolomics data obtained from pre-mortem serum samples of deceased participants of the Religious Orders Study and the Rush Memory and Aging Project (ROS/MAP), who had agreed to post-mortem neuropathological examinations, using the same metabolomics kit (AbsoluteIDQ-p180). We had metabolite levels for 596 serum samples of 559 subjects (i.e. we had 37 additional samples from follow-up visits). 89 subjects (126 serum samples) had brain amyloid pathology data (40 CN, 28 MCI, and 21 AD) and 100 subjects (137 serum samples) had brain tau pathology data (46 CN, 28 MCI, and 26 AD), as well as data on all required covariates.

Of the 126 total samples available for brain amyloid pathology analysis, 92 were from females and 34 from males and 97 from *APOE* ε4 non-carriers and 29 from *APOE* ε4 carriers; mean age at visit was 90.24 (± 5.65) years. Of the 137 total samples available for brain tau pathology analysis, 100 were from females and 37 from males and 105 from *APOE* ε4 non-carriers and 32 from *APOE* ε4 carriers; mean age at visit was 90.20 (± 5.74) years.

Metabolomics data processing was performed very similar as for the ADNI, except that we used study sample-based median quotient normalization for batch removal instead of utilizing NIST standard plasma. We then did a targeted analysis to replicate associations of 17 metabolites with amyloid and tau pathology using post-mortem, neuropathology-derived measures of total amyloid load (percent area occupied) and numeric Braak stage as a semi-quantitative measure of severity of neurofibrillary tangle (NFT) pathology in the cortex. Mixed effects regression models were adjusted for fixed effects of age at visit, sex, study cohort (ROS vs. MAP), race, number of copies of *APOE* ε4, as well as years of education and random intercepts for visit and participant identifiers. In stratified analyses, sex and *APOE* ε4, respectively, were omitted as covariates. Heterogeneity estimates were calculated as in ADNI.

Results of association analysis and heterogeneity estimation are provided in Supplementary Table 4.

Supplementary Note 2: Replication Analysis in AIBL

We had access to measurements of CSF p-tau for 94 subjects (82 CN, 7 MCI, and 5 AD) in conjunction with targeted, quantitative lipidomics data (UHPLC ESI-MS/MS platform of the Metabolomics Laboratory of the Baker Heart and Diabetes Institute, Melbourne, Australia). The applied lipidomics technology provides greater resolution than the p180 (which reports many lipids in form of sums of fatty acid chains). With the available data, we were able to derive measures of three metabolites (PC ae C36:1, PC ae C36:2, and SM (OH) C16:1) by summing up the, partly fractionized, levels of the following lipids:

PC ae C36:1

- 100% - PC(O-18:0/18:1)
- 100% - PC(15-MHDA_18:1)
- 100% - PC(17:0_18:1)
- 44% - SM(d16:1/23:0)/SM(d17:1/22:0)

PC ae C36:2

- 100% - PC(O-18:1/18:1)
- 100% - PC(O-18:0/18:2)

- 100% - PC(15-MHDA_18:2)
- 100% - PC(17:0_18:2)

SM (OH) C16:1

- 100% - SM(d16:1/19:0)
- 100% - SM(d18:1/17:0) + SM(d17:1/18:0)

To ascertain that the thus retrieved sums/metabolite measures are comparable, we performed regression analysis of the derived measures against the corresponding p180 metabolites in ADNI-1 for which data on both platforms are available. Overall, R^2-values for these comparisons were >60%, corresponding to an estimated overall correlation of >77.45%, which provides strong evidence for the applicability of this approach.

Of the 94 total subjects available for CSF p-tau analysis, 48 were female and 46 male and 72 *APOE* ε4- and 22 *APOE* ε4+; mean age was 73.9 (± 5.8) years. CSF p-tau data was obtained by analyzing CSF samples in duplicate using the enzyme-linked immunosorbent assay (ELISA): INNOTEST PHOSPHO-TAU(181P) (P-tau181P) (Innogenetics, Ghent, Belgium).

Metabolomics data processing was performed very similar as for the ADNI, except that we used pooled plasma quality control (QC) sample-based median quotient normalization for batch removal instead of utilizing NIST standard plasma. We then did a targeted analysis to replicate associations of the three metabolite measures with CSF p-tau. Association analysis was performed for log-transformed CSF p-tau levels and the three metabolite measures using linear regression while adjusting for sex, age, BMI, *APOE* ε4 status, and level of education (categorized in 5 groups: 0 = 0 to 6 years of education, 1 = 7 to 8 years, 2 = 9 to 12 years, 3 = 13 to 15 years and 4 = 15 years+). In sex-stratified analyses, sex was omitted as covariate. Heterogeneity estimates were calculated as in ADNI.

Results of association analysis and heterogeneity estimation are provided in Supplementary Table 5.

Supplementary Tables

Supplementary Table 1: Post-hoc estimated sample sizes required to replicate findings reported in this study

Supplementary Table 1: Post-hoc estimated sample sizes required to replicate findings reported in this study. Provides for each metabolite reported in the study the sample size required to observe significant (either overall Bonferroni at 9.09 x 10^{-4}, replication at Bonferroni significance for 21 unique metabolites, i.e. 2.38 x 10^{-3}, or nominal significance) replication for a specific association test with the same power as in ADNI. Cells marked in green are those where ADNI was sufficiently powered/detected an effect significant at the respective threshold. Numbers are rough estimates and assume that effect sizes in ADNI can be generalized to the cohort used for replication.

		pooled		males		females		sex difference	*APOE* ε4+		*APOE* ε4-		*APOE* ε4 status difference
biomarker	metabolite	bonferroni[a]	replication[b]	bonferroni[a]	replication[b]	bonferroni[a]	replication[b]	replication[c]	bonferroni[a]	replication[b]	bonferroni[a]	replication[b]	replication[c]
	PC ae C44:6	898	753	869	729	885	742	364,160	316	265	2,089	1,753	570
	PC ae C44:4	975	818	880	738	1,158	972	293,270	382	320	2,556	2,144	679
	PC ae C44:5	995	835	760	637	1,572	1,319	15,236	318	266	2,904	2,436	519
Pathological CSF $A\beta_{1-42}$	Threonine	1,627	1,364	596	500	12,828	10,760	1,103	891	747	2,726	2,287	2,727
	Valine	4,545	3,812	83,468	70,010	915	768	1,070	2,695	2,260	3,242	2,720	35,004
	PC ae C42:4	1,251	1,049	1,001	840	1,672	1,402	43,946	382	321	4,175	3,502	622
	Proline	13,733	11,519	25,082,732	21,038,555	2,818	2,364	4,276	3,591	3,013	1,585	1,330	883
	Glycine	21,779	18,268	1,879	1,576	10,015	8,400	1,144	995	834	6,081	5,101	647
	C10	1,481	1,242	56,630	47,499	458	384	859	1,178	988	2,325	1,950	16,528
	C5−DC (C6−OH)	2,318	1,945	186,346	156,301	577	484	931	3,792	3,181	2,462	2,065	98,579
	C8	2,655	2,227	1,104,739	926,618	686	576	1,013	2,889	2,423	3,602	3,022	1,225,198
	PC ae C36:2	4,051	3,398	820	688	21,631	18,144	752	527,426	442,388	1,238	1,038	1,901
CSF p-tau	Histidine	9,880	8,287	10,898	9,141	989	830	833	6,163	5,169	11,961	10,033	93,885
	Asparagine	10,389	8,714	1,137	954	3,769	3,161	641	3,881,215	3,255,433	6,503	5,455	10,408
	SM (OH) C16:1	11,545	9,684	1,445	1,212	7,569	6,349	976	39,206	32,885	1,935	1,623	1,848
	Glycine	13,613	11,419	1,537	1,289	13,207	11,078	1,027	2,404	2,016	1,178,118	988,166	3,089
	PC ae C36:1	14,687	12,319	1,572	1,319	6,184	5,187	950	43,152	36,195	3,580	3,003	3,045
	C2	39,982	33,536	3,413	2,863	1,144	960	653	959,881	805,116	28,450	23,863	53,765
	PC aa C32:1	702	589	598	502	944	792	17,667	1,557	1,306	464	389	3,003
	PC ae C44:4	926	776	1,248	1,047	576	483	8,558	960	805	908	762	8,212,123
	PC ae C44:5	1,006	844	918	770	906	760	10,245,998	835	700	1,097	920	41,260
	PC aa C32:0	1,091	915	913	766	1,358	1,139	14,673	716	600	1,998	1,676	6,570
	PC ae C42:4	1,132	949	1,198	1,005	912	765	180,418	763	640	1,685	1,413	8,930
FDG-PET	C10	3,313	2,779	1,997	1,675	10,229	8,580	7,817	8,828	7,405	591	496	547
	C8	4,636	3,888	2,108	1,768	126,689	106,263	4,063	8,324	6,982	678	569	565
	Valine	9,478	7,950	31,829	26,697	4,219	3,539	17,708	8,473	7,107	1,184	993	882
	Glycine	11,699	9,813	4,927	4,133	26,807	22,485	10,491	662	555	3,756	3,151	462
	Proline	22,157	18,585	3,823	3,206	799	670	546	1,290	1,082	5,429	4,554	815
	C16:1	1,143	959	460	386	14,711	12,339	927	1,846	1,548	865	725	14,166
	PC ae C40:2	4,055	3,401	859	720	41,943	35,180	879	1,886	1,582	10,363	8,693	9,057

[a] **at $P \leq 9.09 \times 10^{-4}$**
[b] **at $P \leq 2.38 \times 10^{-3}$**
[c] **at $P \leq 0.05$**

Supplementary Figures

Supplementary Figure 1: Study rationale and workflow

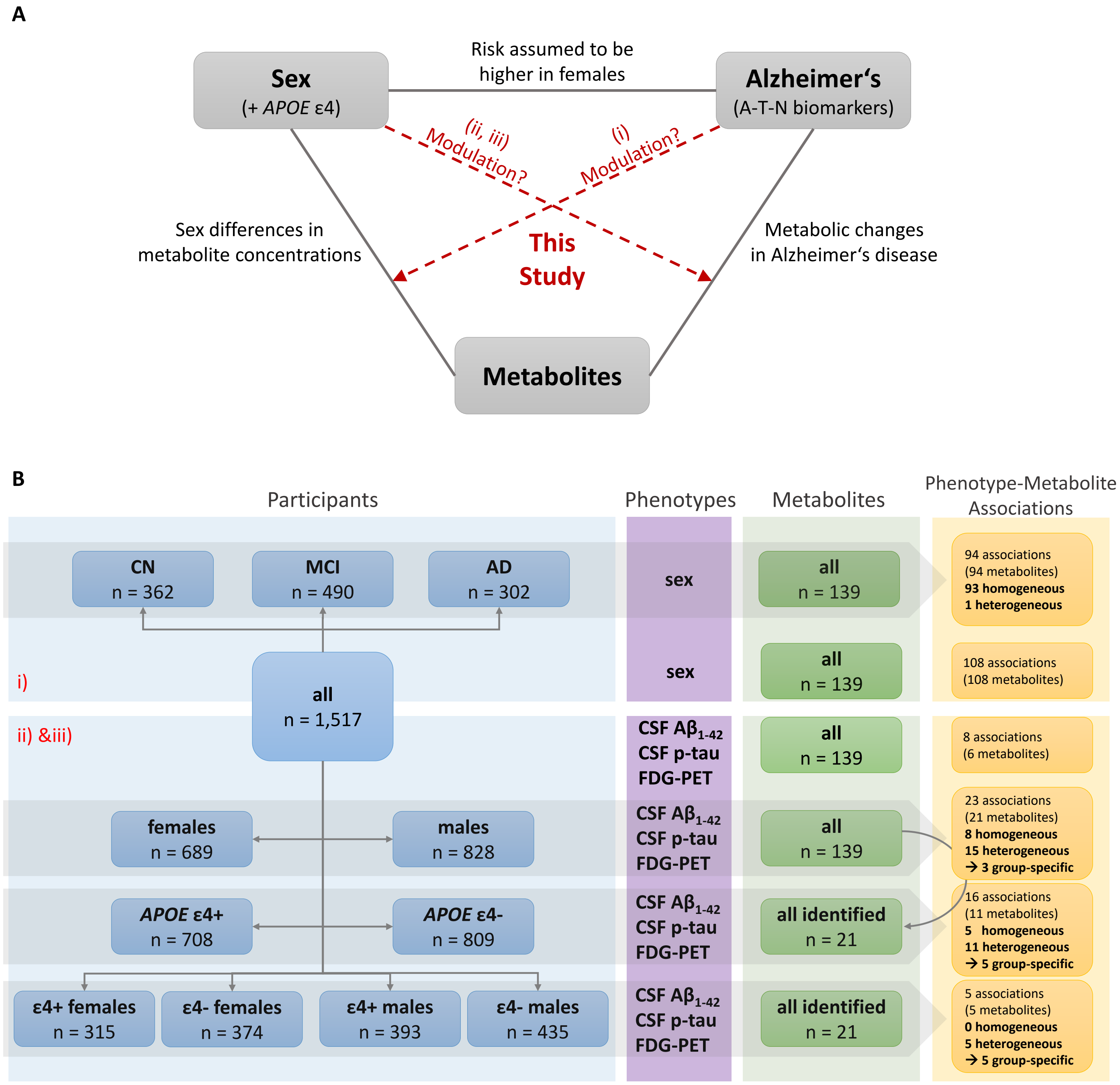

Supplementary Figure 1: Study rationale and workflow. A) This study aims to investigate the relationship between AD, sex, and metabolic readouts in a systematic fashion. The background of this work is: firstly, it has been reported that AD risk may be increased in females; secondly, there are strongly pronounced, highly significant, and often replicated sex-differences in blood metabolite concentrations in the general, healthy population; and, thirdly, we and others have shown that there are significant associations of metabolite levels with AD and its biomarkers. In the current study, we examined: (i) if clinical diagnosis of MCI or AD influences peripheral metabolic sex-differences as seen in healthy controls, (ii) if sex modulates associations of metabolite levels with three AD biomarkers across the A-T-N spectrum, and, (iii), if effects of metabolites showing sex-based effect heterogeneity in their associations with AD are also modulated by *APOE* ε4 status. **B)** To address the three research questions of this study, we first performed analyses of sex-metabolite associations for 139 metabolites in the ADNI cohort stratified by diagnostic group (question i). Subsequently, we performed phenotype (A-T-N)-metabolite associations for 139 metabolites in the ADNI cohort stratified by sex (question ii) and stratified by *APOE* ε4 status; additionally, we performed phenotype (A-T-N)-metabolite associations for the 21 significantly associated metabolites after stratification by sex plus *APOE* ε4 status (question iii).

Supplementary Figure 2: Peripheral metabolic sex-differences in the ADNI cohorts

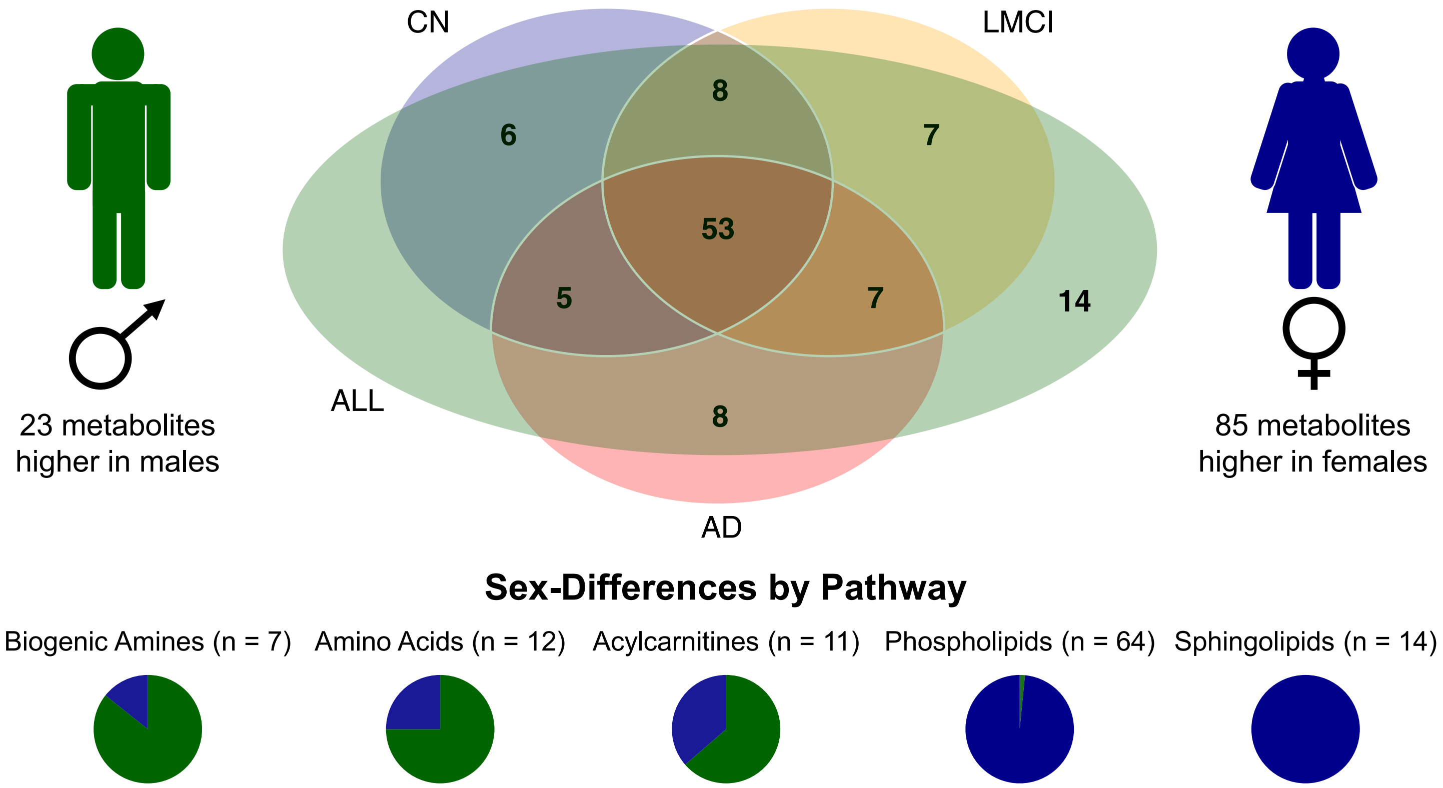

Supplementary Figure 2: Peripheral metabolic sex-differences in the ADNI cohorts. We tested whether sex-associated differences in blood metabolite levels differ between patients with probable AD, subjects with MCI, and CN subjects in the ADNI cohorts. We found 108 of 139 metabolites to be significantly associated with sex after multiple testing correction while adjusting for age, BMI, ADNI study phase, and diagnostic group. 70 of these associations replicate previous findings in a healthy population-based cohort. All SMs and the majority of PCs were more abundant in women. The majority of biogenic amines, amino acids, and acylcarnitines were more abundant in men. Stratifying subjects by diagnostic group revealed that 53 of the 108 metabolites showing significant sex-differences were also significant in each of the three groups (AD, MCI, CN) alone, while 14 metabolites showed no significant difference in any of the groups, probably due to lower statistical power after stratification. No significant sex-differences were found that were not also significant in the unstratified analysis.

Supplementary Figure 3: Estimation and significance of effect heterogeneity exemplified by the association of C2 with CSF p-tau

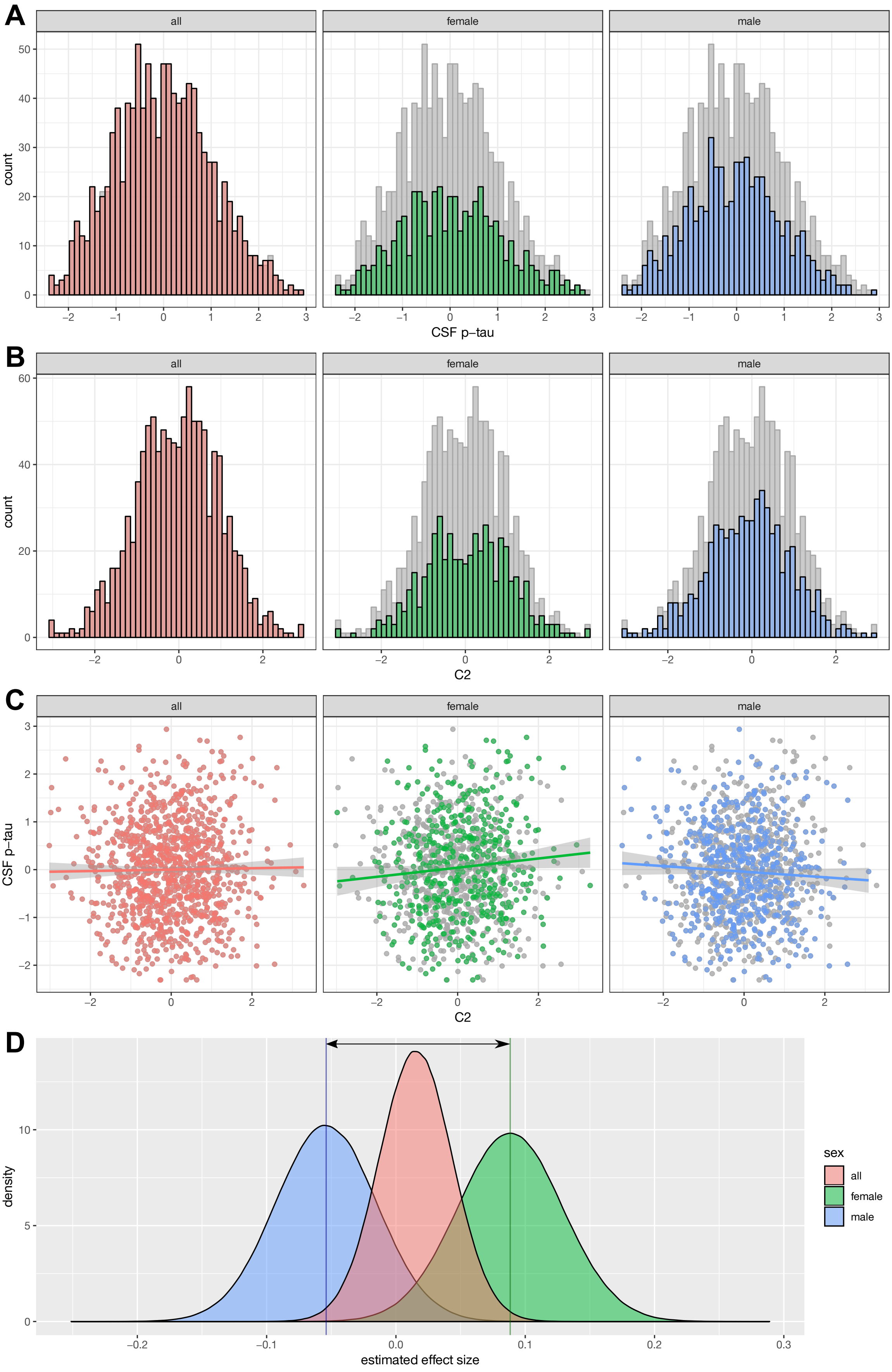

Supplementary Figure 3: Estimation and significance of effect heterogeneity exemplified by the association of C2 with CSF p-tau. A: Histograms of the distribution of log2-transformed CSF p-tau levels in all samples, and for females and males separately. **B:** Histograms of the distribution of log2-transformed, Z-scored C2 levels in all samples, and for females and males separately. **C:** Scatter plots of C2 levels versus CSF p-tau levels plus regression lines. In all samples, there is absolutely no effect, in females there is a nominally significant correlation, and in males, there is a non-significant opposite trend (i.e. negative correlation). **D:** Density plots of the estimated effects in all samples, females, and males. Heterogeneity estimation as applied here investigates if there is a significant difference in the estimated distributions of association effects seen for two strata (here, females and males). These analyses are not directly aiming at determining the direction of effects in one of the strata (which may change for insignificant associations), but are examining if there is a significant difference in the variance-weighted estimated effects. A significant test thus identifies associations that show significantly different effects in two strata that cannot be identified in a pooled analysis adjusting for the stratifying variable, as seen in the density curve of the estimated effect distribution across all samples (which is centered close to 0). These analyses are naturally assuming a close-to-normal distribution of effect sizes (as is regression), which we could validate for >90% of investigated effects in the bootstrap analyses (Supplementary Analysis 1).

Supplementary Figure 4: Boxplots for all 21 metabolites identified in this study in relation to A-T-N biomarkers in 2-fold stratified analyses

Boxplots for all 21 metabolites identified in this study to show their relation to A-T-N biomarkers (A: pathological CSF A$\beta_{1\text{-}42}$; T: mean-split CSF p-tau levels; N: mean-split FDG-PET values) for 2-fold stratified analyses by both sex and *APOE* ε4 status. *APOE* ε4 status groups are plotted in separate panels, females and males are distinguished by color (f: blue, m: green), and binarized biomarker groups are emphasized by lighter (lower-risk biomarker profile) and deeper (higher-risk biomarker profile) colors.

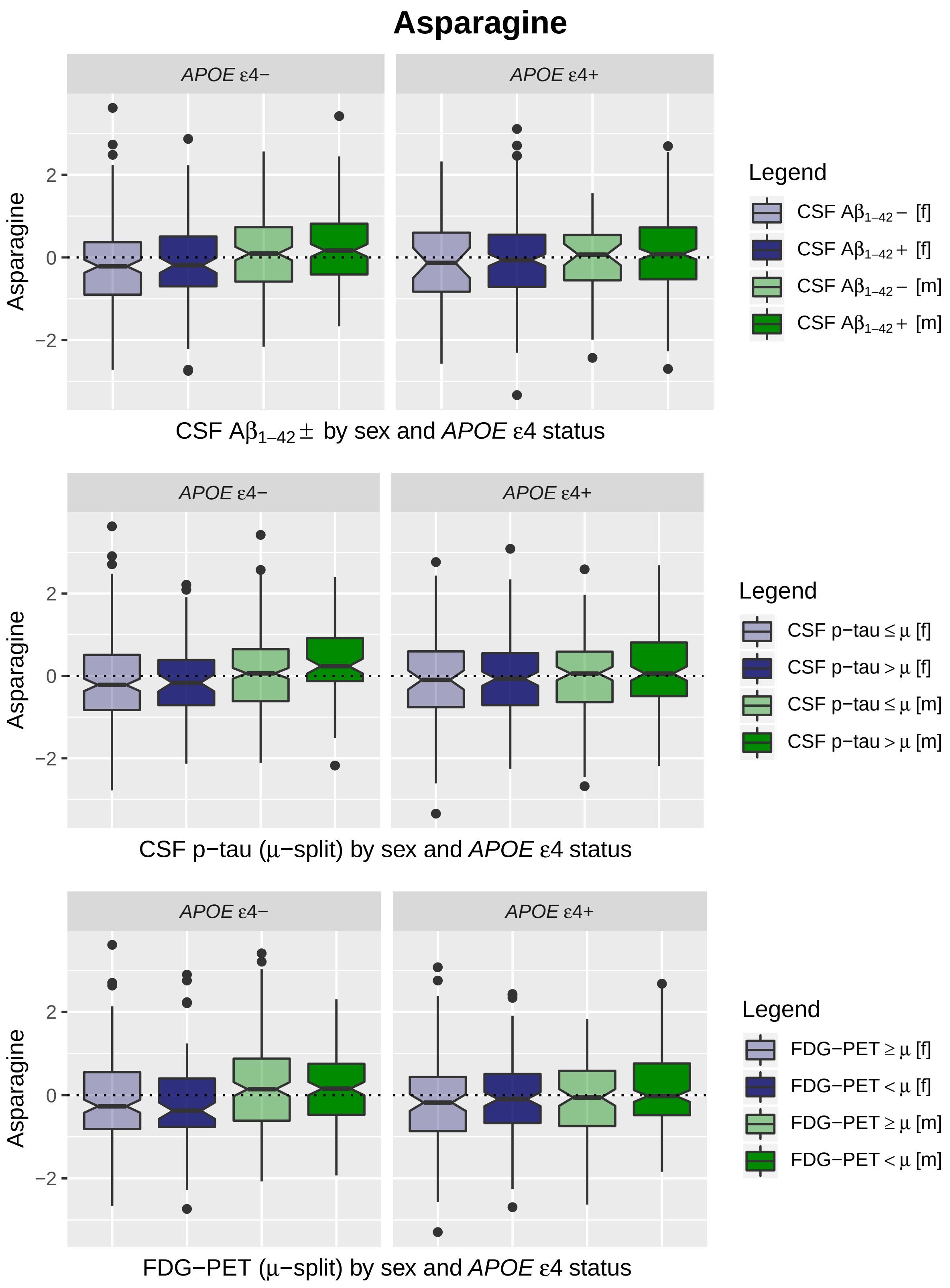

Asparagine
APOE ε4−
APOE ε4+
Asparagine
2
0
−2
Legend
CSF Aβ1–42 − [f]
CSF Aβ1–42 + [f]
CSF Aβ1–42 − [m]
CSF Aβ1–42 + [m]
CSF Aβ1–42 ± by sex and APOE ε4 status
APOE ε4−
APOE ε4+
Asparagine
2
0
−2
Legend
CSF p−tau ≤ μ [f]
CSF p−tau > μ [f]
CSF p−tau ≤ μ [m]
CSF p−tau > μ [m]
CSF p−tau (μ−split) by sex and APOE ε4 status
APOE ε4−
APOE ε4+
Asparagine
2
0
−2
Legend
FDG−PET ≥ μ [f]
FDG−PET < μ [f]
FDG−PET ≥ μ [m]
FDG−PET < μ [m]
FDG−PET (μ−split) by sex and APOE ε4 status

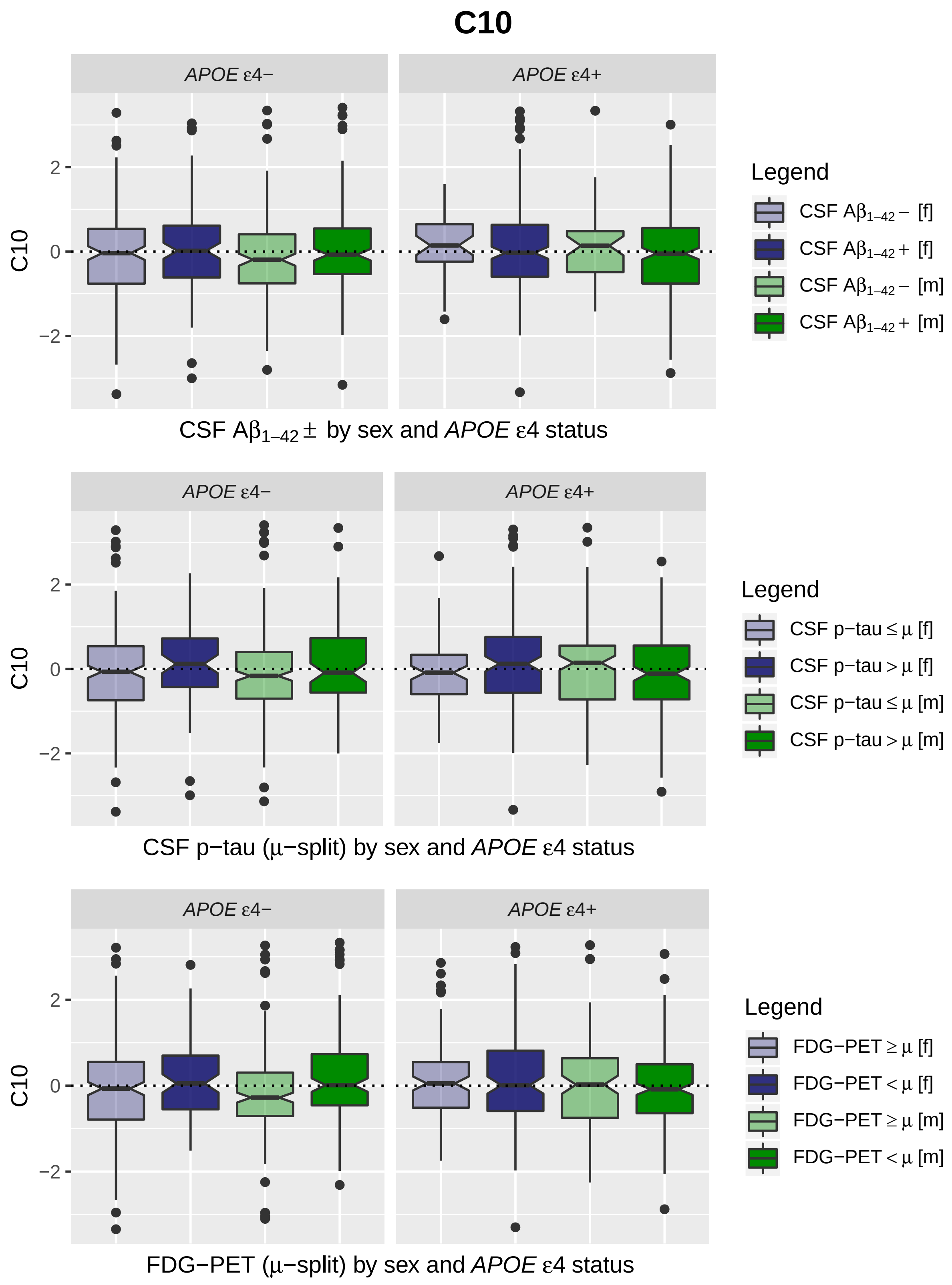

C10
APOE ε4−
APOE ε4+
2
0
−2
C10
CSF Aβ1−42 ± by sex and APOE ε4 status
Legend
CSF Aβ1−42 − [f]
CSF Aβ1−42 + [f]
CSF Aβ1−42 − [m]
CSF Aβ1−42 + [m]
CSF p−tau (μ−split) by sex and APOE ε4 status
CSF p−tau ≤ μ [f]
CSF p−tau > μ [f]
CSF p−tau ≤ μ [m]
CSF p−tau > μ [m]
FDG−PET (μ−split) by sex and APOE ε4 status
FDG−PET ≥ μ [f]
FDG−PET < μ [f]
FDG−PET ≥ μ [m]
FDG−PET < μ [m]

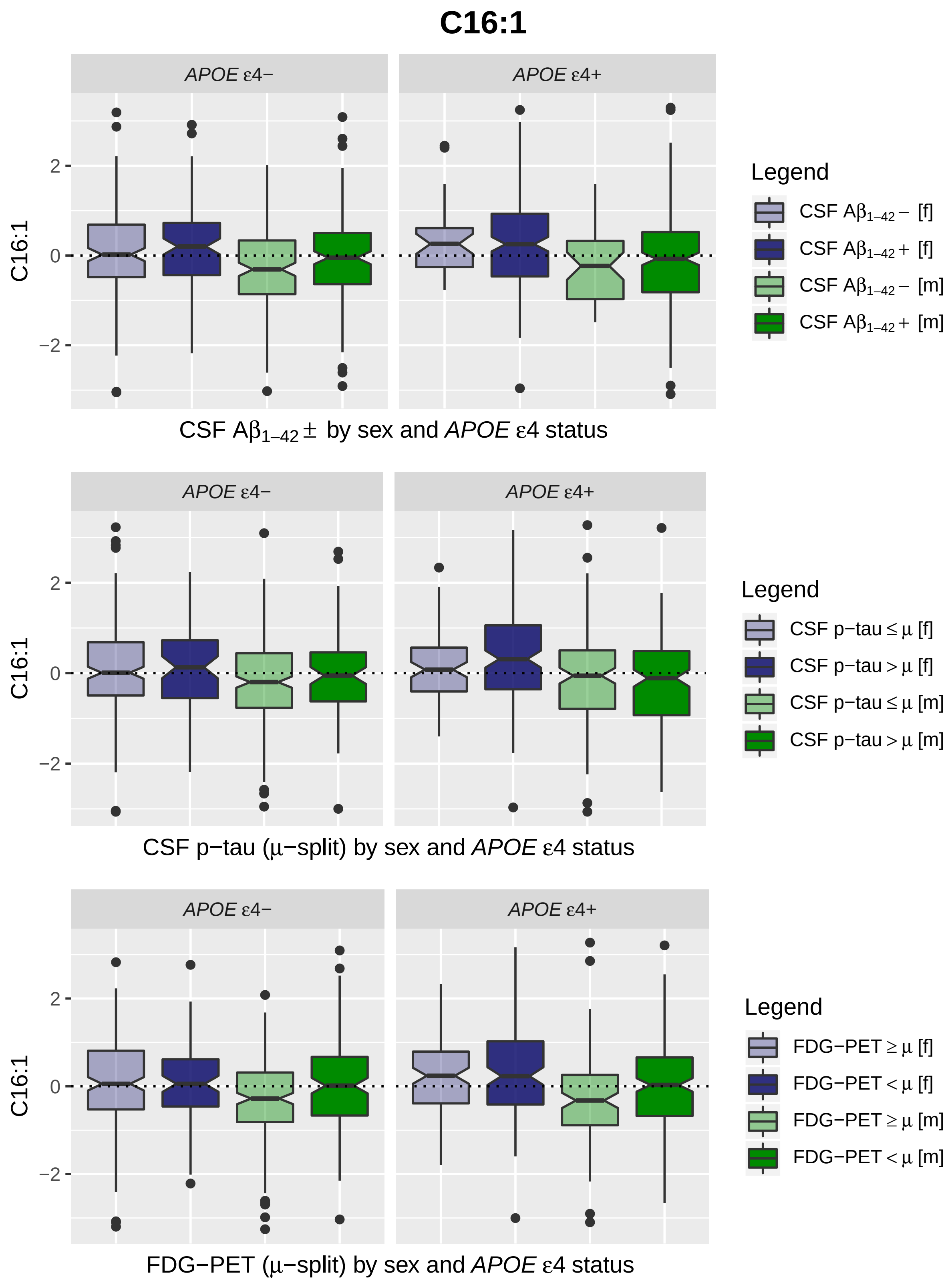

C16:1
APOE ε4−
APOE ε4+
C16:1
2
0
−2
Legend
CSF Aβ1–42 − [f]
CSF Aβ1–42 + [f]
CSF Aβ1–42 − [m]
CSF Aβ1–42 + [m]
CSF Aβ1–42 ± by sex and APOE ε4 status
Legend
CSF p−tau ≤ μ [f]
CSF p−tau > μ [f]
CSF p−tau ≤ μ [m]
CSF p−tau > μ [m]
CSF p−tau (μ−split) by sex and APOE ε4 status
Legend
FDG−PET ≥ μ [f]
FDG−PET < μ [f]
FDG−PET ≥ μ [m]
FDG−PET < μ [m]
FDG−PET (μ−split) by sex and APOE ε4 status

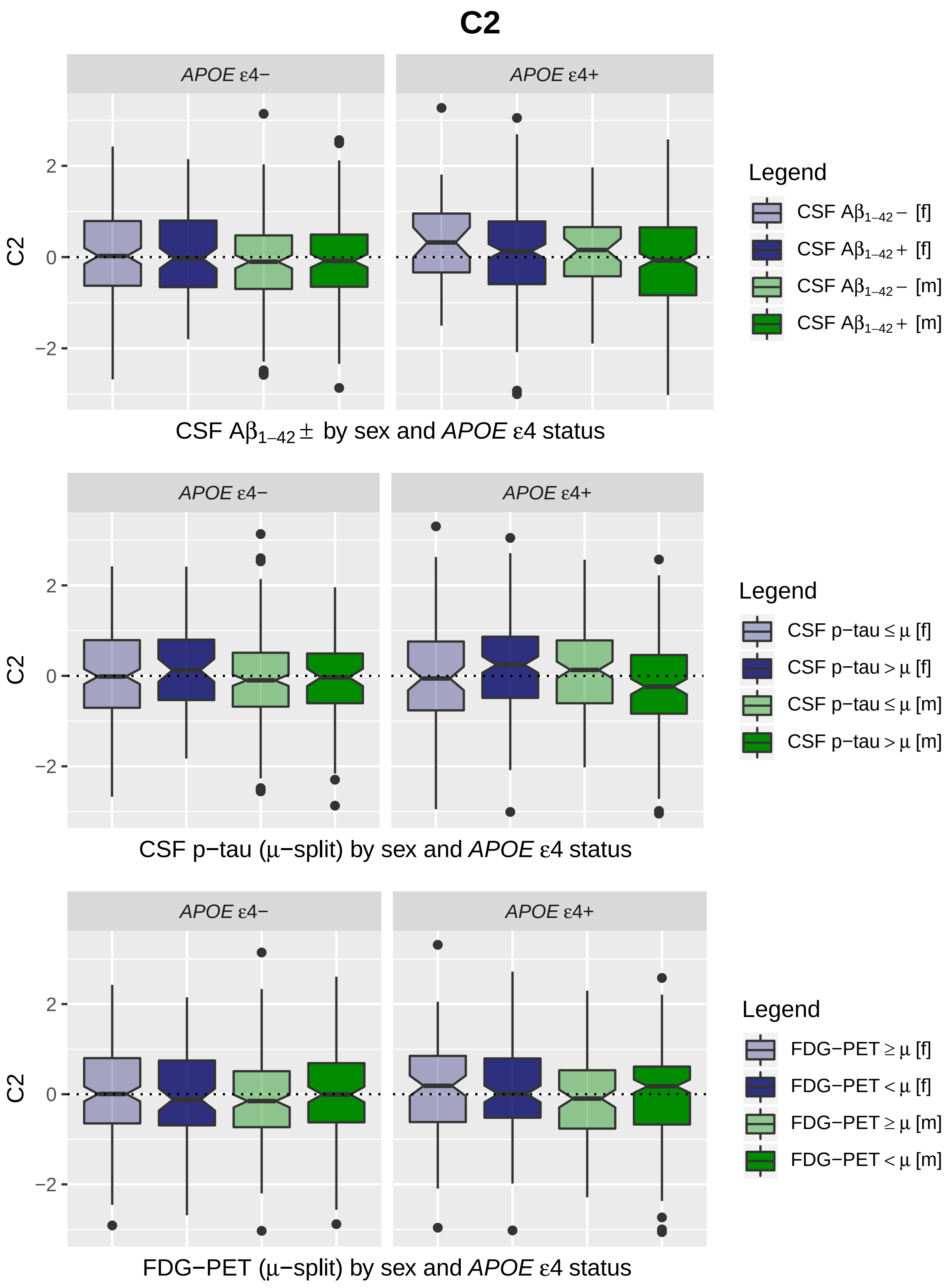
C2
APOE ε4−
APOE ε4+
C2
2
0
−2
Legend
CSF Aβ1–42 − [f]
CSF Aβ1–42 + [f]
CSF Aβ1–42 − [m]
CSF Aβ1–42 + [m]
CSF Aβ1–42 ± by sex and APOE ε4 status
APOE ε4−
APOE ε4+
C2
2
0
−2
Legend
CSF p−tau ≤ μ [f]
CSF p−tau > μ [f]
CSF p−tau ≤ μ [m]
CSF p−tau > μ [m]
CSF p−tau (μ−split) by sex and APOE ε4 status
APOE ε4−
APOE ε4+
C2
2
0
−2
Legend
FDG−PET ≥ μ [f]
FDG−PET < μ [f]
FDG−PET ≥ μ [m]
FDG−PET < μ [m]
FDG−PET (μ−split) by sex and APOE ε4 status

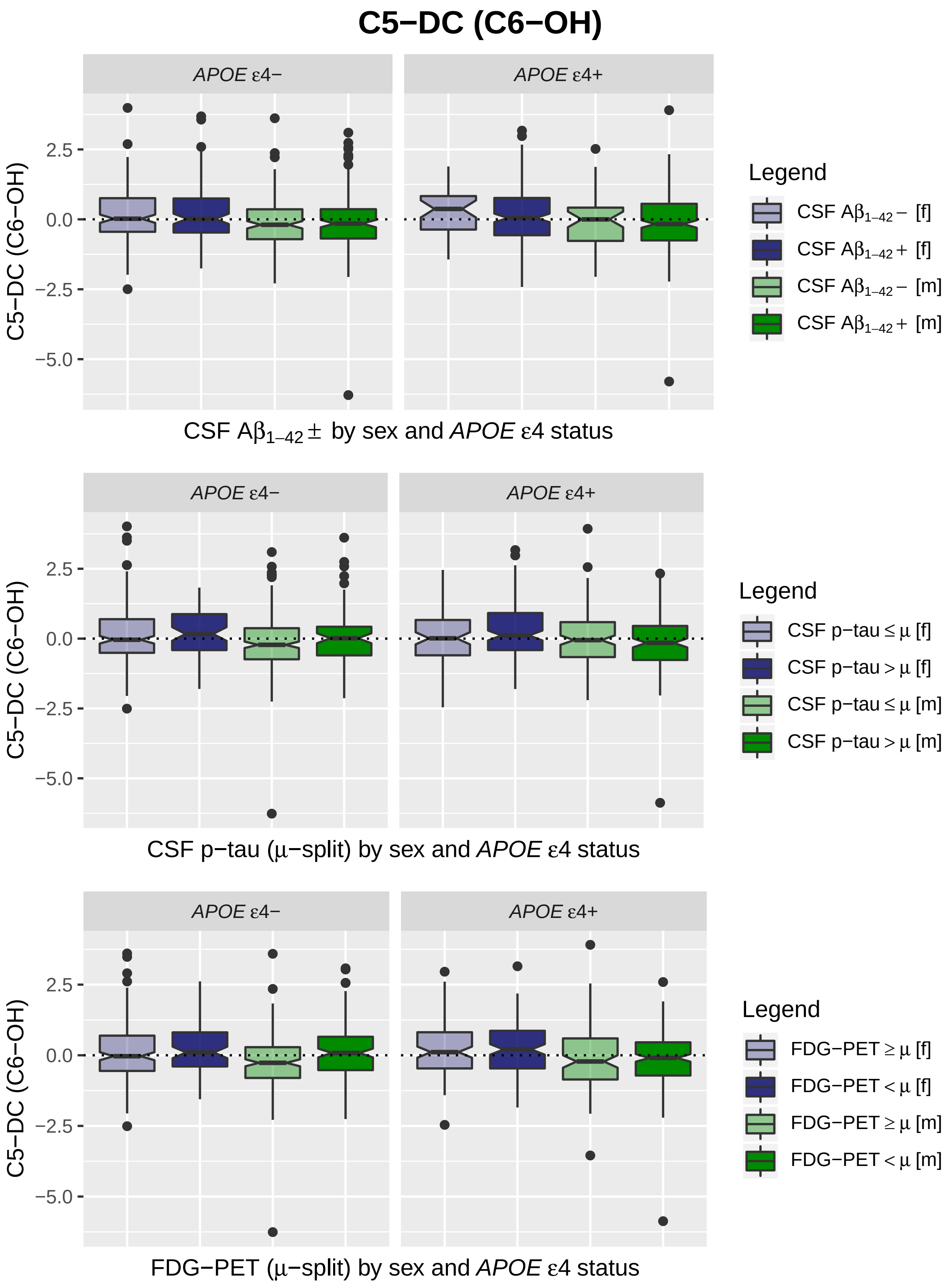

C5−DC (C6−OH)
APOE ε4−
APOE ε4+
C5−DC (C6−OH)
2.5
0.0
−2.5
−5.0
CSF Aβ1−42 ± by sex and APOE ε4 status
Legend
CSF Aβ1−42 − [f]
CSF Aβ1−42 + [f]
CSF Aβ1−42 − [m]
CSF Aβ1−42 + [m]
CSF p−tau (μ−split) by sex and APOE ε4 status
CSF p−tau ≤ μ [f]
CSF p−tau > μ [f]
CSF p−tau ≤ μ [m]
CSF p−tau > μ [m]
FDG−PET (μ−split) by sex and APOE ε4 status
FDG−PET ≥ μ [f]
FDG−PET < μ [f]
FDG−PET ≥ μ [m]
FDG−PET < μ [m]

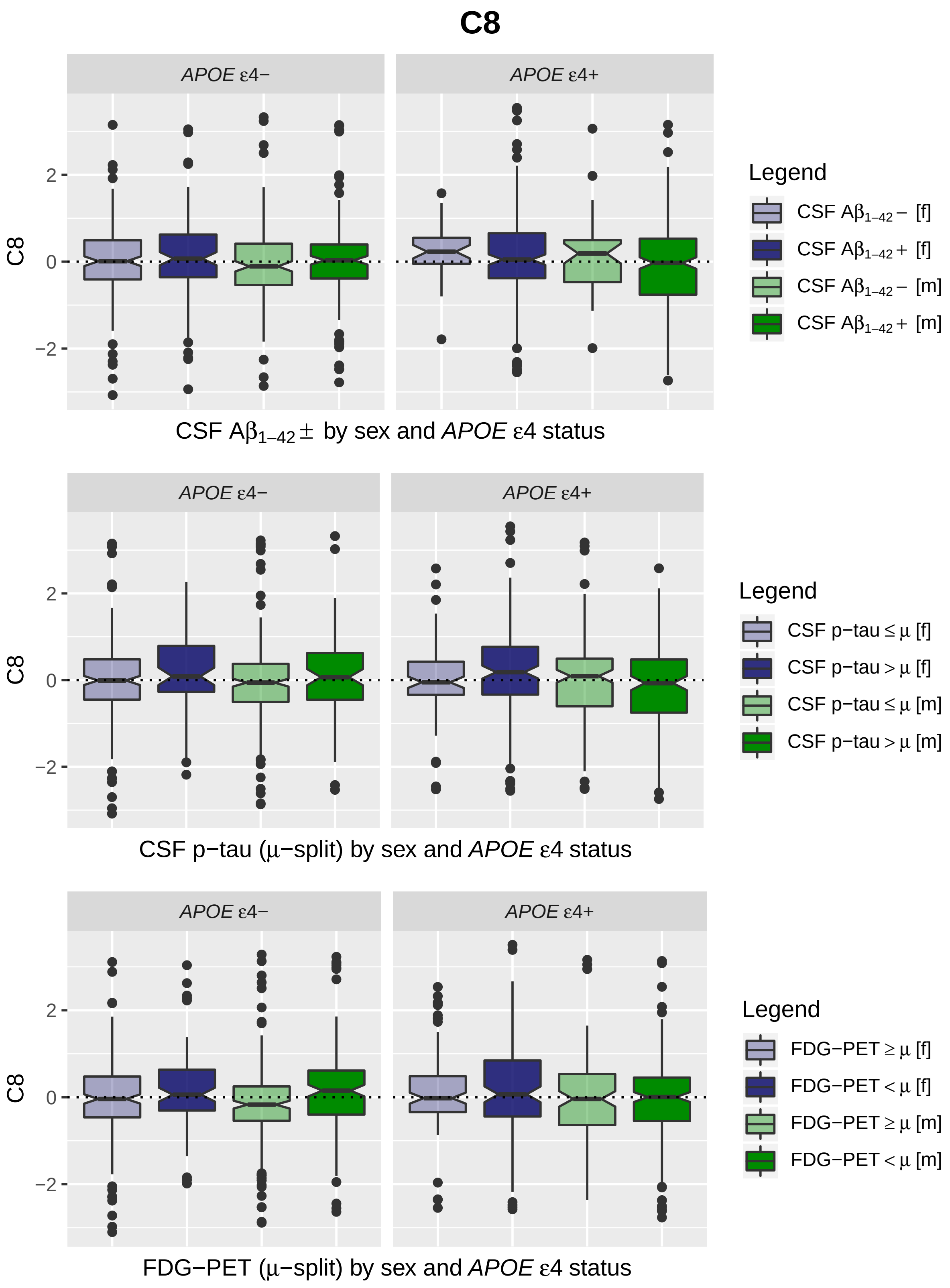
C8
APOE ε4−
APOE ε4+
C8
2
0
−2
CSF Aβ1−42 ± by sex and APOE ε4 status
Legend
CSF Aβ1−42 − [f]
CSF Aβ1−42 + [f]
CSF Aβ1−42 − [m]
CSF Aβ1−42 + [m]
CSF p−tau (μ−split) by sex and APOE ε4 status
Legend
CSF p−tau ≤ μ [f]
CSF p−tau > μ [f]
CSF p−tau ≤ μ [m]
CSF p−tau > μ [m]
FDG−PET (μ−split) by sex and APOE ε4 status
Legend
FDG−PET ≥ μ [f]
FDG−PET < μ [f]
FDG−PET ≥ μ [m]
FDG−PET < μ [m]

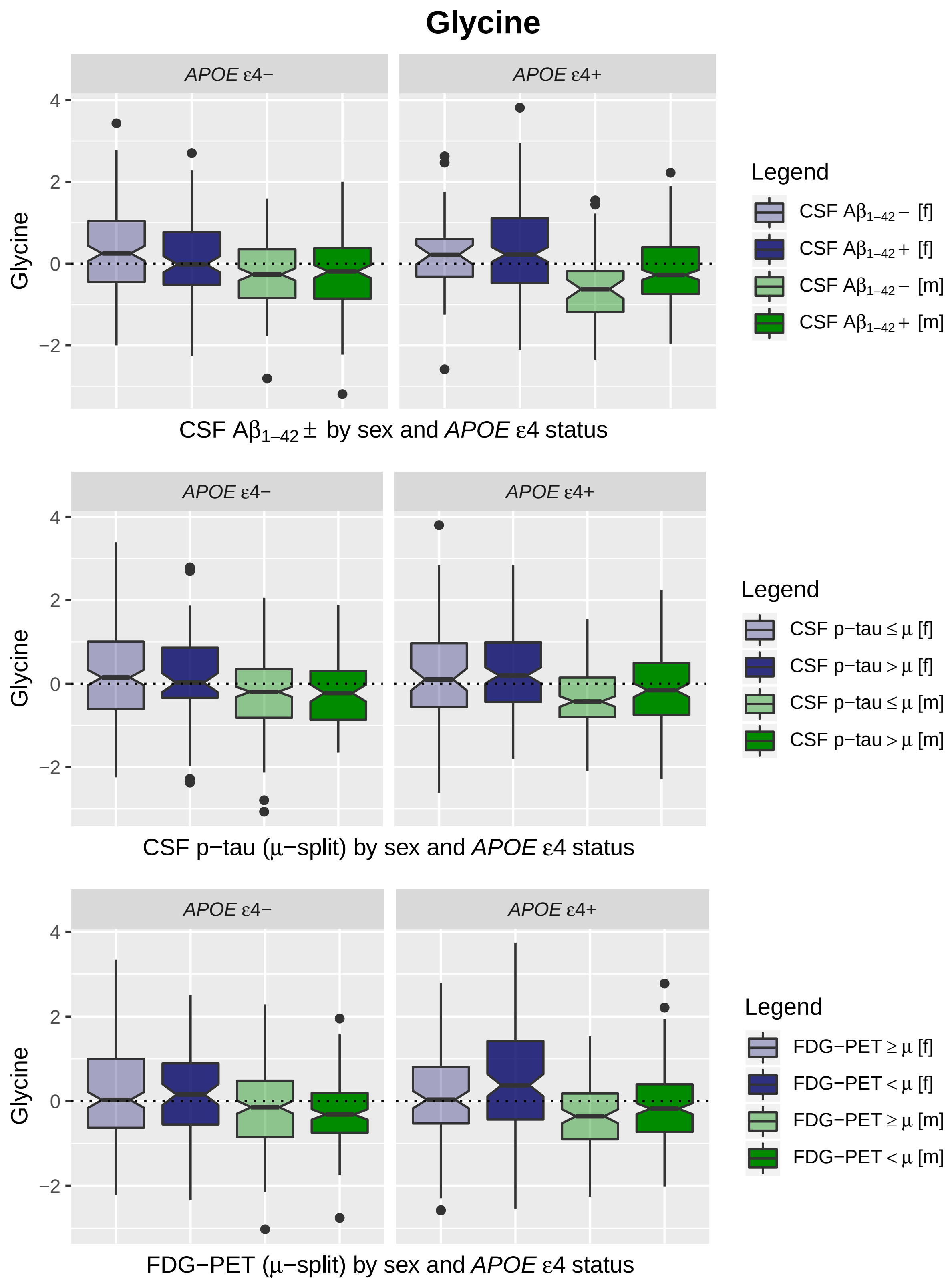
Glycine
APOE ε4−
APOE ε4+
4
2
0
−2
Glycine
Legend
CSF Aβ1–42 − [f]
CSF Aβ1–42 + [f]
CSF Aβ1–42 − [m]
CSF Aβ1–42 + [m]
CSF Aβ1–42 ± by sex and APOE ε4 status
CSF p−tau ≤ μ [f]
CSF p−tau > μ [f]
CSF p−tau ≤ μ [m]
CSF p−tau > μ [m]
CSF p−tau (μ−split) by sex and APOE ε4 status
FDG−PET ≥ μ [f]
FDG−PET < μ [f]
FDG−PET ≥ μ [m]
FDG−PET < μ [m]
FDG−PET (μ−split) by sex and APOE ε4 status

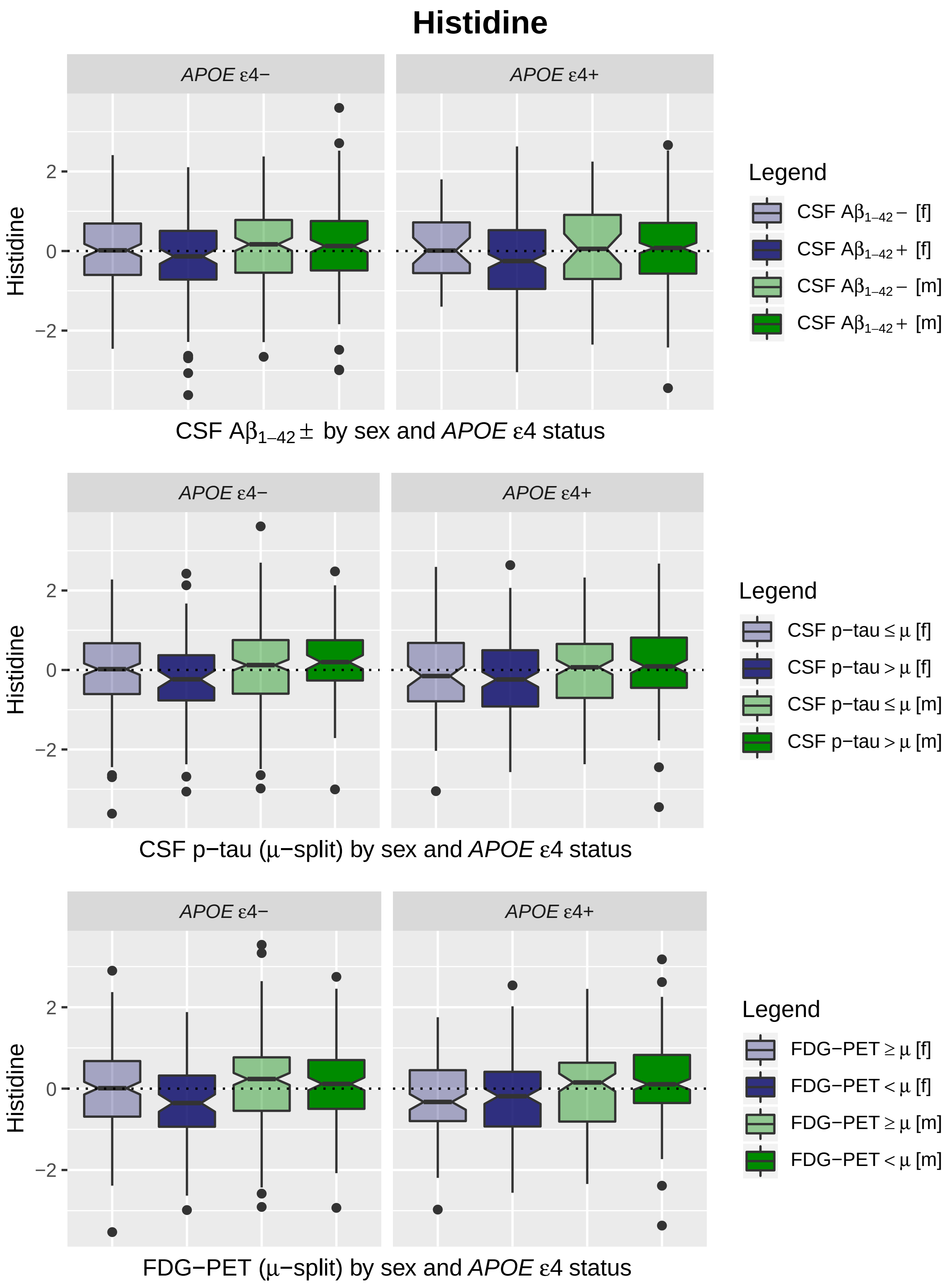
Histidine
APOE ε4−
APOE ε4+
Histidine
2
0
−2
Legend
CSF Aβ1–42 − [f]
CSF Aβ1–42 + [f]
CSF Aβ1–42 − [m]
CSF Aβ1–42 + [m]
CSF Aβ1–42 ± by sex and APOE ε4 status
Legend
CSF p−tau ≤ μ [f]
CSF p−tau > μ [f]
CSF p−tau ≤ μ [m]
CSF p−tau > μ [m]
CSF p−tau (μ−split) by sex and APOE ε4 status
Legend
FDG−PET ≥ μ [f]
FDG−PET < μ [f]
FDG−PET ≥ μ [m]
FDG−PET < μ [m]
FDG−PET (μ−split) by sex and APOE ε4 status

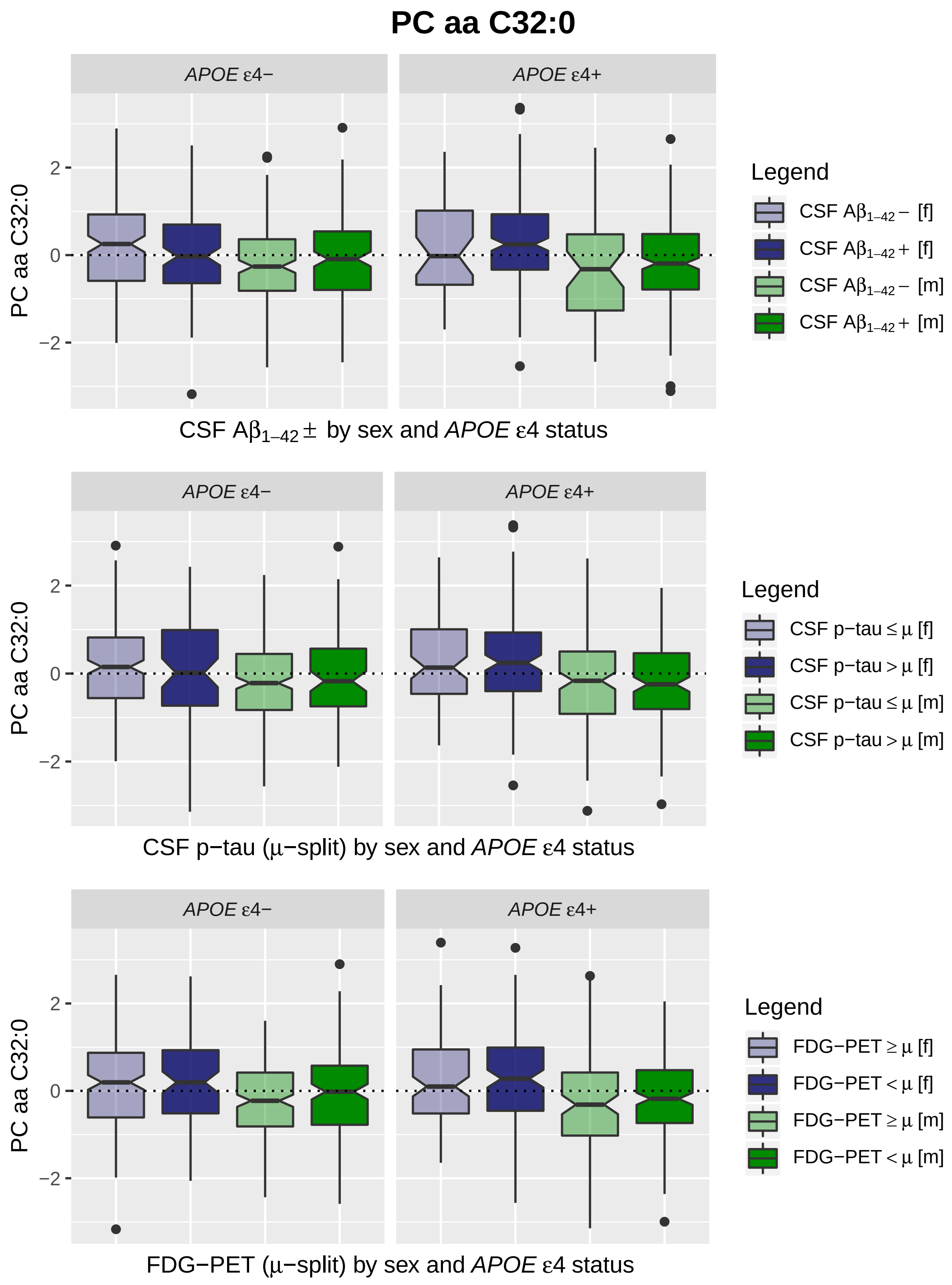
PC aa C32:0
APOE ε4−
APOE ε4+
PC aa C32:0
2
0
−2
Legend
CSF Aβ1–42 − [f]
CSF Aβ1–42 + [f]
CSF Aβ1–42 − [m]
CSF Aβ1–42 + [m]
CSF Aβ1–42 ± by sex and APOE ε4 status
CSF p−tau ≤ μ [f]
CSF p−tau > μ [f]
CSF p−tau ≤ μ [m]
CSF p−tau > μ [m]
CSF p−tau (μ−split) by sex and APOE ε4 status
FDG−PET ≥ μ [f]
FDG−PET < μ [f]
FDG−PET ≥ μ [m]
FDG−PET < μ [m]
FDG−PET (μ−split) by sex and APOE ε4 status

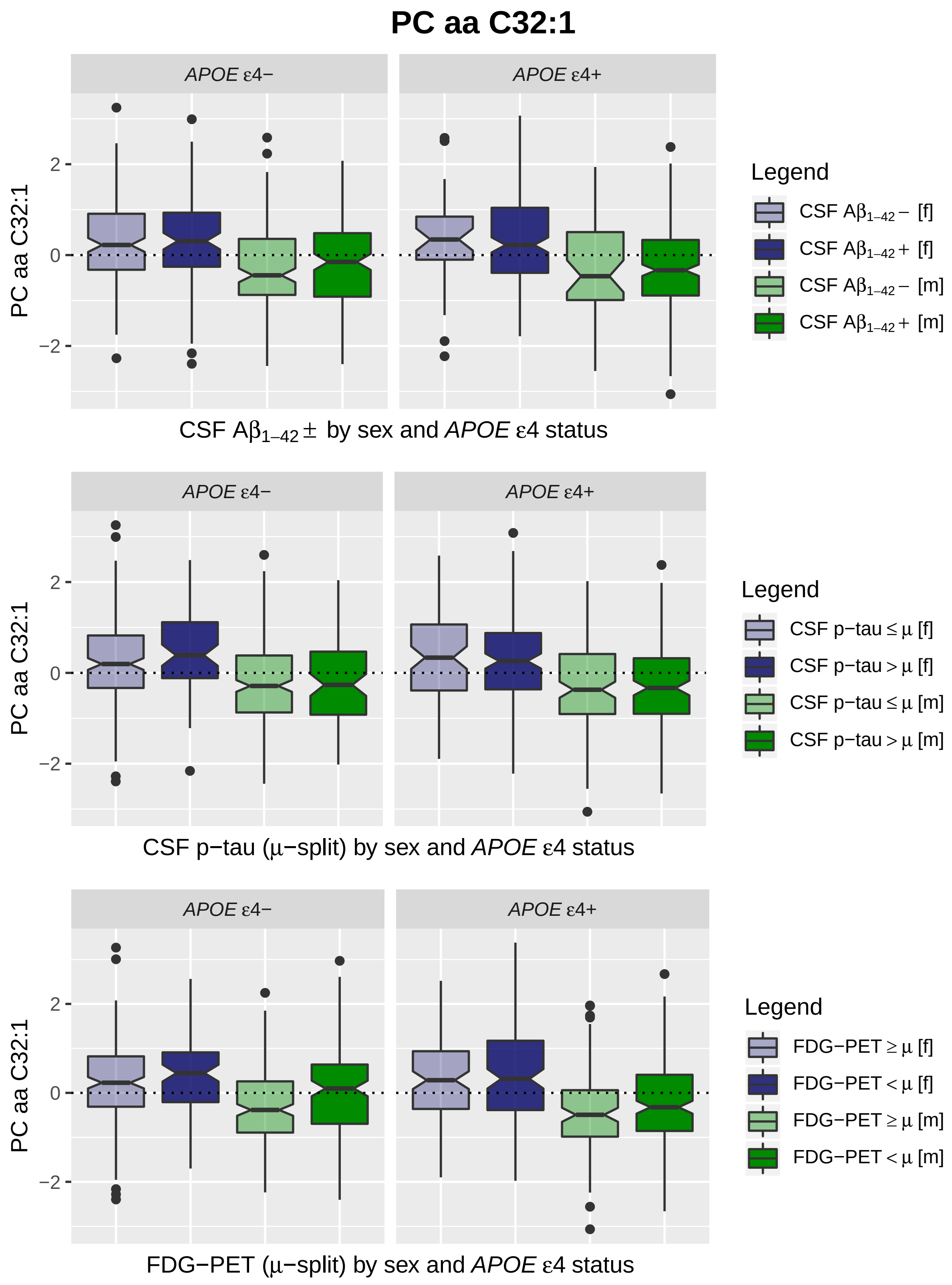

PC aa C32:1
APOE ε4−
APOE ε4+
PC aa C32:1
2
0
−2
Legend
CSF Aβ1−42 − [f]
CSF Aβ1−42 + [f]
CSF Aβ1−42 − [m]
CSF Aβ1−42 + [m]
CSF Aβ1−42 ± by sex and APOE ε4 status
CSF p−tau ≤ μ [f]
CSF p−tau > μ [f]
CSF p−tau ≤ μ [m]
CSF p−tau > μ [m]
CSF p−tau (μ−split) by sex and APOE ε4 status
FDG−PET ≥ μ [f]
FDG−PET < μ [f]
FDG−PET ≥ μ [m]
FDG−PET < μ [m]
FDG−PET (μ−split) by sex and APOE ε4 status

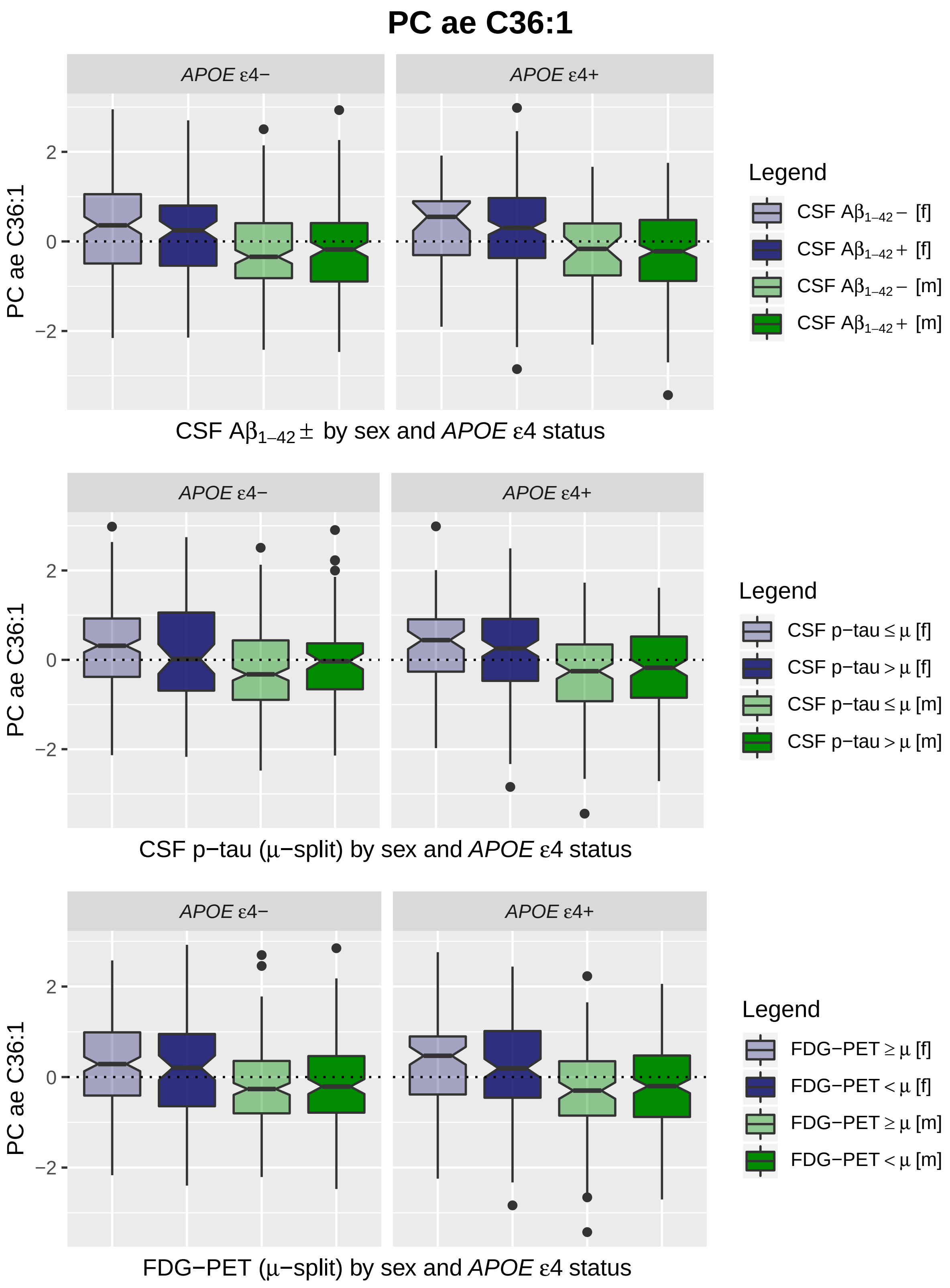
PC ae C36:1
APOE ε4−
APOE ε4+
PC ae C36:1
2
0
−2
Legend
CSF Aβ1–42 − [f]
CSF Aβ1–42 + [f]
CSF Aβ1–42 − [m]
CSF Aβ1–42 + [m]
CSF Aβ1–42 ± by sex and APOE ε4 status
APOE ε4−
APOE ε4+
PC ae C36:1
2
0
−2
Legend
CSF p−tau ≤ μ [f]
CSF p−tau > μ [f]
CSF p−tau ≤ μ [m]
CSF p−tau > μ [m]
CSF p−tau (μ−split) by sex and APOE ε4 status
APOE ε4−
APOE ε4+
PC ae C36:1
2
0
−2
Legend
FDG−PET ≥ μ [f]
FDG−PET < μ [f]
FDG−PET ≥ μ [m]
FDG−PET < μ [m]
FDG−PET (μ−split) by sex and APOE ε4 status

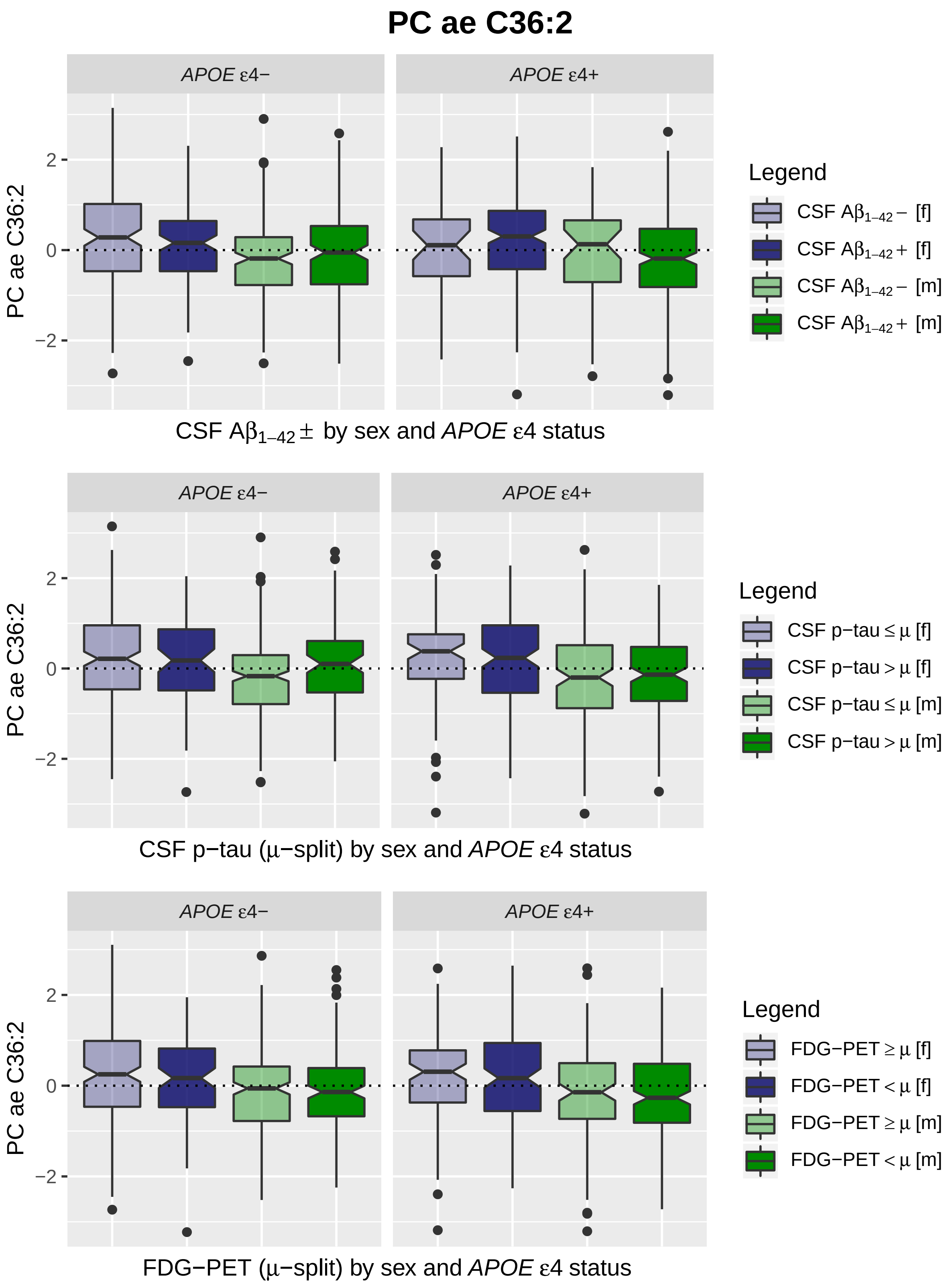
PC ae C36:2
APOE ε4−
APOE ε4+
PC ae C36:2
CSF Aβ1−42 ± by sex and APOE ε4 status
Legend
CSF Aβ1−42 − [f]
CSF Aβ1−42 + [f]
CSF Aβ1−42 − [m]
CSF Aβ1−42 + [m]
CSF p−tau (μ−split) by sex and APOE ε4 status
CSF p−tau ≤ μ [f]
CSF p−tau > μ [f]
CSF p−tau ≤ μ [m]
CSF p−tau > μ [m]
FDG−PET (μ−split) by sex and APOE ε4 status
FDG−PET ≥ μ [f]
FDG−PET < μ [f]
FDG−PET ≥ μ [m]
FDG−PET < μ [m]

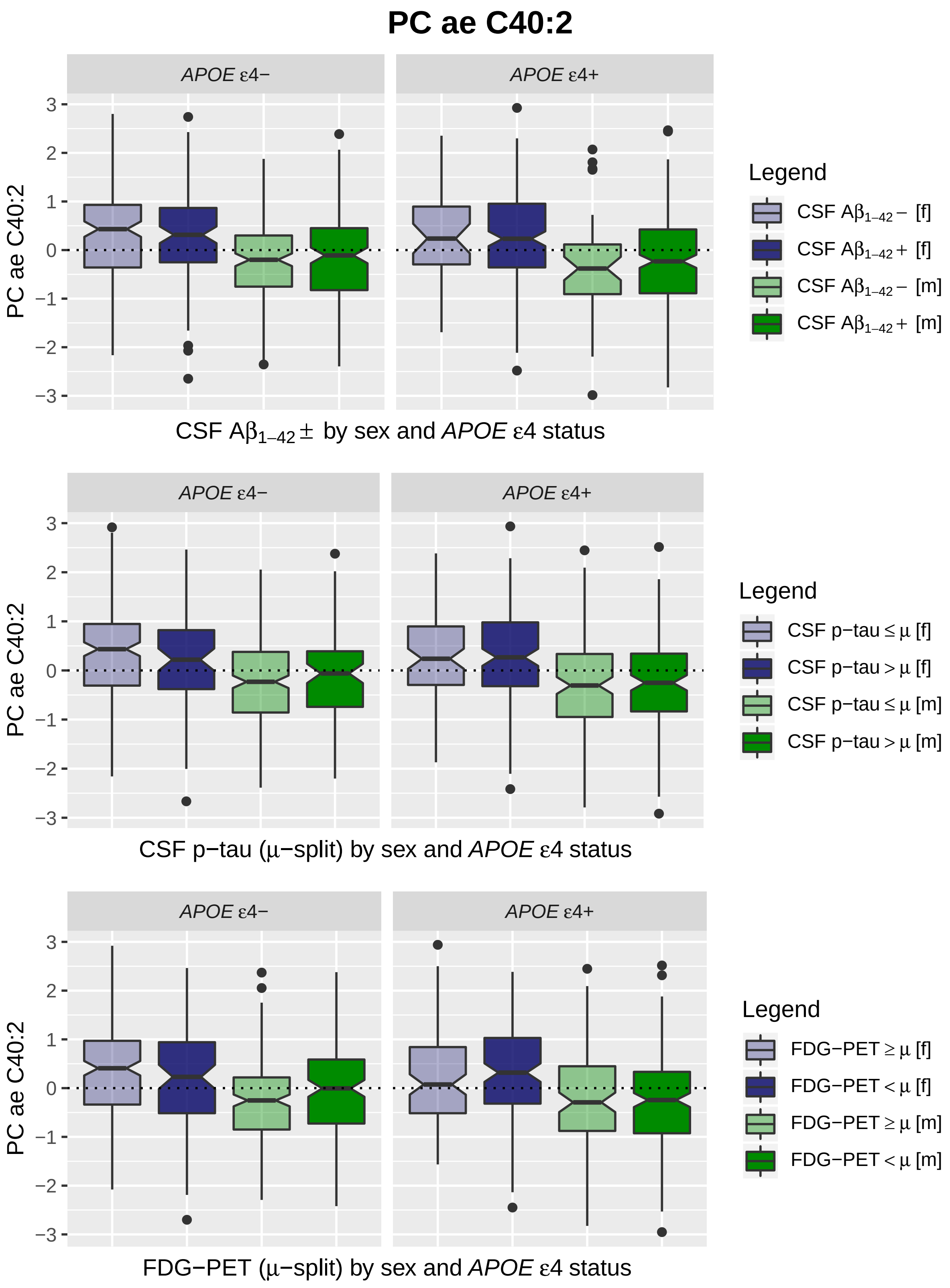
PC ae C40:2
APOE ε4−
APOE ε4+
PC ae C40:2
CSF Aβ1−42 ± by sex and APOE ε4 status
Legend
CSF Aβ1−42 − [f]
CSF Aβ1−42 + [f]
CSF Aβ1−42 − [m]
CSF Aβ1−42 + [m]
CSF p−tau (μ−split) by sex and APOE ε4 status
CSF p−tau ≤ μ [f]
CSF p−tau > μ [f]
CSF p−tau ≤ μ [m]
CSF p−tau > μ [m]
FDG−PET (μ−split) by sex and APOE ε4 status
FDG−PET ≥ μ [f]
FDG−PET < μ [f]
FDG−PET ≥ μ [m]
FDG−PET < μ [m]

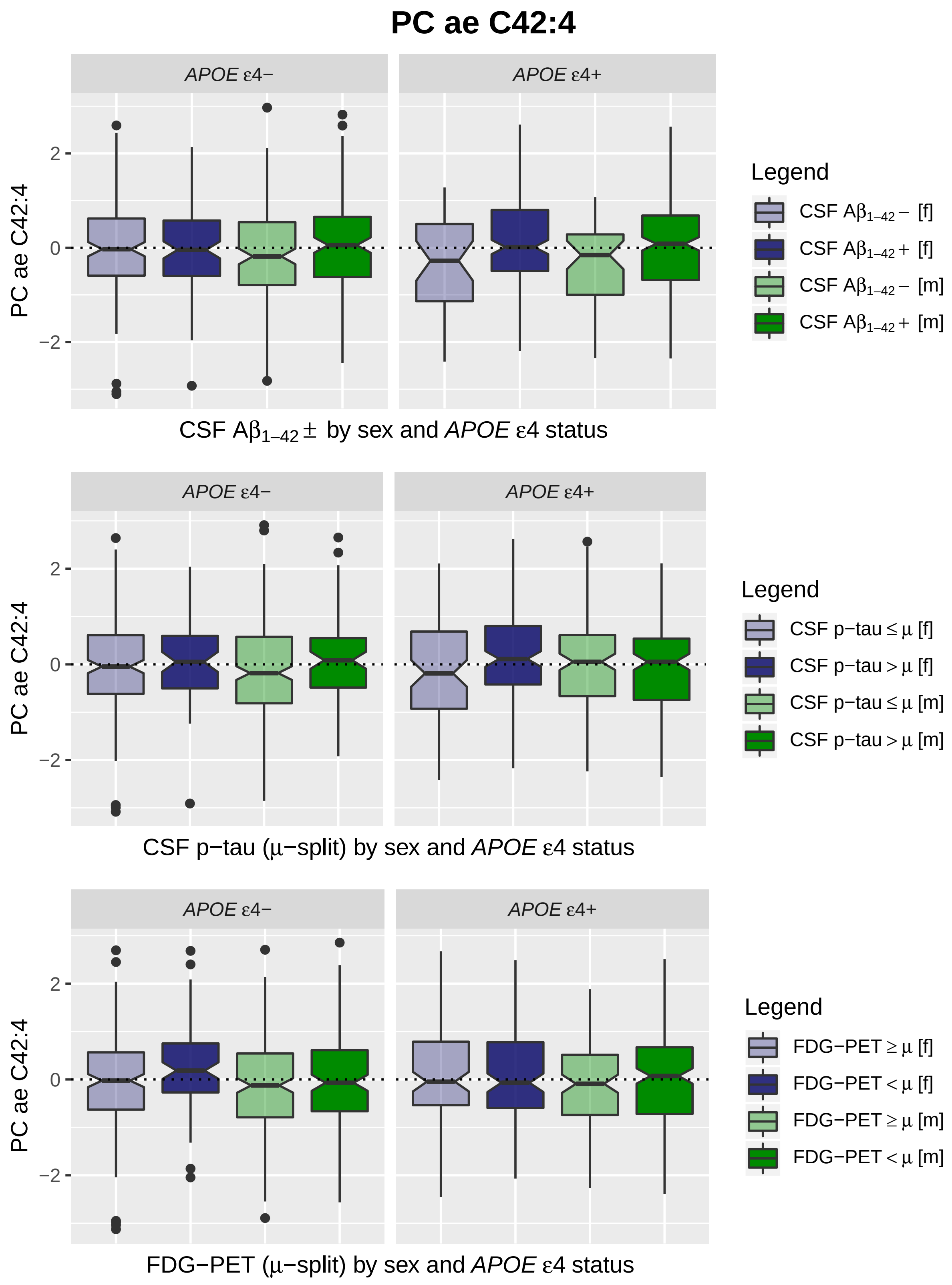
PC ae C42:4
APOE ε4−
APOE ε4+
PC ae C42:4
2
0
−2
CSF Aβ1−42 ± by sex and APOE ε4 status
Legend
CSF Aβ1−42 − [f]
CSF Aβ1−42 + [f]
CSF Aβ1−42 − [m]
CSF Aβ1−42 + [m]
CSF p−tau (μ−split) by sex and APOE ε4 status
Legend
CSF p−tau ≤ μ [f]
CSF p−tau > μ [f]
CSF p−tau ≤ μ [m]
CSF p−tau > μ [m]
FDG−PET (μ−split) by sex and APOE ε4 status
Legend
FDG−PET ≥ μ [f]
FDG−PET < μ [f]
FDG−PET ≥ μ [m]
FDG−PET < μ [m]

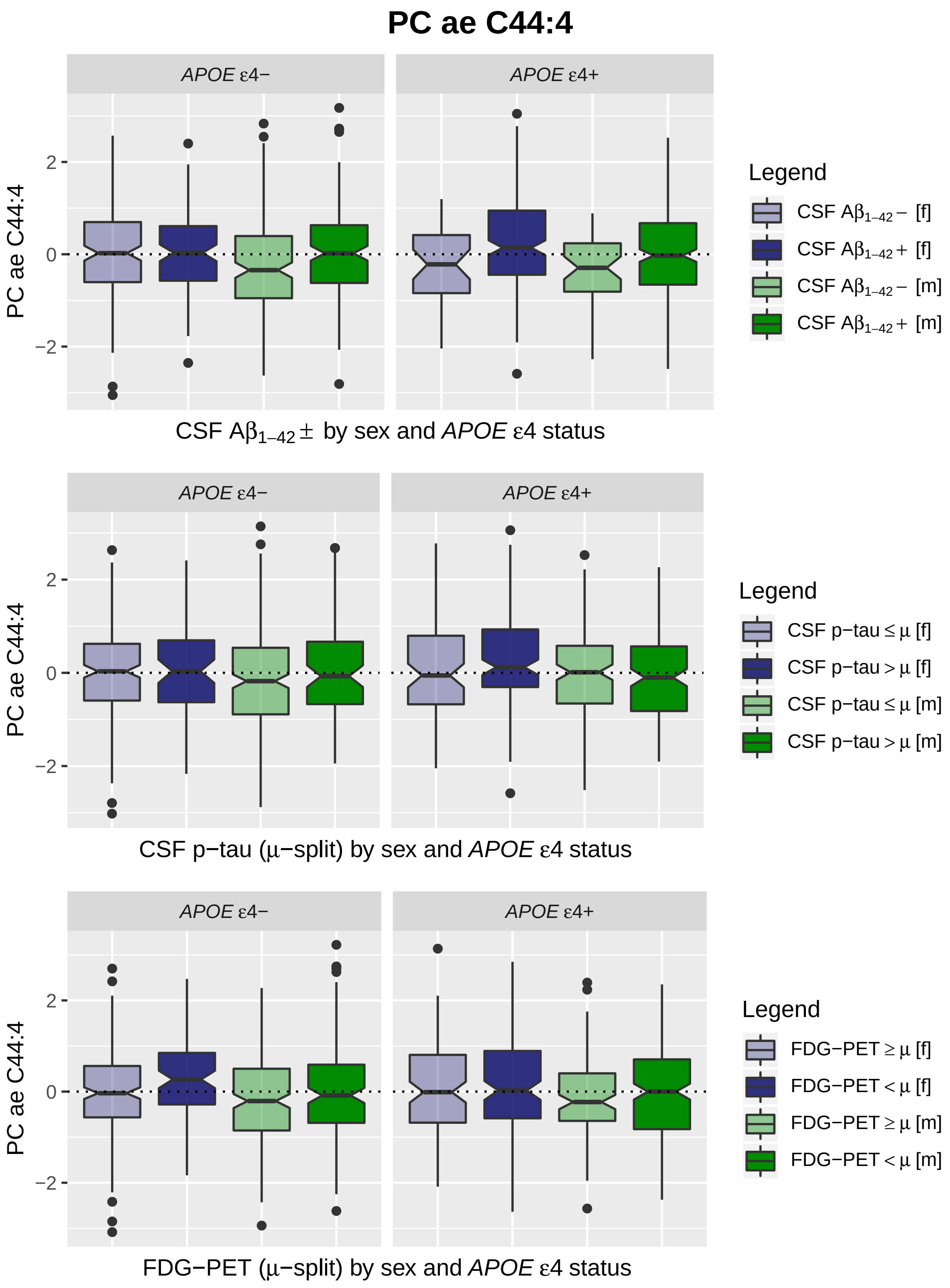
PC ae C44:4
APOE ε4−
APOE ε4+
PC ae C44:4
2
0
−2
CSF Aβ1–42 ± by sex and APOE ε4 status
Legend
CSF Aβ1–42 − [f]
CSF Aβ1–42 + [f]
CSF Aβ1–42 − [m]
CSF Aβ1–42 + [m]
CSF p−tau (μ−split) by sex and APOE ε4 status
CSF p−tau ≤ μ [f]
CSF p−tau > μ [f]
CSF p−tau ≤ μ [m]
CSF p−tau > μ [m]
FDG−PET (μ−split) by sex and APOE ε4 status
FDG−PET ≥ μ [f]
FDG−PET < μ [f]
FDG−PET ≥ μ [m]
FDG−PET < μ [m]

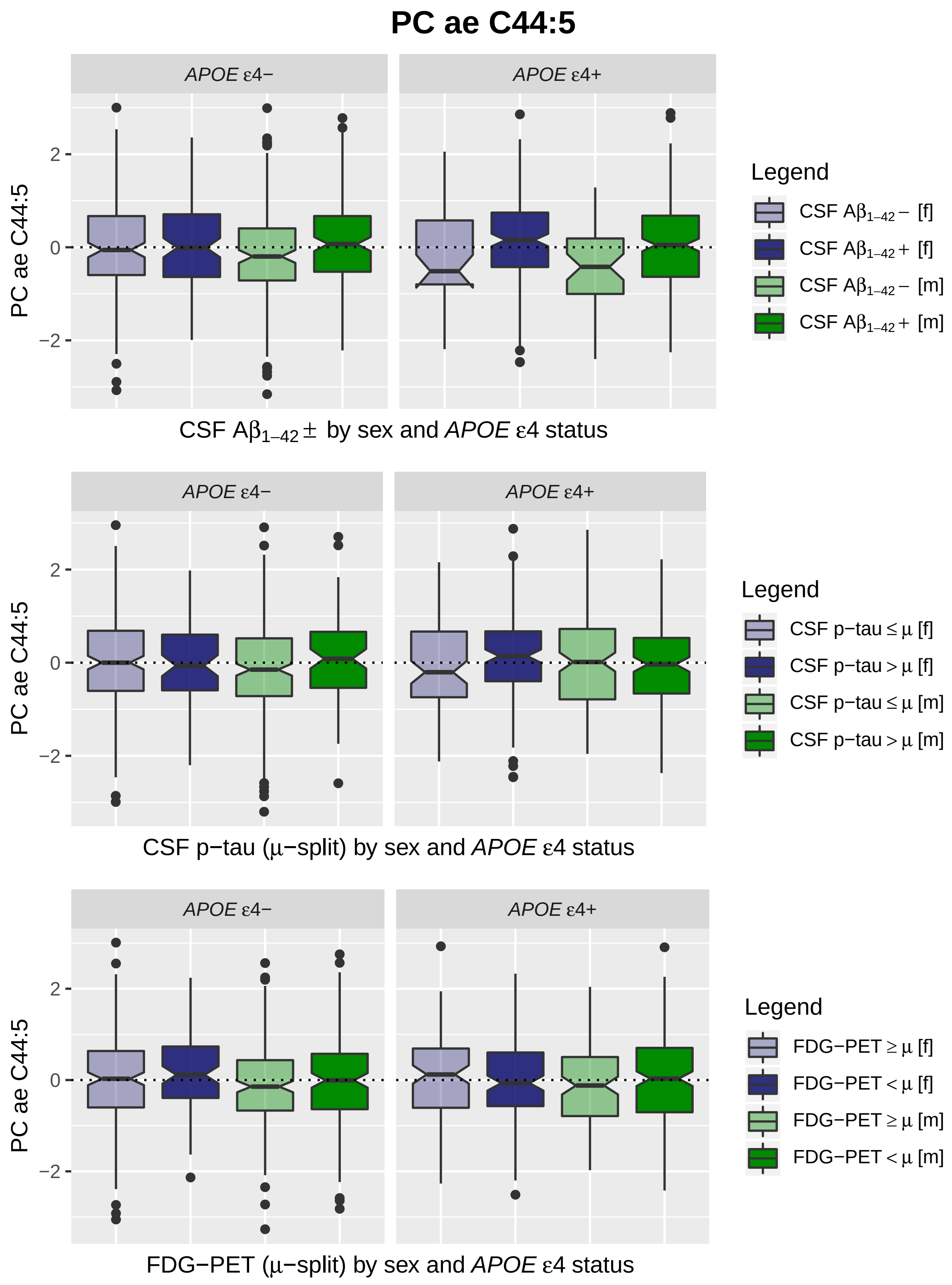

PC ae C44:5
APOE ε4−
APOE ε4+
PC ae C44:5
2
0
−2
Legend
CSF Aβ1–42 − [f]
CSF Aβ1–42 + [f]
CSF Aβ1–42 − [m]
CSF Aβ1–42 + [m]
CSF Aβ1–42 ± by sex and APOE ε4 status
CSF p−tau ≤ μ [f]
CSF p−tau > μ [f]
CSF p−tau ≤ μ [m]
CSF p−tau > μ [m]
CSF p−tau (μ−split) by sex and APOE ε4 status
FDG−PET ≥ μ [f]
FDG−PET < μ [f]
FDG−PET ≥ μ [m]
FDG−PET < μ [m]
FDG−PET (μ−split) by sex and APOE ε4 status

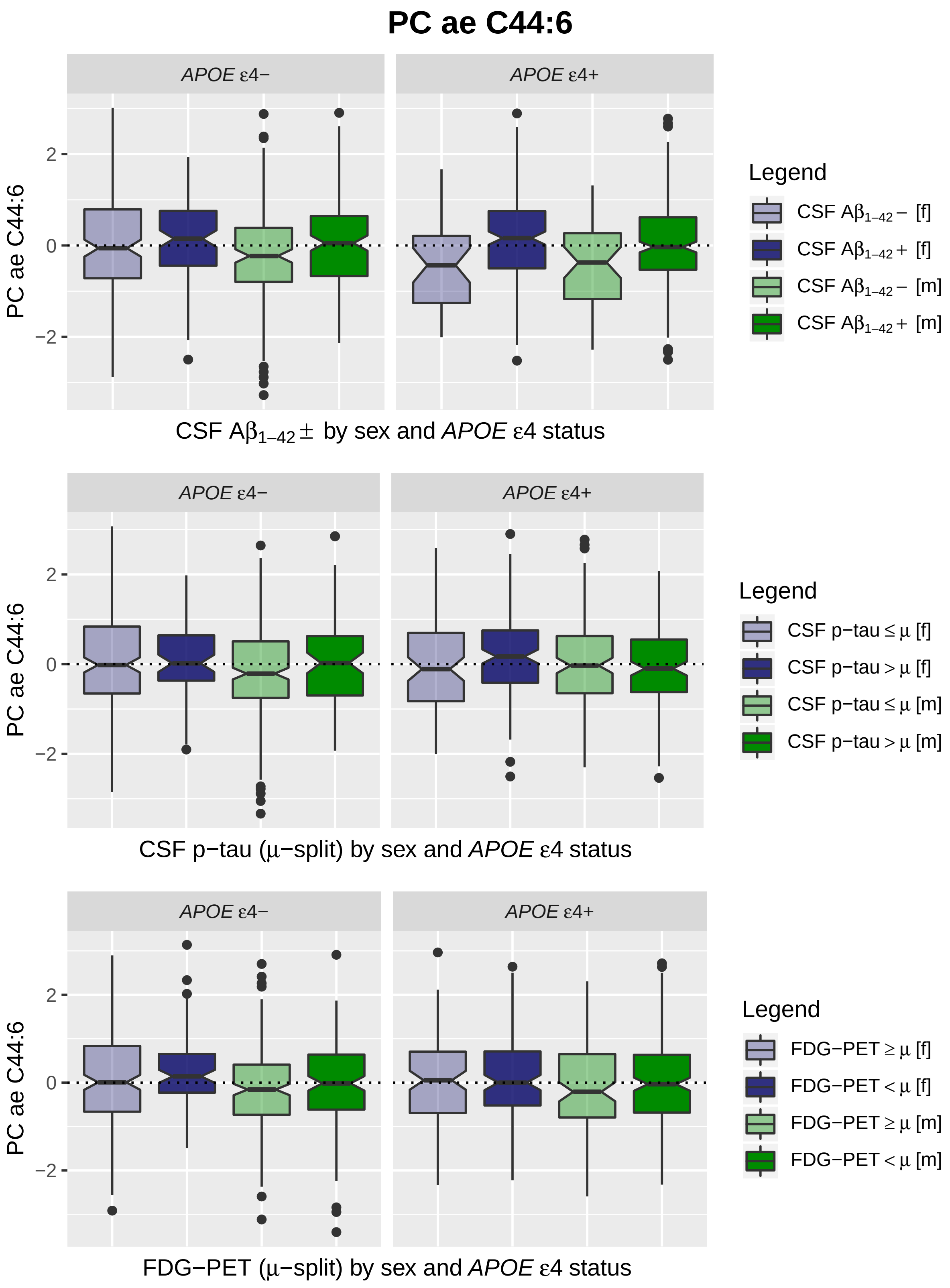

PC ae C44:6
APOE ε4−
APOE ε4+
PC ae C44:6
2
0
−2
Legend
CSF Aβ1–42 − [f]
CSF Aβ1–42 + [f]
CSF Aβ1–42 − [m]
CSF Aβ1–42 + [m]
CSF Aβ1–42 ± by sex and APOE ε4 status
CSF p−tau ≤ μ [f]
CSF p−tau > μ [f]
CSF p−tau ≤ μ [m]
CSF p−tau > μ [m]
CSF p−tau (μ−split) by sex and APOE ε4 status
FDG−PET ≥ μ [f]
FDG−PET < μ [f]
FDG−PET ≥ μ [m]
FDG−PET < μ [m]
FDG−PET (μ−split) by sex and APOE ε4 status

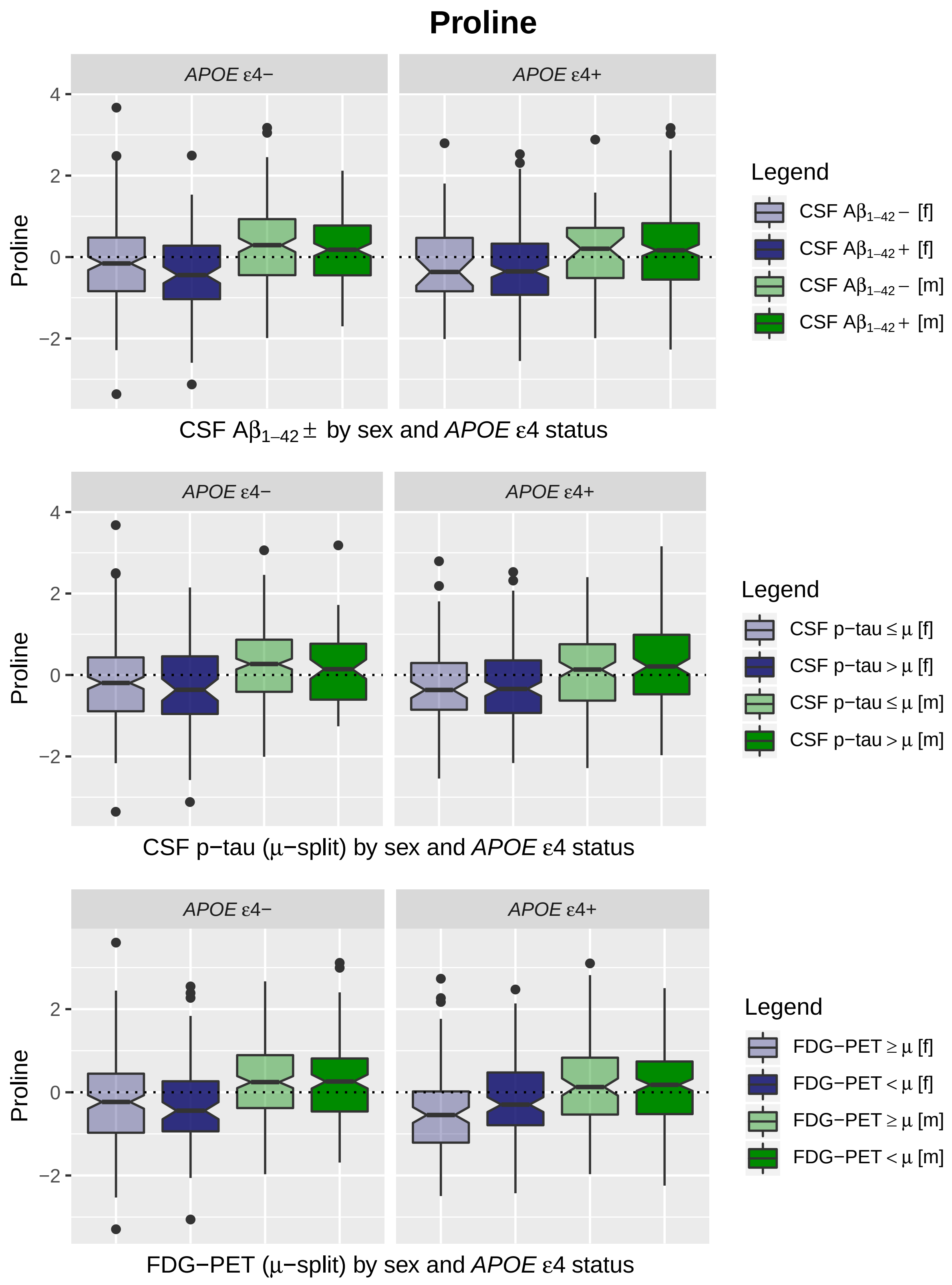
Proline
APOE ε4−
APOE ε4+
Proline
4
2
0
−2
Legend
CSF Aβ1–42 − [f]
CSF Aβ1–42 + [f]
CSF Aβ1–42 − [m]
CSF Aβ1–42 + [m]
CSF Aβ1–42 ± by sex and APOE ε4 status
APOE ε4−
APOE ε4+
Proline
Legend
CSF p−tau ≤ μ [f]
CSF p−tau > μ [f]
CSF p−tau ≤ μ [m]
CSF p−tau > μ [m]
CSF p−tau (μ−split) by sex and APOE ε4 status
APOE ε4−
APOE ε4+
Proline
Legend
FDG−PET ≥ μ [f]
FDG−PET < μ [f]
FDG−PET ≥ μ [m]
FDG−PET < μ [m]
FDG−PET (μ−split) by sex and APOE ε4 status

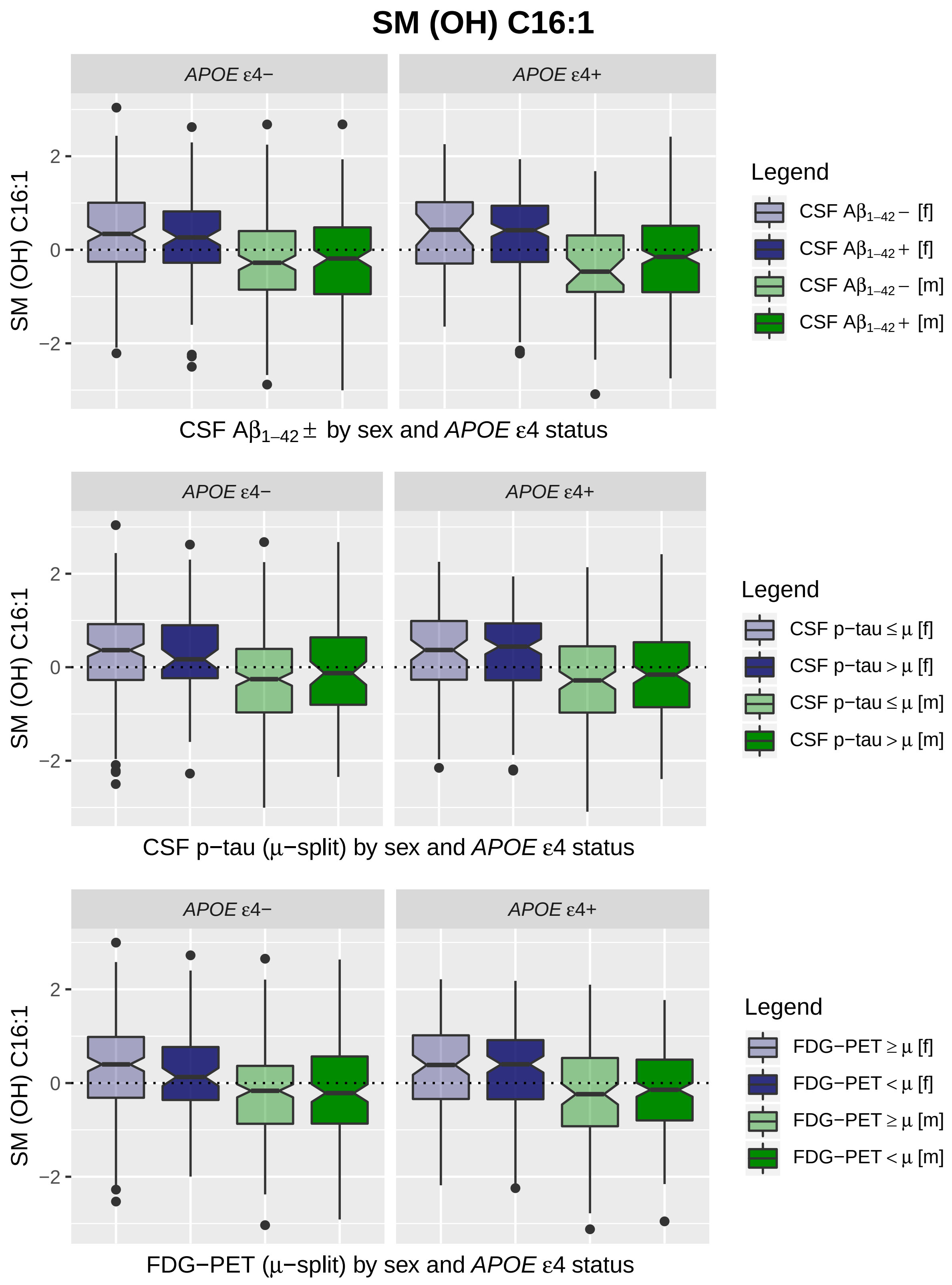
SM (OH) C16:1
APOE ε4−
APOE ε4+
SM (OH) C16:1
2
0
−2
Legend
CSF Aβ1−42 − [f]
CSF Aβ1−42 + [f]
CSF Aβ1−42 − [m]
CSF Aβ1−42 + [m]
CSF Aβ1−42 ± by sex and APOE ε4 status
CSF p−tau ≤ μ [f]
CSF p−tau > μ [f]
CSF p−tau ≤ μ [m]
CSF p−tau > μ [m]
CSF p−tau (μ−split) by sex and APOE ε4 status
FDG−PET ≥ μ [f]
FDG−PET < μ [f]
FDG−PET ≥ μ [m]
FDG−PET < μ [m]
FDG−PET (μ−split) by sex and APOE ε4 status

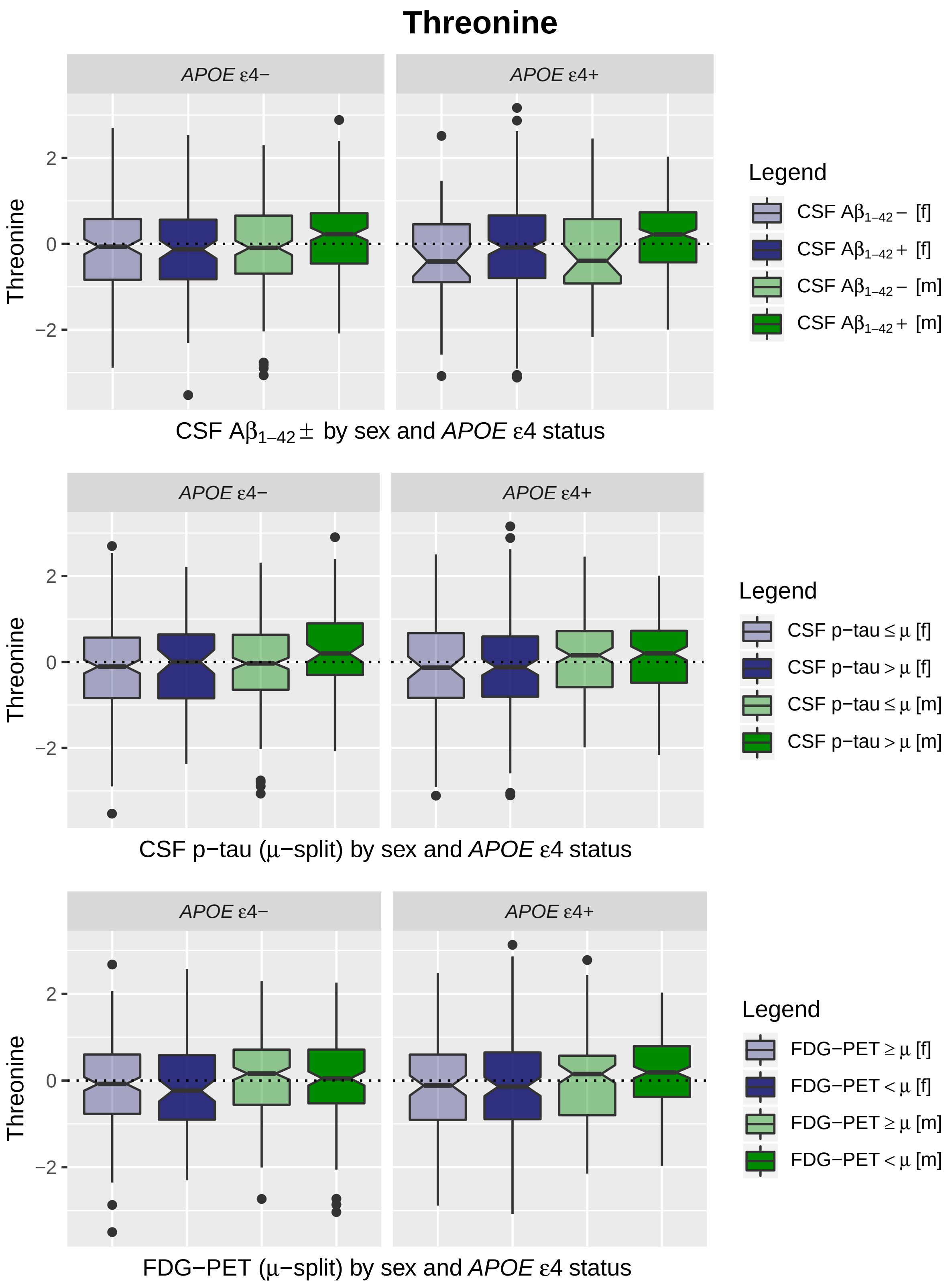

Threonine
APOE ε4−
APOE ε4+
Threonine
2
0
−2
Legend
CSF Aβ1–42 − [f]
CSF Aβ1–42 + [f]
CSF Aβ1–42 − [m]
CSF Aβ1–42 + [m]
CSF Aβ1–42 ± by sex and APOE ε4 status
APOE ε4−
APOE ε4+
Threonine
2
0
−2
Legend
CSF p−tau ≤ μ [f]
CSF p−tau > μ [f]
CSF p−tau ≤ μ [m]
CSF p−tau > μ [m]
CSF p−tau (μ−split) by sex and APOE ε4 status
APOE ε4−
APOE ε4+
Threonine
2
0
−2
Legend
FDG−PET ≥ μ [f]
FDG−PET < μ [f]
FDG−PET ≥ μ [m]
FDG−PET < μ [m]
FDG−PET (μ−split) by sex and APOE ε4 status

Valine

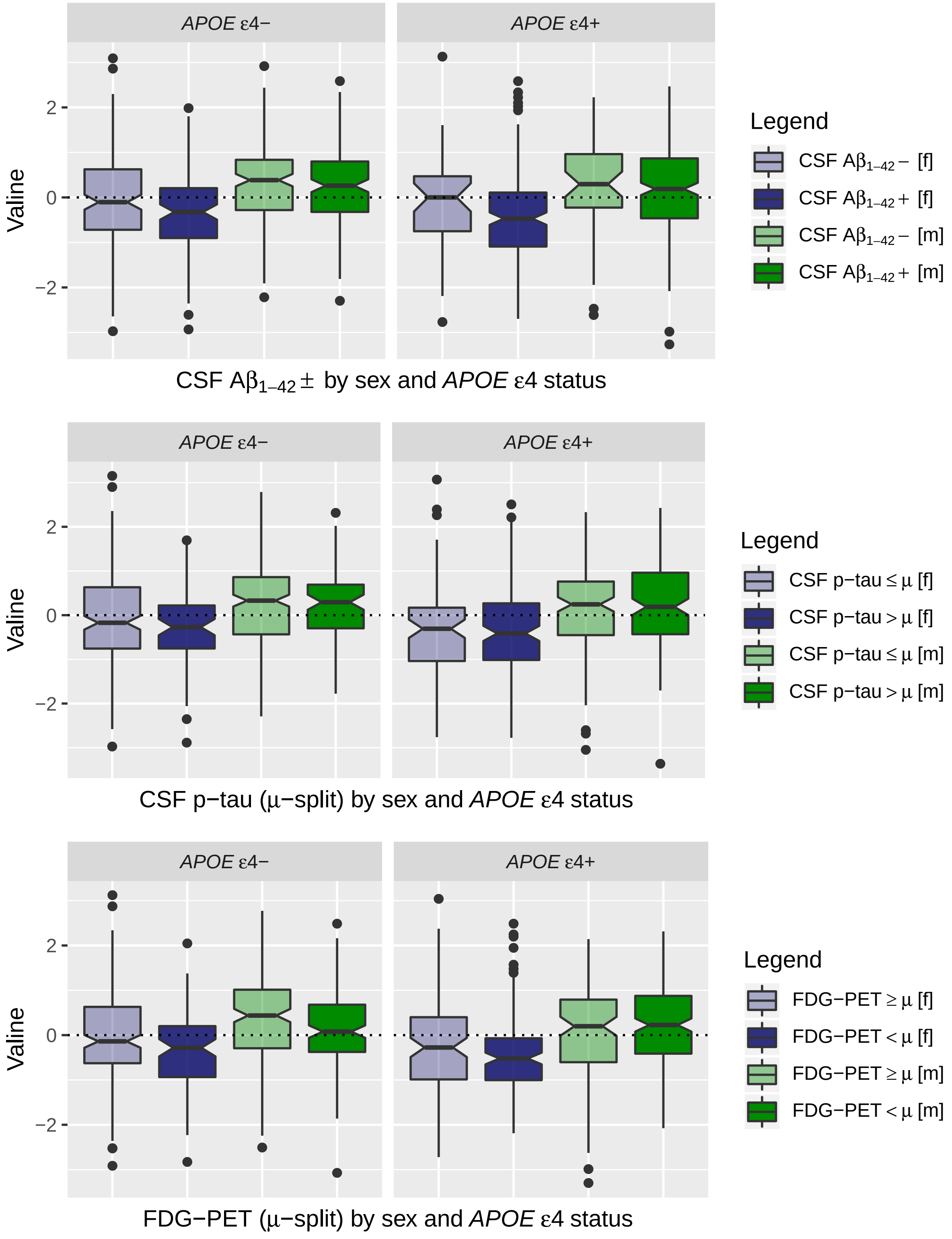
APOE ε4−
APOE ε4+
Valine
2
0
−2
Legend
CSF Aβ1–42 − [f]
CSF Aβ1–42 + [f]
CSF Aβ1–42 − [m]
CSF Aβ1–42 + [m]
CSF Aβ1–42 ± by sex and APOE ε4 status
CSF p−tau ≤ μ [f]
CSF p−tau > μ [f]
CSF p−tau ≤ μ [m]
CSF p−tau > μ [m]
CSF p−tau (μ−split) by sex and APOE ε4 status
FDG−PET ≥ μ [f]
FDG−PET < μ [f]
FDG−PET ≥ μ [m]
FDG−PET < μ [m]
FDG−PET (μ−split) by sex and APOE ε4 status

Supplementary Figure 5: Quotient normalization for batch removal exemplified for PC ae C44:5

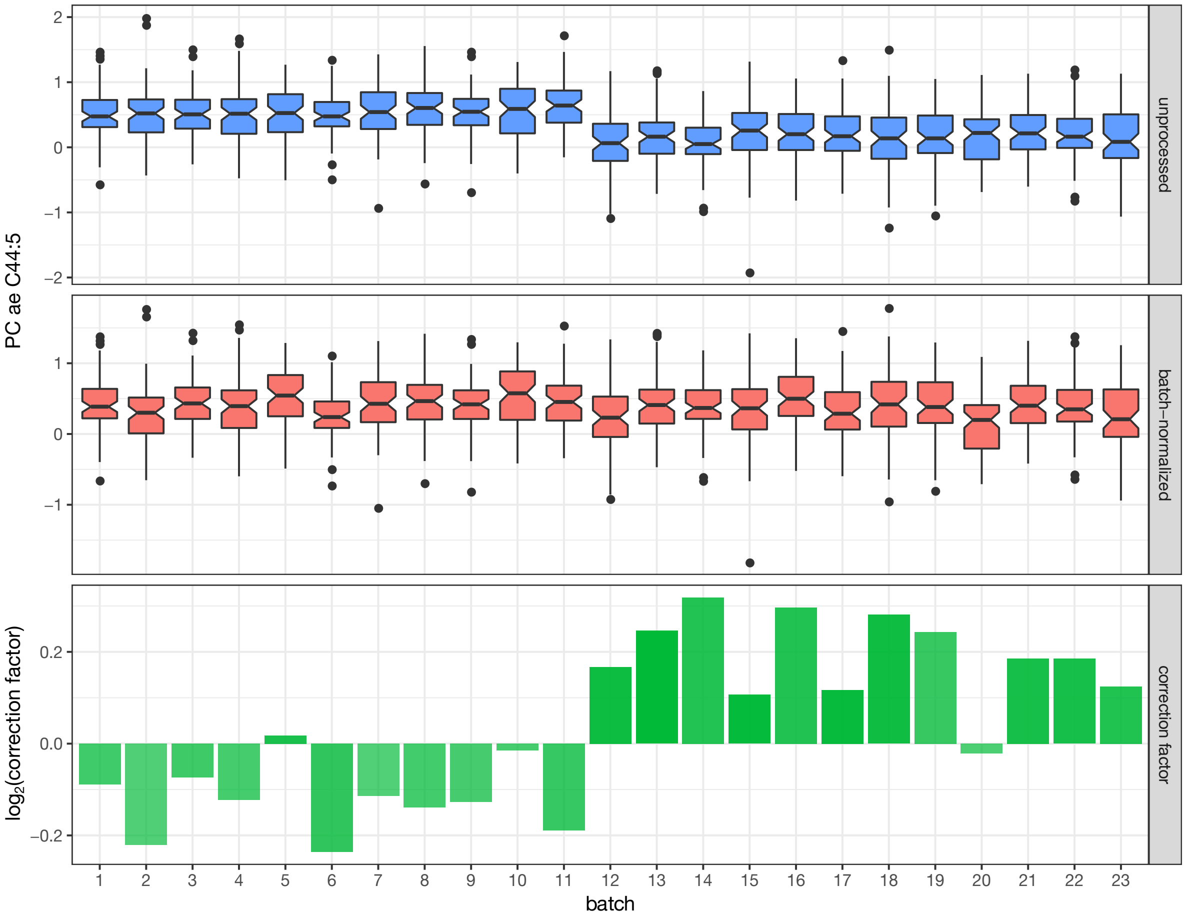

Supplementary Figure 5: Quotient normalization for batch removal exemplified for PC ae C44:5. The **top panel** shows boxplots of log_2-transformed levels of PC ae C44:5 in study samples for all batches (defined by 96-well plates) prior to quotient normalization. Differences between plates as well as between runs for ADNI-1 (batches 1-11) vs. ADNI-GO/2 (batches 12-23) are clearly identifiable. The **middle panel** shows log_2-transformed levels of PC ae C44:5 in study samples for all batches after quotient normalization using measurements for NIST standards. The shift between ADNI-1 and -GO/2 is not significant any more, however, differences between single batches are still observable. As measurements for NIST standards are assumed to be stable across plates, the remaining variability should be biological variance due to random distribution of study samples across plates. To ascertain that this is the case, i.e. that technical confounds are removed but true biological variance is retained, we perform additional QC steps using blinded duplicates/triplicates of study samples distributed across batches and remove metabolites that show excessive values for the coefficient of variation and the intraclass correlation coefficient. The **bottom panel** shows the log_2-transformed (for clearer visualization) correction factors. These correction or dilution factors are obtained for each batch and metabolite by dividing the global, cross-batch mean concentration of a metabolite in NIST standards by the within-batch mean concentration in NIST standards of the same metabolite. Metabolite concentrations in study samples are then multiplied by the factor derived for the batch a study sample was contained in.

Supplementary Figure 6: Distributions of levels of all 139 metabolites included after QC.

Histograms of metabolite distributions after QC, log_2-transformation and centering/scaling.

Acylcarnitines

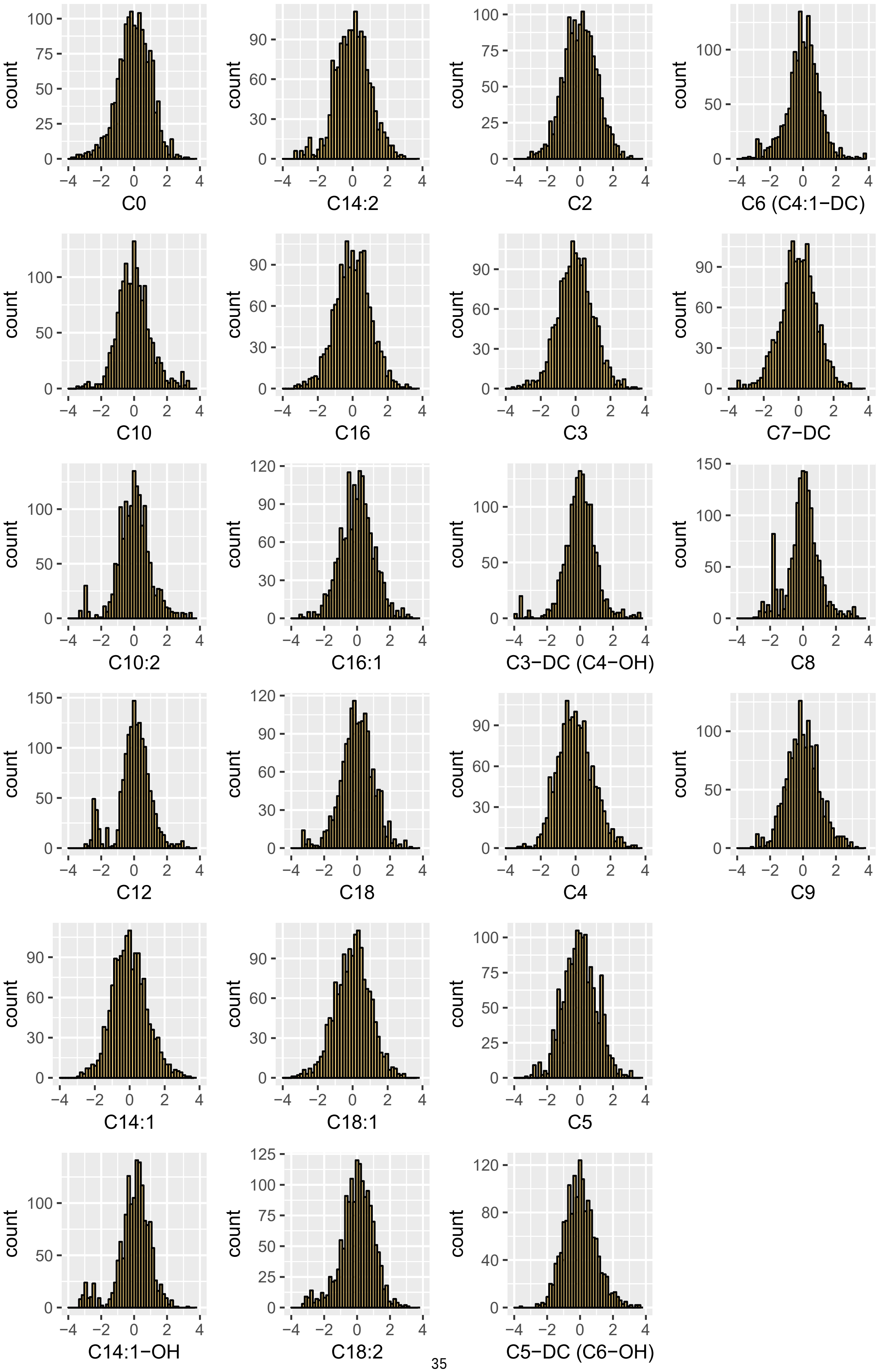

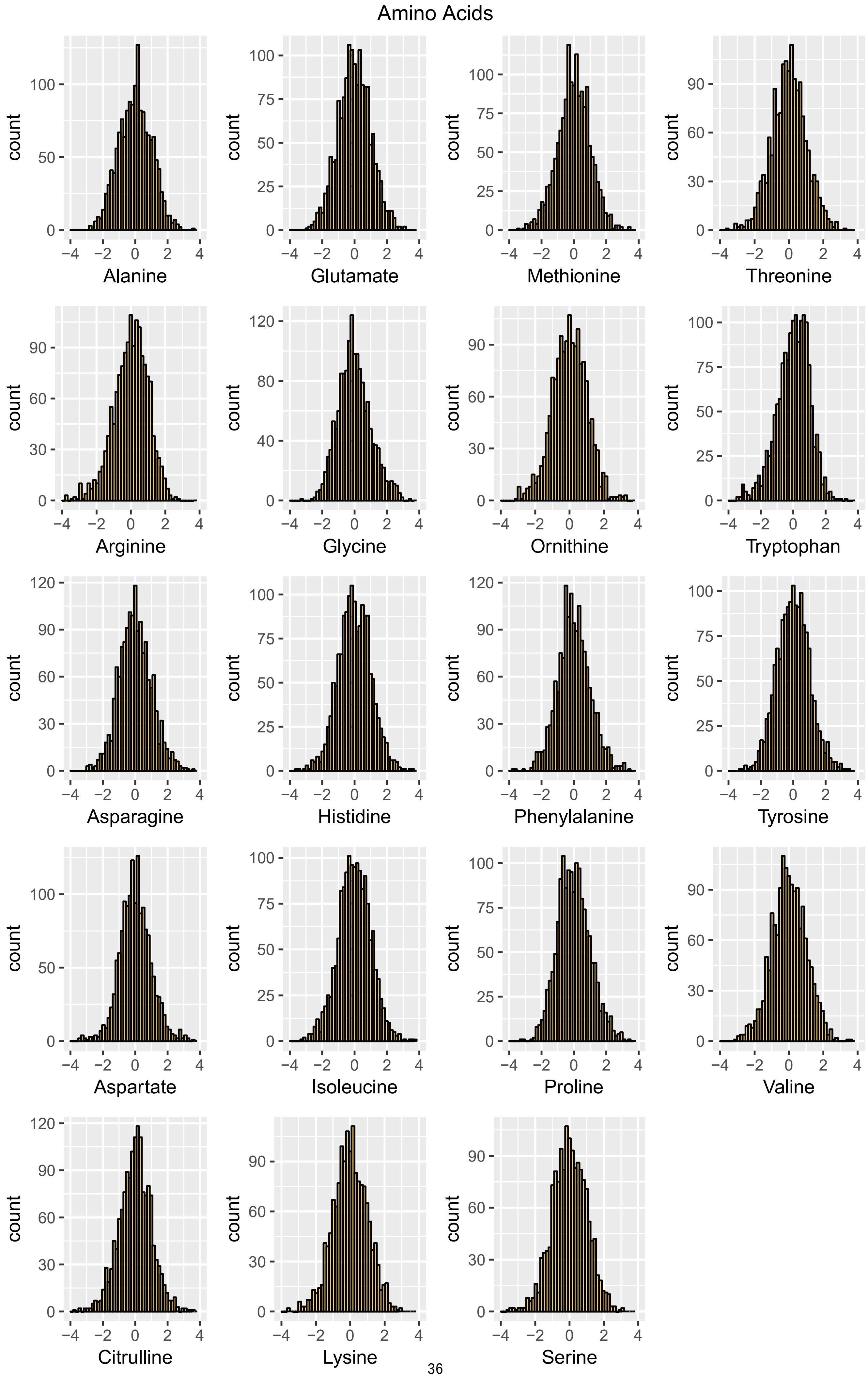
Amino Acids
count
Alanine
Glutamate
Methionine
Threonine
Arginine
Glycine
Ornithine
Tryptophan
Asparagine
Histidine
Phenylalanine
Tyrosine
Aspartate
Isoleucine
Proline
Valine
Citrulline
Lysine
Serine

Biogenic Amines

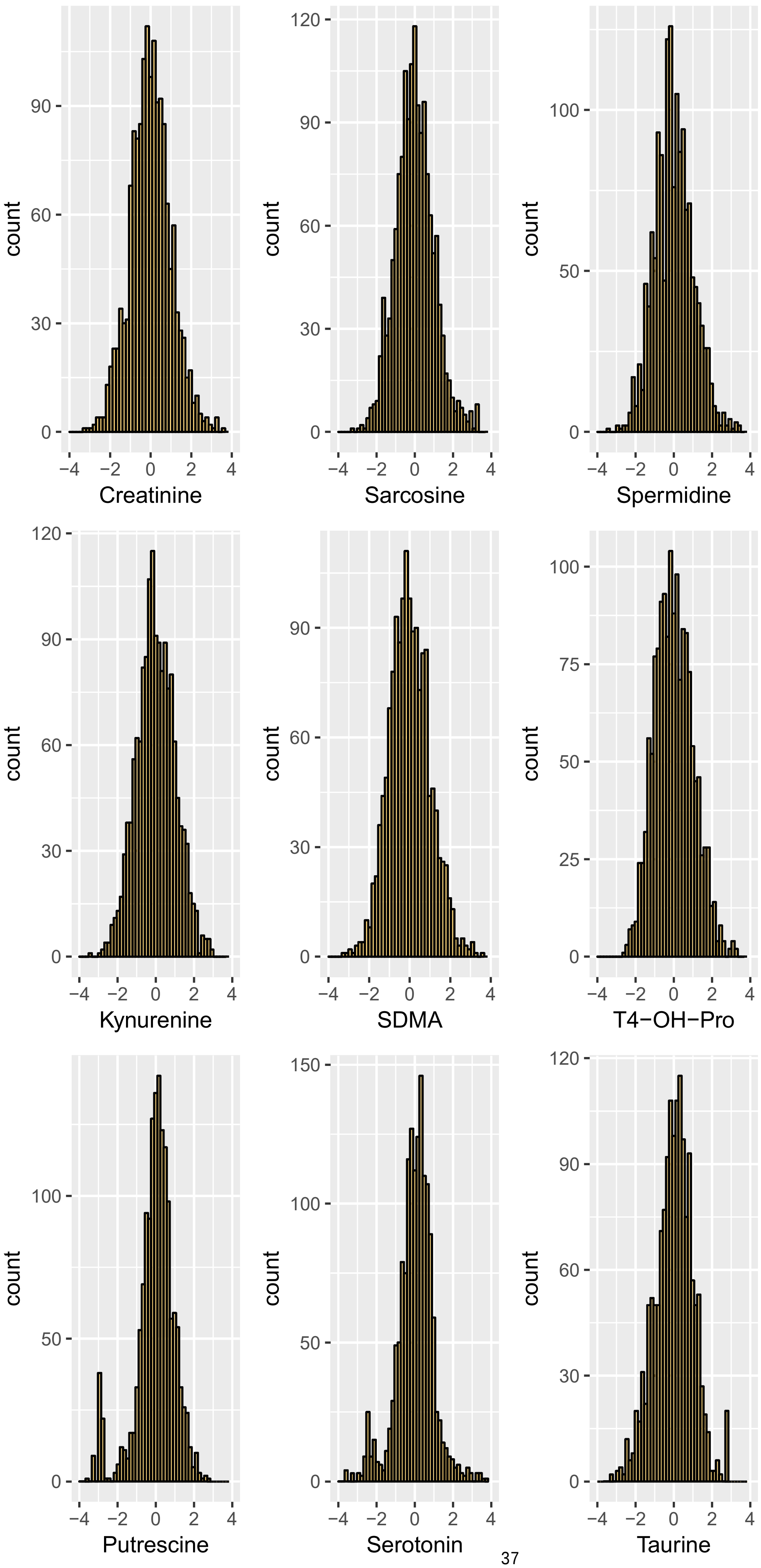

Glycerophospholipids

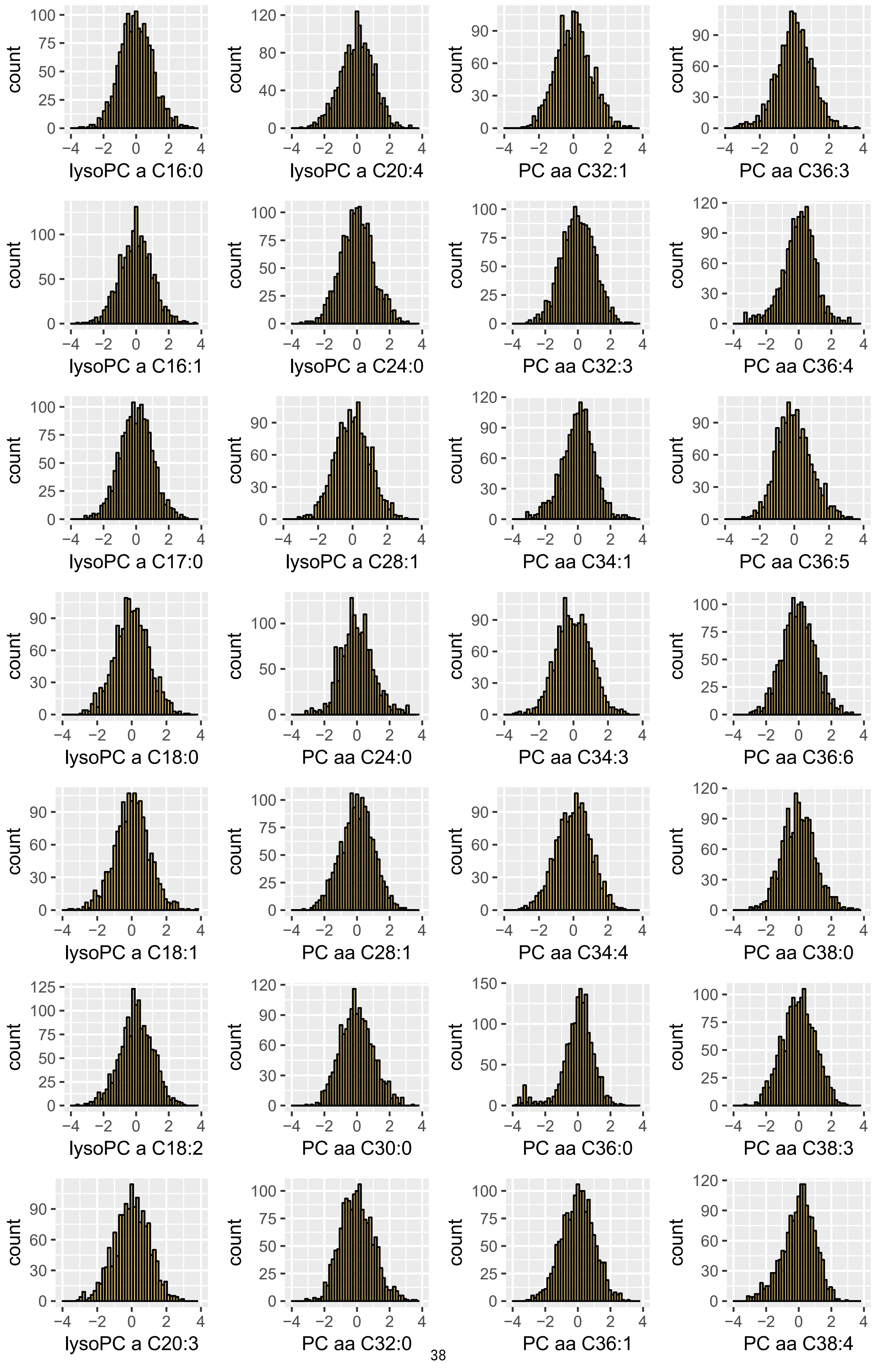

Glycerophospholipids

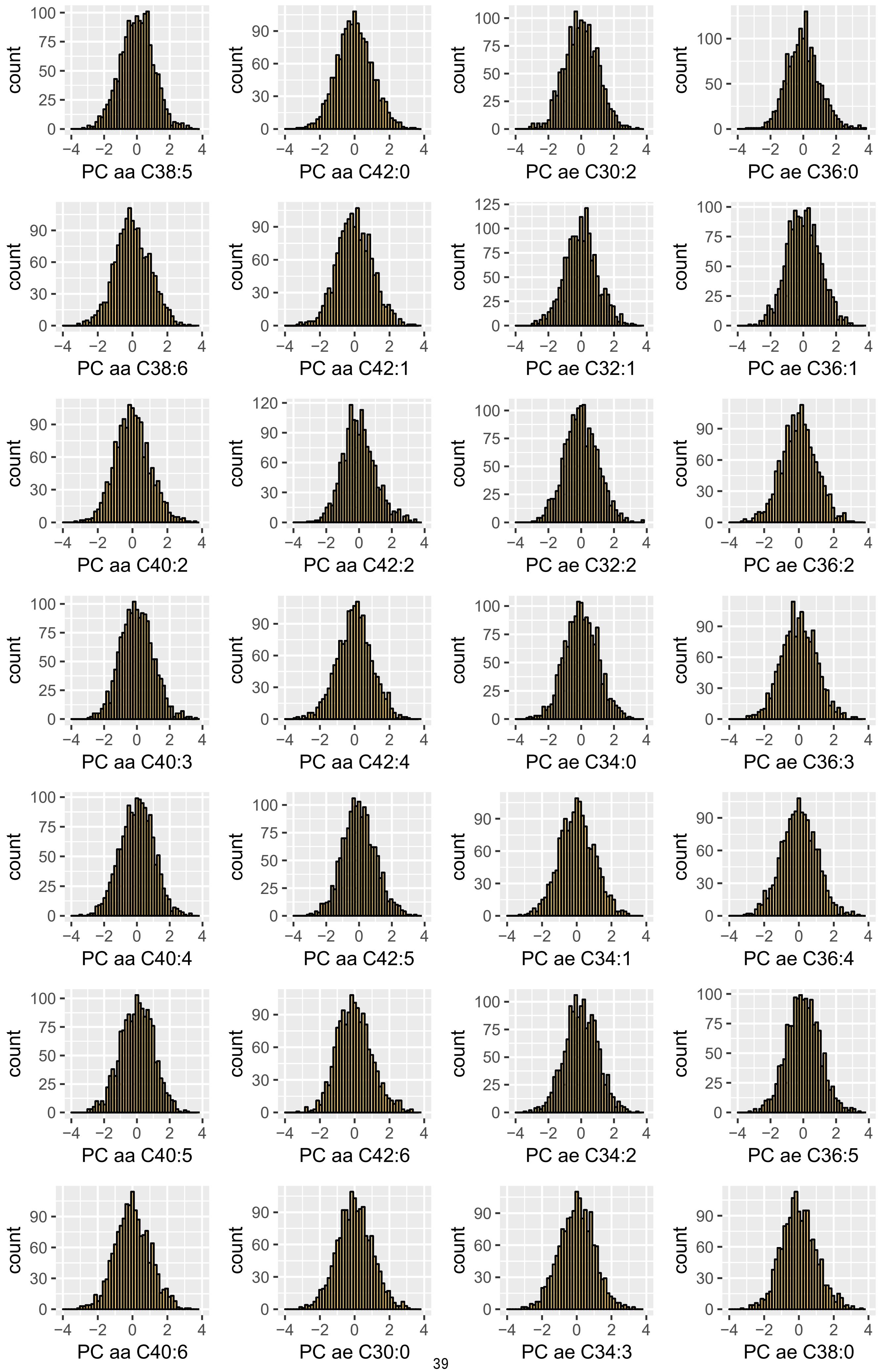

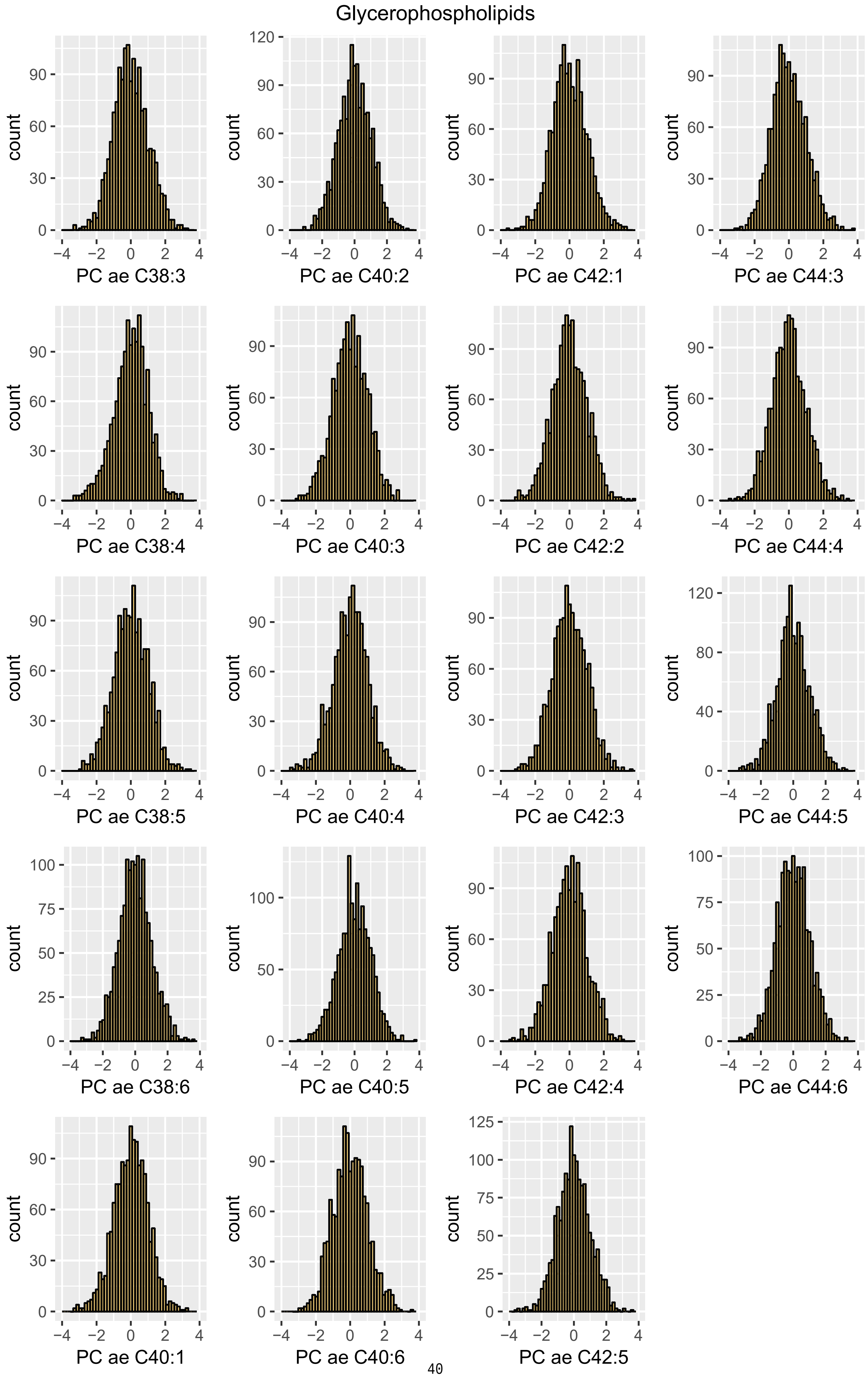
Glycerophospholipids
count
PC ae C38:3
PC ae C40:2
PC ae C42:1
PC ae C44:3
PC ae C38:4
PC ae C40:3
PC ae C42:2
PC ae C44:4
PC ae C38:5
PC ae C40:4
PC ae C42:3
PC ae C44:5
PC ae C38:6
PC ae C40:5
PC ae C42:4
PC ae C44:6
PC ae C40:1
PC ae C40:6
PC ae C42:5

Sphingolipids

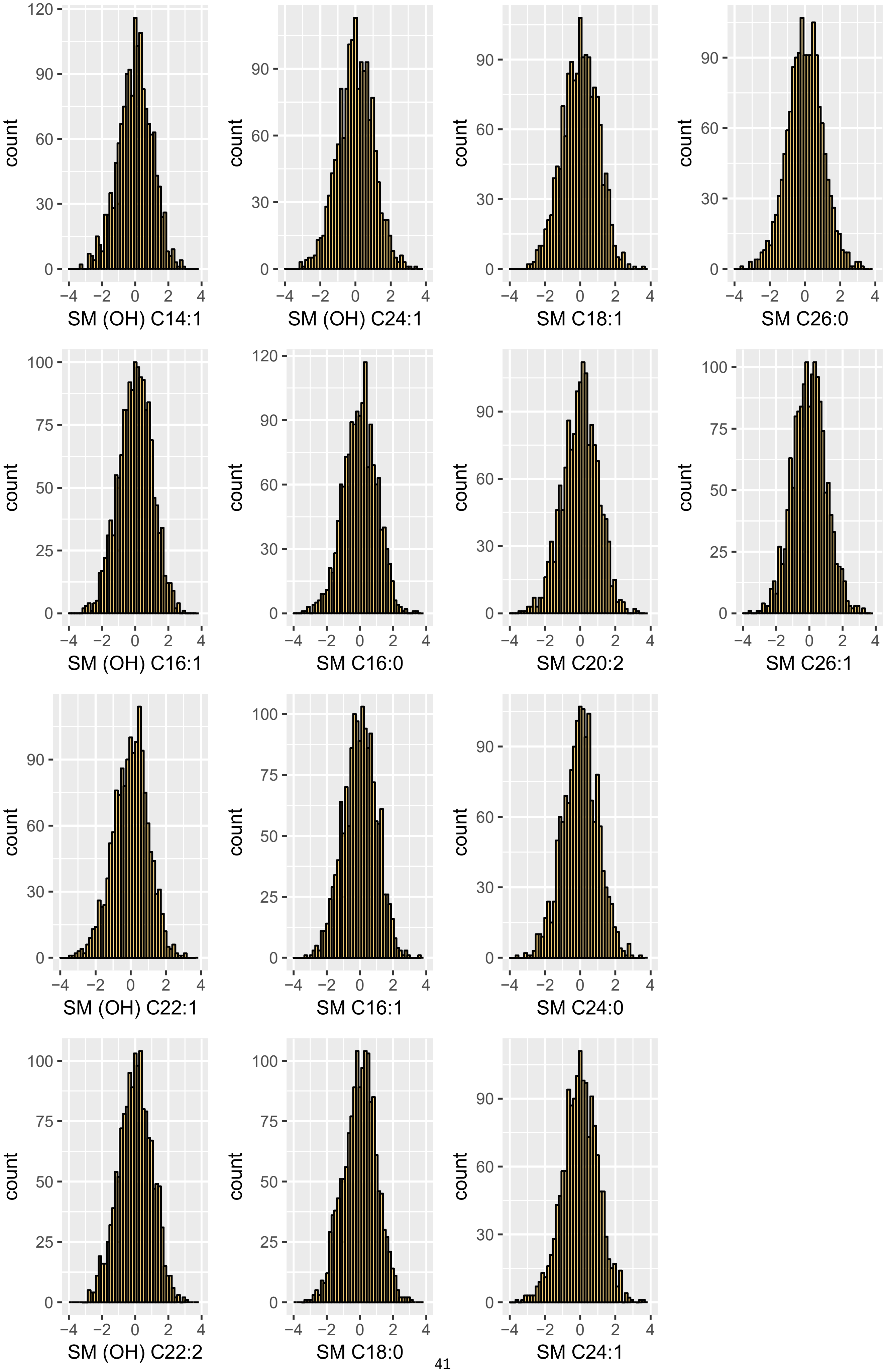

Supplement: Supplementary file 1 — Supplementary Information [file 41467_2020_14959_MOESM1_ESM.pdf]
