# Supplementary material for: Sex and APOE ε4 genotype modify the Alzheimer’s disease serum metabolome
Source: Nat Commun. 2020 Mar 2;11:1148. doi: 10.1038/s41467-020-14959-w (PMC7052223; doi:10.1038/s41467-020-14959-w)
Supplement: Supplementary file 3 — Description of Additional Supplementary Files [file 41467_2020_14959_MOESM3_ESM.pdf]

Description of Additional Supplementary Files

File Name: Supplementary Data 1
Description: Metabolic imprint of sex on blood metabolites in ADNI cohorts for all participants and stratified by diagnostic groups (CN, LMCI, and probable AD).
Lists the metabolites and their pathways, and for each analysis information on the peripheral metabolic sex-differences observed, the absolute difference in means between sexes, and the associated P-value. For diagnosis-stratified analyses, we additionally provide an estimate of power to observe the differences seen in all subjects in the respective stratum in a two-sample t-test. Further, estimates of heterogeneity for metabolic sex-differences between CN subjects and participants with probable AD are provided, as well as the results from the Mittelstrass et al. study of metabolic sex-differences in the population-based KORA cohort.

File Name: Supplementary Data 2
Description: Full association results for metabolites with A-T-N biomarkers having any Pvalue < 0.05 in the pooled analysis, the sex-stratified analysis, or the APOE ε4 status-stratified analysis (including heterogeneity estimates). Association results are provided in the same format as for Table 2 and Table 3 in the main manuscript.

File Name: Supplementary Data 3
Description: Results of the bootstrapping analysis (n = 1000) of all reported findings in ADNI.

File Name: Supplementary Data 4
Description: Association results from ROS/MAP for brain amyloid and tau pathology in relation to 17 metabolites. Association results are provided in the same format as for Table 2 and Table 3 in the main manuscript. Significant (at Bonferroni significance for 17 unique metabolites, i.e. 2.94 x 10-3) associations are marked in bold.

File Name: Supplementary Data 5
Description: Association results from the AIBL cohort for CSF p-tau in relation to 3 metabolites. Association results are provided in the same format as for Table 2 and Table 3 in the main manuscript.

File Name: Supplementary Data 6
Description: Quality control measures for all metabolites with data available in this study. Lists for each metabolite the intra-class correlation coefficient (ICC) and the coefficient of variation (CV) in blinded duplicates/triplicates, as well as the rate of missing data. The last column marks metabolites as “passed” for included metabolites, and “failed” for excluded metabolites.
