# Supplementary material for: Sex and APOE ε4 genotype modify the Alzheimer’s disease serum metabolome
Source: Nat Commun. 2020 Mar 2;11:1148. doi: 10.1038/s41467-020-14959-w (PMC7052223; doi:10.1038/s41467-020-14959-w)
Supplement: Supplementary file 6 — Supplementary Data 3 [file 41467_2020_14959_MOESM6_ESM.pdf]

Supplementary Data 1 - Bootstrapping analysis

for: Sex and *APOE* ε4 genotype modify the Alzheimer's disease serum metabolome

Matthias Arnold, Kwangsik Nho, Alexandra Kueider-Paisley, Tyler Massaro, Kevin Huynh, Barbara Brauner, Siamak MahmoudianDehkordi, Gregory Louie, M. Arthur Moseley, J. Will Thompson, Lisa St John Williams, Jessica D. Tenenbaum, Colette Blach, Rui Chang, Roberta D. Brinton, Rebecca Baillie, Xianlin Han, John Q. Trojanowski, Leslie M. Shaw, Ralph Martins, Michael W. Weiner, Eugenia Trushina, Jon B. Toledo, Peter J. Meikle, David A. Bennett, Jan Krumsiek, P. Murali Doraiswamy, Andrew J. Saykin, Rima Kaddurah-Daouk, and Gabi Kastenmüller for the Alzheimer's Disease Neuroimaging Initiative and the Alzheimer's Disease Metabolomics Consortium

Contents

Sex-stratified analysis . . . **2**
- **Pathological CSF A$\beta_{1\text{-}42}$** . . . 2
- **CSF p-tau** . . . 4
- **FDG-PET** . . . 9

***APOE* ε4 status-stratified analyses** . . . **13**
- **Pathological CSF A$\beta_{1\text{-}42}$** . . . 13
- **FDG-PET** . . . 16

2-fold stratified analyses (sex * *APOE* ε4 status) . . . **21**
- **Pathological CSF A$\beta_{1\text{-}42}$** . . . 21
- **CSF p-tau** . . . 22
- **FDG-PET** . . . 23

β_0: original beta (as reported in manuscript); P_0: original P-value; bias (β_0): bias of the original beta as determined by 1000 bootstraps; β_{boot}: histogram of β-estimates across 1000 bootstraps, with β_0 indicated as dashed line and 95% bootstrap-t confidence intervals given by grey lines; P_N: P-value of the Shapiro-Wilk test for normality; $\mu(\beta)_{boot}$: mean β across the 1000 bootstraps; $\mu(SE)_{boot}$: mean of the standard error of β across the 1000 bootstraps; P_{boot}: two-tailed P-value of the standard normal distribution for –abs($\mu(\beta)_{boot}$ / $\mu(SE)_{boot}$); % $P_{bonf.sign.}$: fraction of bootstraps yielding a Bonferroni-significant P-value ($P \leq 9.09 \times 10^{-4}$); % $P_{nom.sign.}$: fraction of bootstraps yielding a nominally significant P-value ($P \leq 0.05$); Δ Sex / Δ APOE ε4±: statistics for heterogeneity (P_{boot}: two-tailed P-value of the standard normal distribution for the mean heterogeneity estimate; % $P_{nom.sign.}$: fraction of bootstraps yielding a nominally significant P-value; I^2_{boot}: I^2 calculated for the mean heterogeneity estimate; % $I^2_{boot} = 0$: fraction of bootstraps yielding an I^2 estimate of exactly zero)

Sex-stratified analysis

Pathological CSF A$\beta_{1\text{-}42}$

metabolite	statistic	pooled	males	females
PC ae C44:6	β_0	0.283	0.282	0.299
	P_0	0.000258	0.00596	0.0133
	bias (β_0)	0.00486	0.00453	0.0105
	β_{boot}	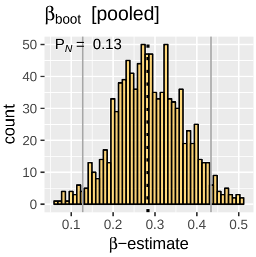	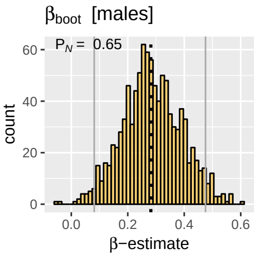	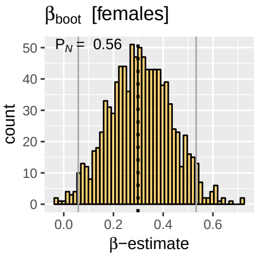
	$\mu(\beta)_{boot}$	0.288	0.286	0.309
	$\mu(SE)_{boot}$	0.0783	0.104	0.123
	P_{boot}	0.000233	0.006	0.012
	% $P_{bonf.sign.}$	63.9	27.5	20
	% $P_{nom.sign.}$	95.6	79.4	71

	P_{boot}	% $P_{nom.sign.}$	I^2_{boot} (% I^2_{boot} = 0)
Δ Sex	0.891	4.8	0 (68)

metabolite	statistic	pooled	males	females
PC ae C44:4	β_0	0.265	0.274	0.255
	P_0	0.000457	0.00629	0.0307
	bias (β_0)	0.00664	0.00383	0.0136
	β_{boot}	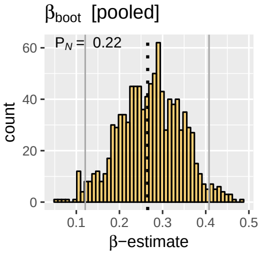	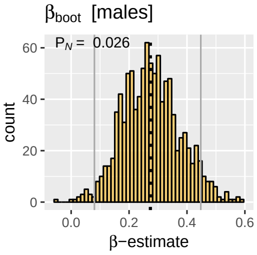	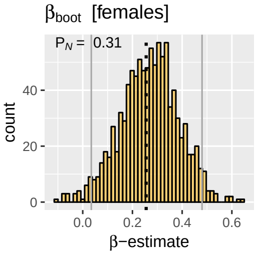
	$\mu(\beta)_{boot}$	0.272	0.277	0.269
	$\mu(SE)_{boot}$	0.0765	0.102	0.121
	P_{boot}	0.000378	0.00638	0.0259
	% $P_{bonf.sign.}$	60.8	25	11.2
	% $P_{nom.sign.}$	94.8	78.6	62.3

	P_{boot}	% $P_{nom.sign.}$	I^2_{boot} (% I^2_{boot} = 0)
Δ Sex	0.955	4.7	0 (70)

metabolite	statistic	pooled	males	females
PC ae C44:5	β_0	0.26	0.294	0.214
	P_0	0.000523	0.00326	0.0638
	bias (β_0)	0.00113	0.00458	0.000925
	β_{boot}	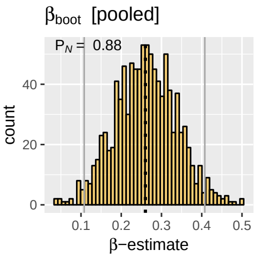	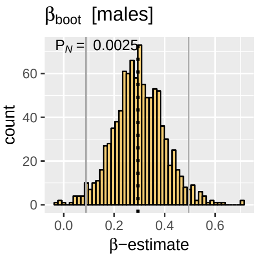	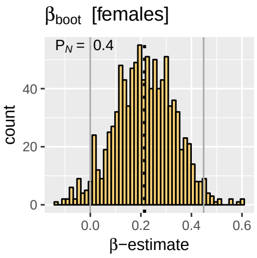
	$\mu(\beta)_{boot}$	0.261	0.299	0.215
	$\mu(SE)_{boot}$	0.0758	0.102	0.118
	P_{boot}	0.000567	0.00337	0.0672
	% $P_{bonf.sign.}$	55.4	34.4	5
	% $P_{nom.sign.}$	93.4	85.6	46.5
	Δ Sex	P_{boot}	% $P_{nom.sign.}$	I^2_{boot} (% I^2_{boot} = 0)
		0.591	8.8	0 (64)

metabolite	statistic	pooled	males	females
Threonine	β_0	0.207	0.372	0.0699
	P_0	0.00672	0.000883	0.517
	bias (β_0)	0.00311	0.00544	0.00391
	β_{boot}	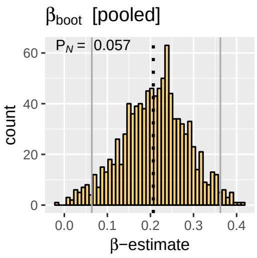	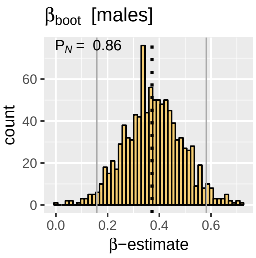	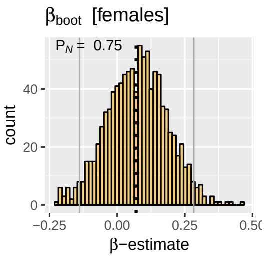
	$\mu(\beta)_{boot}$	0.21	0.377	0.0738
	$\mu(SE)_{boot}$	0.077	0.114	0.11
	P_{boot}	0.00642	0.000892	0.502
	% $P_{bonf.sign.}$	27.1	50.9	0.3
	% $P_{nom.sign.}$	79.1	92.2	9.6
	Δ Sex	P_{boot}	% $P_{nom.sign.}$	I^2_{boot} (% I^2_{boot} = 0)
		0.0553	47.8	47.8 (19)

metabolite	statistic	pooled	males	females
Valine	β_0	-0.134	0.0319	-0.299
	P_0	0.105	0.78	0.015
	bias (β_0)	-0.00348	-0.00022	-0.00717
	β_{boot}	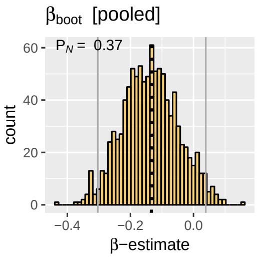	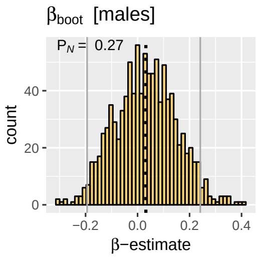	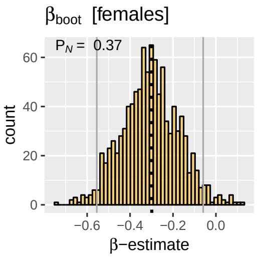
	$\mu(\beta)_{boot}$	-0.137	0.0317	-0.306
	$\mu(SE)_{boot}$	0.0833	0.116	0.125
	P_{boot}	0.0995	0.785	0.0144
	% $P_{bonf.sign.}$	4.3	0	19.9
	% $P_{nom.sign.}$	39.8	5	70.3
	Δ Sex	P_{boot}	% $P_{nom.sign.}$	I^2_{boot} (% I^2_{boot} = 0)
		0.0483	49.9	49.4 (18)

CSF p-tau

metabolite	statistic	pooled	males	females
C10	β_0	0.0839	0.0141	0.144
	P_0	0.00458	0.734	0.000607
	bias (β_0)	0.000206	-0.00031	0.000518
	β_{boot}	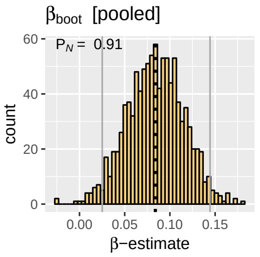	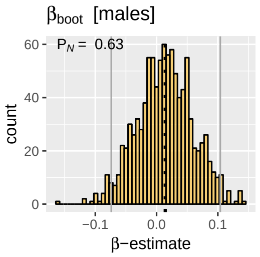	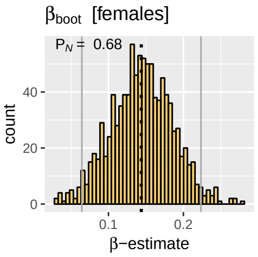
	$\mu(\beta)_{boot}$	0.0841	0.0138	0.144
	$\mu(SE)_{boot}$	0.0295	0.0416	0.0417
	P_{boot}	0.00438	0.74	0.000545
	% $P_{bonf.sign.}$	33.7	0.2	54.8
	% $P_{nom.sign.}$	80.1	8.9	92.5
	Δ Sex	P_{boot}	% $P_{nom.sign.}$	I^2_{boot} (% I^2_{boot} = 0)
		0.0267	59.6	54.9 (12)

metabolite	statistic	pooled	males	females
C5-DC (C6-OH)	β_0	0.103	0.0116	0.205
	P_0	0.0235	0.852	0.00227
	bias (β_0)	-0.000864	0.00161	-0.00285
	β_{boot}	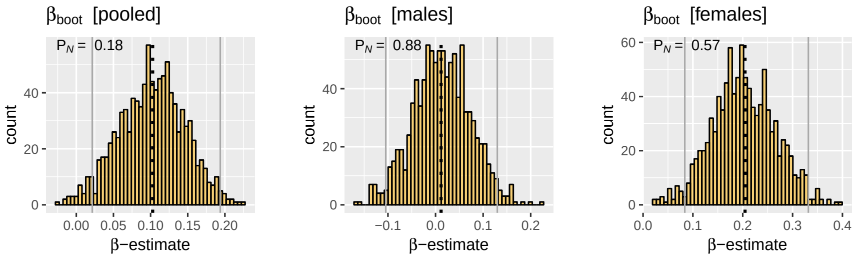		
	$\mu(\beta)_{boot}$	0.102	0.0132	0.202
	$\mu(SE)_{boot}$	0.0454	0.0626	0.0668
	P_{boot}	0.0248	0.832	0.00253
	% $P_{bonf.sign.}$	13.4	0.1	36.6
	% $P_{nom.sign.}$	63.1	4.7	86.3

Δ Sex	P_{boot}	% $P_{nom.sign.}$	I^2_{boot} (% I^2_{boot} = 0)
	0.0392	53.9	51.5 (15)

metabolite	statistic	pooled	males	females
C8	β_0	0.0644	0.00316	0.127
	P_0	0.0342	0.939	0.00511
	bias (β_0)	-0.000902	-0.000382	-0.00103
	β_{boot}	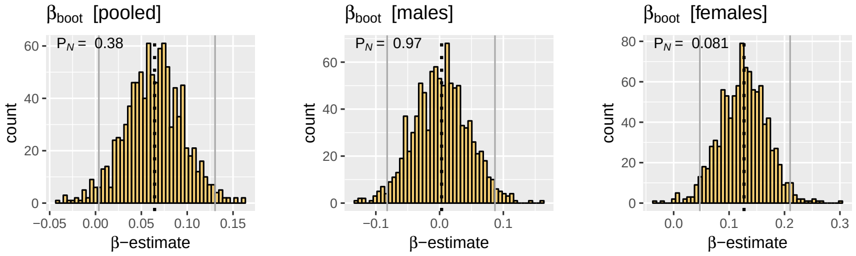		
	$\mu(\beta)_{boot}$	0.0635	0.00278	0.126
	$\mu(SE)_{boot}$	0.0305	0.0412	0.0453
	P_{boot}	0.037	0.946	0.00548
	% $P_{bonf.sign.}$	10.8	0.2	27.6
	% $P_{nom.sign.}$	55.7	5.6	81.1

Δ Sex	P_{boot}	% $P_{nom.sign.}$	I^2_{boot} (% I^2_{boot} = 0)
	0.0441	53.1	50.3 (14)

metabolite	statistic	pooled	males	females
	β_0	0.0557	0.129	-0.0228
	P_0	0.0865	0.0048	0.618
	bias (β_0)	0.000661	0.00102	0.000452
	β_{boot}	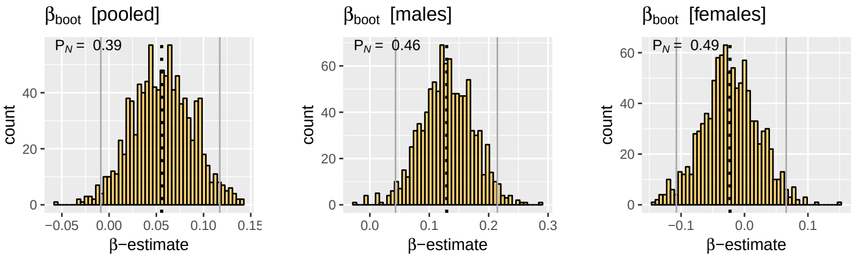		
	$\mu(\beta)_{boot}$	0.0563	0.13	-0.0223
PC ae C36:2	$\mu(SE)_{boot}$	0.0325	0.0458	0.0457
	P_{boot}	0.0828	0.00439	0.625
	% $P_{bonf.sign.}$	5	31.4	0
	% $P_{nom.sign.}$	40.5	82.4	6.2

	Δ Sex	P_{boot}	% $P_{nom.sign.}$	I^2_{boot} (% I^2_{boot} = 0)
		0.0182	68.2	57.6 (7.9)

metabolite	statistic	pooled	males	females
	β_0	-0.0338	0.0325	-0.105
	P_0	0.272	0.439	0.0197
	bias (β_0)	0.000196	-0.00113	0.00152
	β_{boot}	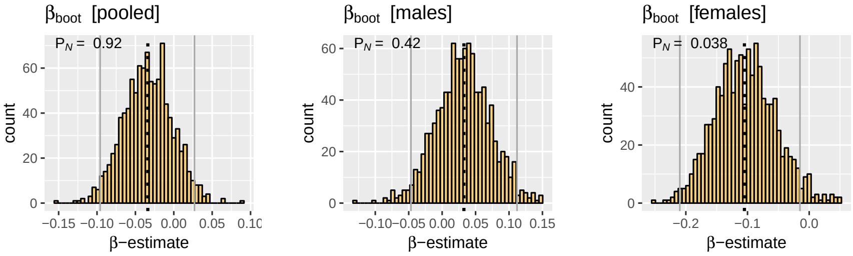		
	$\mu(\beta)_{boot}$	-0.0336	0.0314	-0.103
Histidine	$\mu(SE)_{boot}$	0.0308	0.0422	0.0449
	P_{boot}	0.276	0.456	0.0212
	% $P_{bonf.sign.}$	1.7	0.1	17.8
	% $P_{nom.sign.}$	20.4	10.2	63.1

	Δ Sex	P_{boot}	% $P_{nom.sign.}$	I^2_{boot} (% I^2_{boot} = 0)
		0.0285	60.1	54.3 (11)

metabolite	statistic	pooled	males	females
Asparagine	β_0	0.0336	0.107	-0.0524
	P_0	0.284	0.0166	0.232
	bias (β_0)	-0.000114	-0.000374	-9.01E-05
	β_{boot}	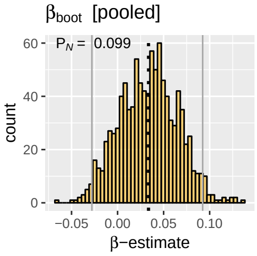	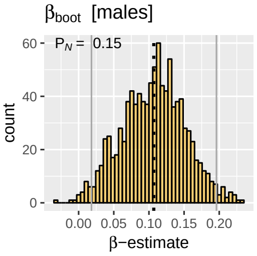	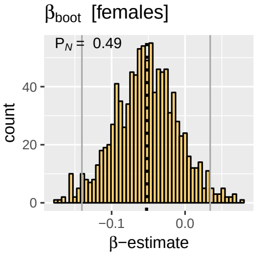
	$\mu(\beta)_{boot}$	0.0335	0.107	-0.0525
	$\mu(SE)_{boot}$	0.0314	0.0446	0.0439
	P_{boot}	0.286	0.017	0.231
	% $P_{bonf.sign.}$	1.1	17	1.5
	% $P_{nom.sign.}$	19.7	66.2	21.5
	Δ Sex	P_{boot}	% $P_{nom.sign.}$	$I^2{}_{boot}$ (% $I^2{}_{boot}$ = 0)
		0.011	71.5	60.7 (5.1)

metabolite	statistic	pooled	males	females
SM (OH) C16:1	β_0	0.032	0.0909	-0.0389
	P_0	0.31	0.0336	0.399
	bias (β_0)	0.000366	0.00102	-0.000497
	β_{boot}	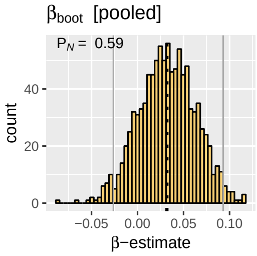	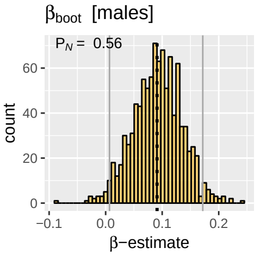	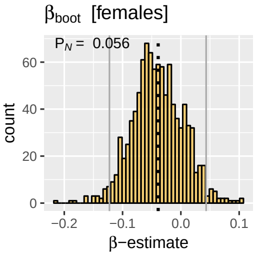
	$\mu(\beta)_{boot}$	0.0323	0.092	-0.0394
	$\mu(SE)_{boot}$	0.0315	0.0428	0.0461
	P_{boot}	0.304	0.0315	0.393
	% $P_{bonf.sign.}$	0.7	11.7	0.8
	% $P_{nom.sign.}$	18.4	59.2	11
	Δ Sex	P_{boot}	% $P_{nom.sign.}$	$I^2{}_{boot}$ (% $I^2{}_{boot}$ = 0)
		0.0365	57.2	52.2 (14)

metabolite	statistic	pooled	males	females
Glycine	β_0	0.0296	0.104	-0.0256
	P_0	0.35	0.0394	0.523
	bias (β_0)	-0.00114	-0.0019	-0.0013
	β_{boot}	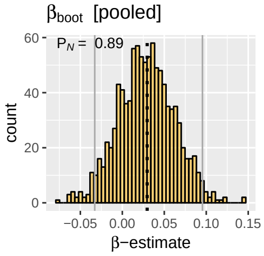	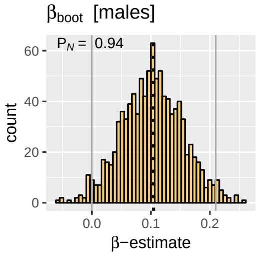	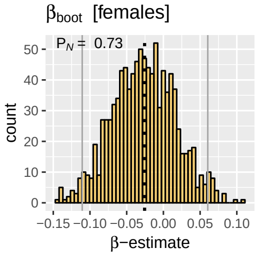
	$\mu(\beta)_{boot}$	0.0285	0.102	-0.0269
	$\mu(SE)_{boot}$	0.0317	0.0506	0.0402
	P_{boot}	0.369	0.0428	0.503
	% $P_{bonf.sign.}$	1	10.5	0.7
	% $P_{nom.sign.}$	16.5	52.7	12.2
	Δ Sex	P_{boot}	% $P_{nom.sign.}$	$I^2{}_{boot}$ (% $I^2{}_{boot}$ = 0)
		0.0447	50.6	50.2 (18)

metabolite	statistic	pooled	males	females
PC ae C36:1	β_0	0.028	0.0883	-0.0413
	P_0	0.368	0.0417	0.351
	bias (β_0)	0.000567	0.00219	-0.00172
	β_{boot}	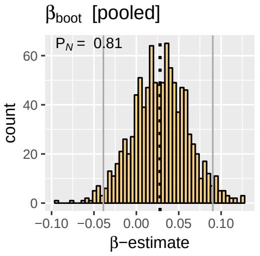	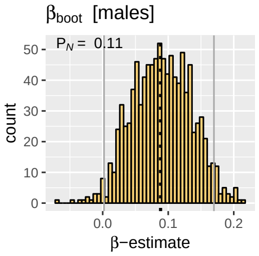	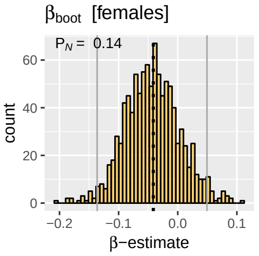
	$\mu(\beta)_{boot}$	0.0285	0.0905	-0.043
	$\mu(SE)_{boot}$	0.0311	0.0434	0.0444
	P_{boot}	0.359	0.0369	0.333
	% $P_{bonf.sign.}$	1.4	12.2	1.2
	% $P_{nom.sign.}$	15.4	54.3	16.8
	Δ Sex	P_{boot}	% $P_{nom.sign.}$	$I^2{}_{boot}$ (% $I^2{}_{boot}$ = 0)
		0.0314	58.3	53.5 (13)

metabolite	statistic	pooled	males	females
	β_0	0.0155	-0.054	0.0885
	P_0	0.585	0.167	0.0302
	bias (β_0)	0.00164	0.00196	0.00204
	β_{boot}	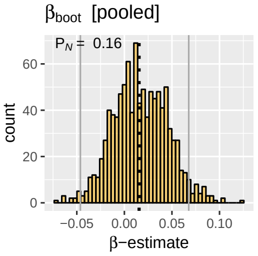	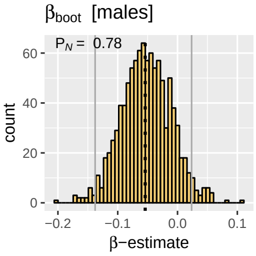	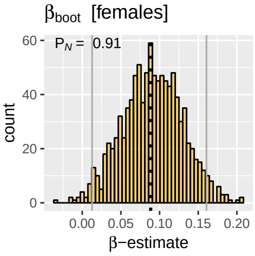
	$\mu(\beta)_{boot}$	0.0171	-0.0521	0.0906
C2	$\mu(SE)_{boot}$	0.0284	0.0391	0.0408
	P_{boot}	0.546	0.183	0.0265
	% $P_{bonf.sign.}$	0.5	2.6	11.4
	% $P_{nom.sign.}$	9	28	60.7
	Δ Sex	P_{boot}	% $P_{nom.sign.}$	I^2_{boot} (% I^2_{boot} = 0)
		0.0116	70.6	60.4 (5.4)

FDG-PET

metabolite	statistic	pooled	males	females
	β_0	-0.127	-0.14	-0.11
	P_0	2.32E-05	0.000631	0.015
	bias (β_0)	-0.00013	0.000483	-0.00101
	β_{boot}	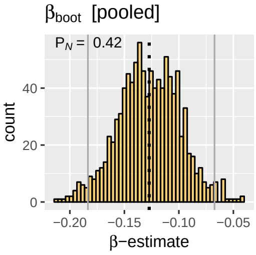	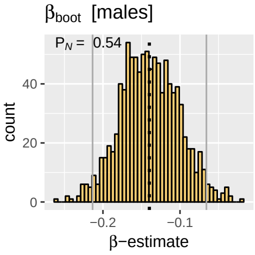	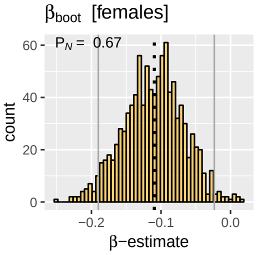
	$\mu(\beta)_{boot}$	-0.127	-0.139	-0.111
PC aa C32:1	$\mu(SE)_{boot}$	0.0299	0.0407	0.0449
	P_{boot}	2.08E-05	0.000621	0.0138
	% $P_{bonf.sign.}$	83.5	54.9	17.9
	% $P_{nom.sign.}$	99.2	93.8	69.6
	Δ Sex	P_{boot}	% $P_{nom.sign.}$	I^2_{boot} (% I^2_{boot} = 0)
		0.636	5.8	0 (63)

metabolite	statistic	pooled	males	females
	β_0	-0.111	-0.0974	-0.141
	P_0	0.000227	0.018	0.00184
	bias (β_0)	-0.00105	-0.00203	-0.000168
	β_{boot}	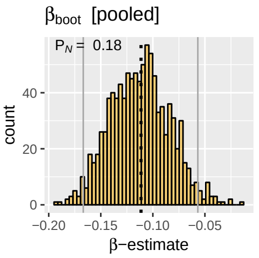	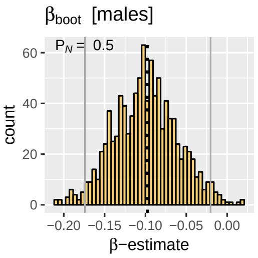	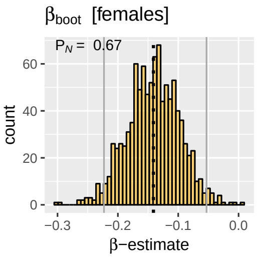
PC ae C44:4	$\mu(\beta)_{boot}$	-0.112	-0.0994	-0.141
	$\mu(SE)_{boot}$	0.0301	0.0411	0.0451
	P_{boot}	0.000189	0.0155	0.00174
	% $P_{bonf.sign.}$	67.5	17.6	41.6
	% $P_{nom.sign.}$	96.8	69.9	89.3

	P_{boot}	% $P_{nom.sign.}$	I^2_{boot} (% $I^2_{boot} = 0$)
Δ Sex	0.492	10	0 (61)

metabolite	statistic	pooled	males	females
	β_0	-0.105	-0.112	-0.111
	P_0	0.000407	0.0058	0.013
	bias (β_0)	-0.0012	-0.00169	-0.000339
	β_{boot}	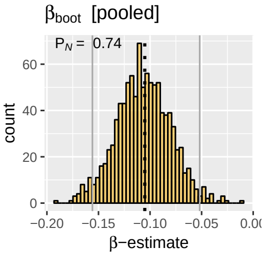	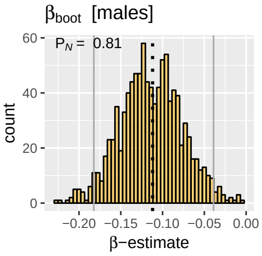	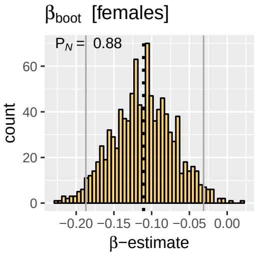
PC ae C44:5	$\mu(\beta)_{boot}$	-0.106	-0.114	-0.111
	$\mu(SE)_{boot}$	0.0296	0.0405	0.0444
	P_{boot}	0.000335	0.005	0.0125
	% $P_{bonf.sign.}$	62.3	29	19.1
	% $P_{nom.sign.}$	96.8	82.4	71.7

	P_{boot}	% $P_{nom.sign.}$	I^2_{boot} (% $I^2_{boot} = 0$)
Δ Sex	0.967	2.7	0 (71)

metabolite	statistic	pooled	males	females
PC aa C32:0	β_0	-0.107	-0.125	-0.0906
	P_0	0.000685	0.00567	0.0425
	bias (β_0)	-0.00132	0.000661	-0.00381

β_{boot}

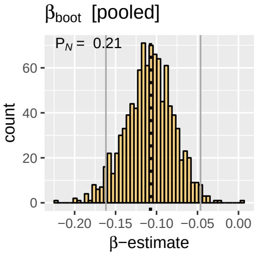

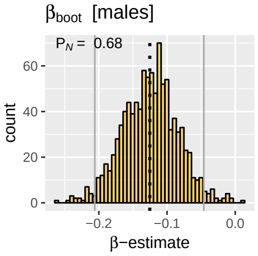

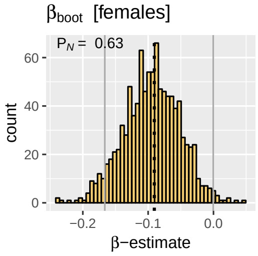

metabolite	statistic	pooled	males	females
PC aa C32:0	$\mu(\beta)_{boot}$	-0.109	-0.125	-0.0944
	$\mu(SE)_{boot}$	0.0315	0.0451	0.0446
	P_{boot}	0.000563	0.00577	0.0342
	% $P_{bonf.sign.}$	54.6	26.1	10.3
	% $P_{nom.sign.}$	94.8	82	56
	Δ Sex	P_{boot}	% $P_{nom.sign.}$	$I^2{}_{boot}$ (% $I^2{}_{boot}$ = 0)
		0.635	5.2	0 (65)

metabolite	statistic	pooled	males	females
PC ae C42:4	β_0	-0.103	-0.103	-0.112
	P_0	0.000856	0.0158	0.0133
	bias (β_0)	-0.00249	-0.00367	-0.00161

β_{boot}

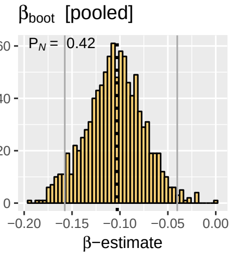

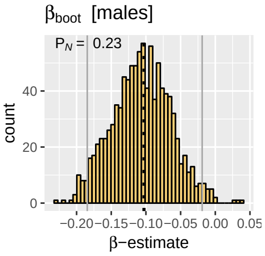

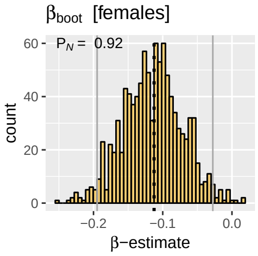

metabolite	statistic	pooled	males	females
PC ae C42:4	$\mu(\beta)_{boot}$	-0.105	-0.106	-0.114
	$\mu(SE)_{boot}$	0.0308	0.0425	0.0454
	P_{boot}	0.000623	0.0122	0.012
	% $P_{bonf.sign.}$	53.2	20.5	19.2
	% $P_{nom.sign.}$	93.4	69.3	72.4
	Δ Sex	P_{boot}	% $P_{nom.sign.}$	$I^2{}_{boot}$ (% $I^2{}_{boot}$ = 0)
		0.902	4.5	0 (69)

metabolite	statistic	pooled	males	females
C16:1	β_0	-0.103	-0.165	-0.0285
	P_0	0.000909	9.64E-05	0.538
	bias (β_0)	-2.19E-05	-0.000244	0.000573
	β_{boot}	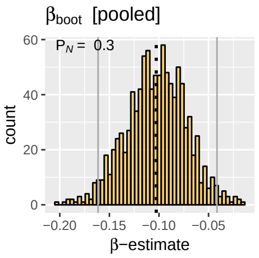	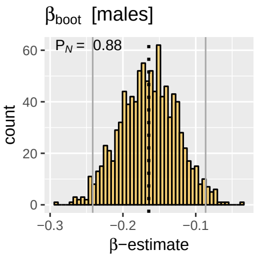	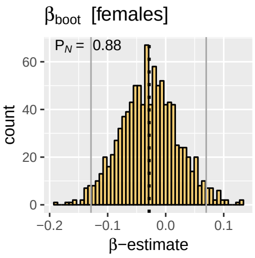
	$\mu(\beta)_{boot}$	-0.103	-0.165	-0.0279
	$\mu(SE)_{boot}$	0.0309	0.0419	0.0463
	P_{boot}	0.000879	8.44E-05	0.546
	% $P_{bonf.sign.}$	49.9	72.4	0.3
	% $P_{nom.sign.}$	92.6	98.2	11.6
	Δ Sex	P_{boot}	% $P_{nom.sign.}$	I^2_{boot} (% I^2_{boot} = 0)
		0.0284	57.5	54.4 (14)

metabolite	statistic	pooled	males	females
PC ae C40:2	β_0	-0.0529	-0.119	0.016
	P_0	0.0782	0.00434	0.715
	bias (β_0)	0.000617	0.00174	-0.000552
	β_{boot}	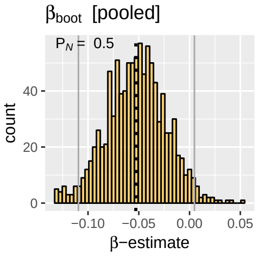	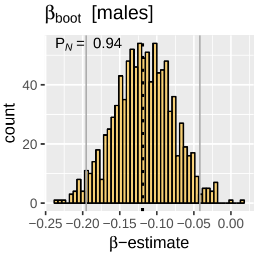	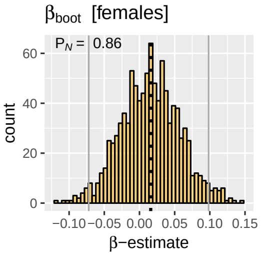
	$\mu(\beta)_{boot}$	-0.0523	-0.117	0.0154
	$\mu(SE)_{boot}$	0.0301	0.0417	0.0438
	P_{boot}	0.0818	0.00485	0.725
	% $P_{bonf.sign.}$	5	29	0
	% $P_{nom.sign.}$	40.1	82	5.8
	Δ Sex	P_{boot}	% $P_{nom.sign.}$	I^2_{boot} (% I^2_{boot} = 0)
		0.0279	58.5	54.5 (9.6)

metabolite	statistic	pooled	males	females
	β_0	-0.0234	0.0591	-0.118
	P_0	0.451	0.177	0.00818
	bias (β_0)	-0.00143	-0.00103	-0.00162
	β_{boot}	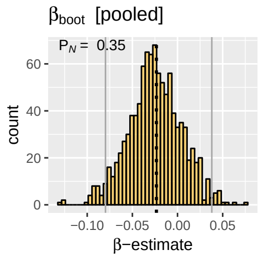	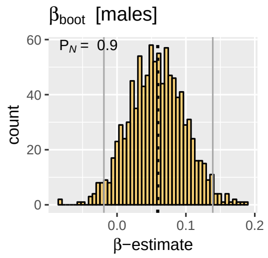	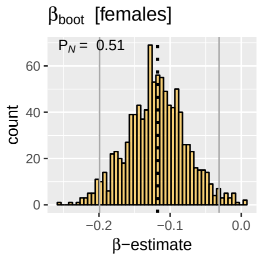
Proline	$\mu(\beta)_{boot}$	-0.0248	0.0581	-0.119
	$\mu(SE)_{boot}$	0.031	0.0437	0.0443
	P_{boot}	0.424	0.184	0.00709
	% $P_{bonf.sign.}$	0.3	1.7	24.9
	% $P_{nom.sign.}$	10.7	25.5	78.9
	Δ Sex	P_{boot}	% $P_{nom.sign.}$	I^2_{boot} (% I^2_{boot} = 0)
		0.00432	80.4	65 (1.9)

APOE ε4 status-stratified analyses

Pathological CSF A$\beta_{1\text{-}42}$

metabolite	statistic	pooled	*APOE* ε4+	*APOE* ε4-
	β_0	0.283	0.63	0.158
	P_0	0.000258	2.5E-05	0.0796
	bias (β_0)	0.00486	0.00984	0.00611
	β_{boot}	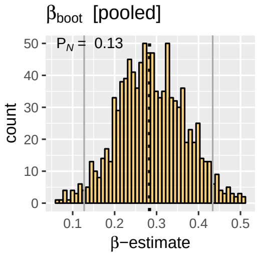	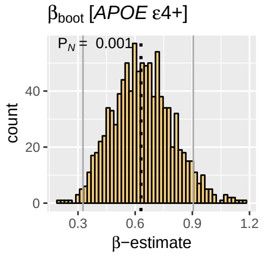	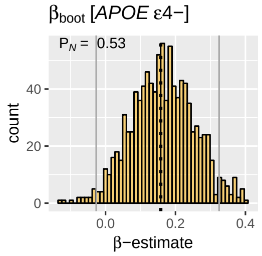
PC ae C44:6	$\mu(\beta)_{boot}$	0.288	0.64	0.164
	$\mu(SE)_{boot}$	0.0783	0.153	0.0914
	P_{boot}	0.000233	2.85E-05	0.0724
	% $P_{bonf.sign.}$	63.9	81.4	5
	% $P_{nom.sign.}$	95.6	99.6	44
	Δ *APOE* $\varepsilon4\pm$	P_{boot}	% $P_{nom.sign.}$	I^2_{boot} (% I^2_{boot} = 0)
		0.00791	76.1	62.3 (4)

metabolite	statistic	pooled	*APOE* ε4+	*APOE* ε4-
	β_0	0.265	0.565	0.139
	P_0	0.000457	0.00013	0.113
	bias (β_0)	0.00664	0.011	0.00656
	β_{boot}	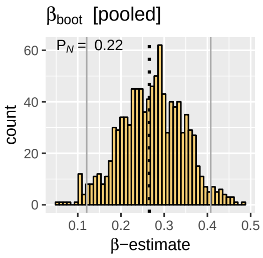	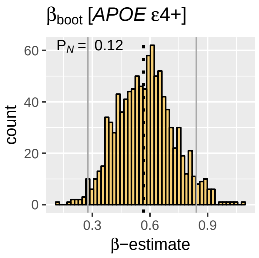	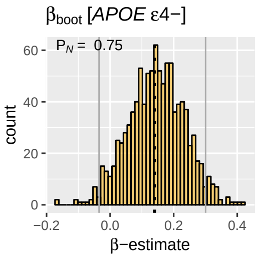
	$\mu(\beta)_{boot}$	0.272	0.577	0.146
PC ae C44:4	$\mu(SE)_{boot}$	0.0765	0.151	0.0893
	P_{boot}	0.000378	0.000137	0.102
	% $P_{bonf.sign.}$	60.8	69.9	3.4
	% $P_{nom.sign.}$	94.8	98.1	37.3
	Δ *APOE* ε4±	P_{boot}	% $P_{nom.sign.}$	I^2_{boot} (% I^2_{boot} = 0)
		0.0148	67.2	59 (6.1)

metabolite	statistic	pooled	*APOE* ε4+	*APOE* ε4-
	β_0	0.26	0.609	0.129
	P_0	0.000523	2.64E-05	0.137
	bias (β_0)	0.00113	0.00849	0.000964
	β_{boot}	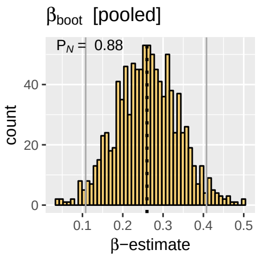	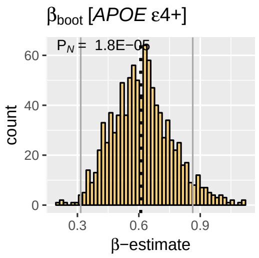	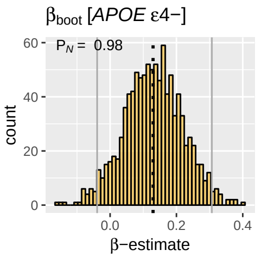
	$\mu(\beta)_{boot}$	0.261	0.618	0.13
PC ae C44:5	$\mu(SE)_{boot}$	0.0758	0.148	0.0884
	P_{boot}	0.000567	3.18E-05	0.14
	% $P_{bonf.sign.}$	55.4	82	2.9
	% $P_{nom.sign.}$	93.4	99.5	31.1
	Δ *APOE* ε4±	P_{boot}	% $P_{nom.sign.}$	I^2_{boot} (% I^2_{boot} = 0)
		0.00504	81.7	64.3 (1.9)

metabolite	statistic	pooled	*APOE* ε4+	*APOE* ε4-
PC ae C42:4	β_0	0.242	0.564	0.114
	P_0	0.00198	0.000132	0.215
	bias (β_0)	0.000364	0.00878	-0.00137

β_{boot}

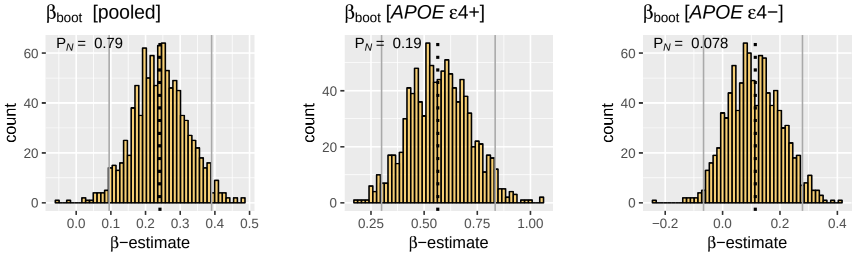

metabolite	statistic	pooled	*APOE* ε4+	*APOE* ε4-
PC ae C42:4	$\mu(\beta)_{boot}$	0.242	0.573	0.113
	$\mu(SE)_{boot}$	0.0788	0.151	0.0931
	P_{boot}	0.00214	0.000147	0.227
	% $P_{bonf.sign.}$	38.5	70	1.6
	% $P_{nom.sign.}$	88.3	98.1	22.8
	Δ *APOE* $\varepsilon 4\pm$	P_{boot}	% $P_{nom.sign.}$	I^2_{boot} (% I^2_{boot} = 0)
		0.00975	74.9	61.3 (3.9)

metabolite	statistic	pooled	*APOE* ε4+	*APOE* ε4-
Proline	β_0	-0.0755	0.176	-0.202
	P_0	0.352	0.215	0.044
	bias (β_0)	-0.0019	-0.00185	-0.00142

β_{boot}

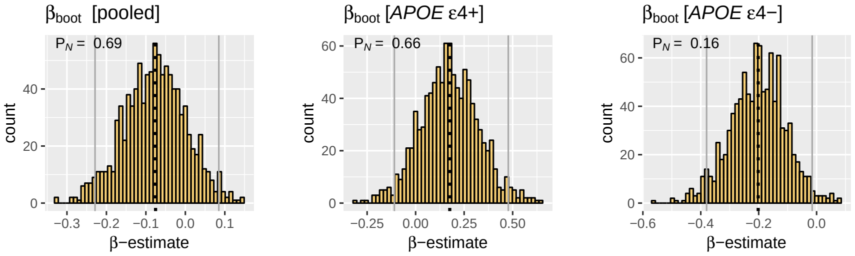

metabolite	statistic	pooled	*APOE* ε4+	*APOE* ε4-
Proline	$\mu(\beta)_{boot}$	-0.0774	0.174	-0.203
	$\mu(SE)_{boot}$	0.0819	0.145	0.102
	P_{boot}	0.345	0.231	0.0455
	% $P_{bonf.sign.}$	0.7	1.9	7.3
	% $P_{nom.sign.}$	14.7	21.9	51.1
	Δ *APOE* $\varepsilon 4\pm$	P_{boot}	% $P_{nom.sign.}$	I^2_{boot} (% I^2_{boot} = 0)
		0.0341	57.3	52.8 (12)

metabolite	statistic	pooled	*APOE* ε4+	*APOE* ε4-
	β_0	0.0603	0.363	-0.102
	P_0	0.46	0.0183	0.305
	bias (β_0)	0.000846	0.0135	-0.00342
	β_{boot}	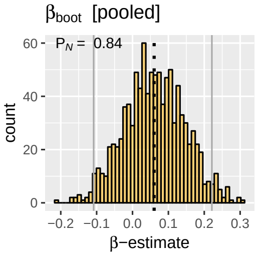	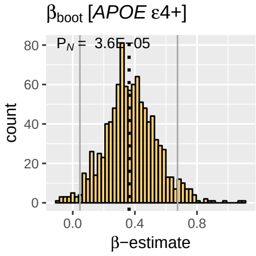	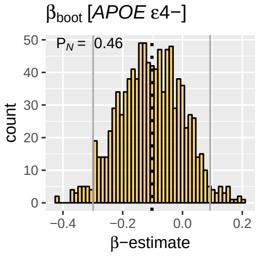
Glycine	$\mu(\beta)_{boot}$	0.0612	0.376	-0.106
	$\mu(SE)_{boot}$	0.0823	0.157	0.101
	P_{boot}	0.457	0.0164	0.296
	% $P_{bonf.sign.}$	0.3	15.7	1.1
	% $P_{nom.sign.}$	12.5	69.2	20.4
	Δ *APOE* ε4±	P_{boot}	% $P_{nom.sign.}$	I^2_{boot} (% I^2_{boot} = 0)
		0.0104	72.8	61 (6.8)

FDG-PET

metabolite	statistic	pooled	*APOE* ε4+	*APOE* ε4-
	β_0	-0.127	-0.0873	-0.162
	P_0	2.32E-05	0.0535	0.000134
	bias (β_0)	-0.00013	-0.00142	0.000826
	β_{boot}	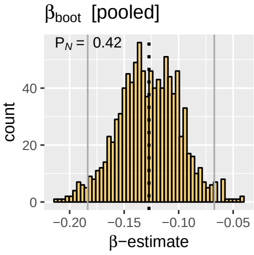	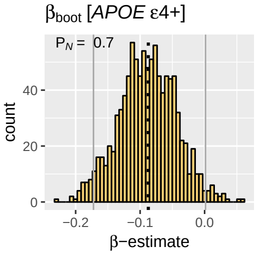	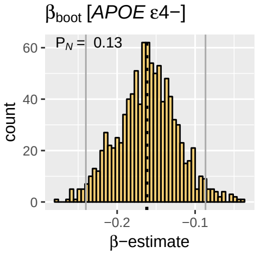
PC aa C32:1	$\mu(\beta)_{boot}$	-0.127	-0.0887	-0.161
	$\mu(SE)_{boot}$	0.0299	0.0452	0.0421
	P_{boot}	2.08E-05	0.0495	0.000131
	% $P_{bonf.sign.}$	83.5	9.5	70.7
	% $P_{nom.sign.}$	99.2	50.3	97.5
	Δ *APOE* ε4±	P_{boot}	% $P_{nom.sign.}$	I^2_{boot} (% I^2_{boot} = 0)
		0.241	19.2	14.8 (42)

metabolite	statistic	pooled	*APOE* ε4+	*APOE* ε4-
PC ae C44:4	β_0	-0.111	-0.115	-0.114
	P_0	0.000227	0.0139	0.00634
	bias (β_0)	-0.00105	-0.000257	-0.00172
	β_{boot}	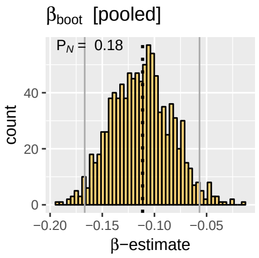	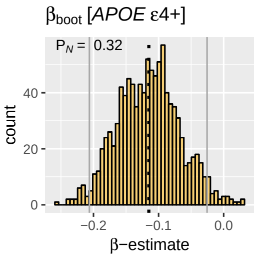	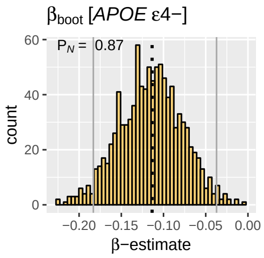
	$\mu(\beta)_{boot}$	-0.112	-0.115	-0.115
	$\mu(SE)_{boot}$	0.0301	0.0466	0.0415
	P_{boot}	0.000189	0.0134	0.00549
	% $P_{bonf.sign.}$	67.5	20.6	26.3
	% $P_{nom.sign.}$	96.8	70.1	81

Δ *APOE* ε4±	P_{boot}	% $P_{nom.sign.}$	I^2_{boot} (% I^2_{boot} = 0)
	1	4.6	0 (70)

metabolite	statistic	pooled	*APOE* ε4+	*APOE* ε4-
PC ae C44:5	β_0	-0.105	-0.122	-0.102
	P_0	0.000407	0.00834	0.013
	bias (β_0)	-0.0012	-0.00222	-0.000556
	β_{boot}	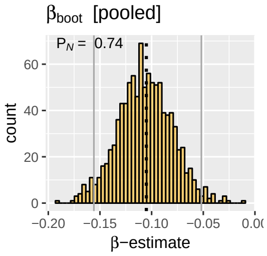	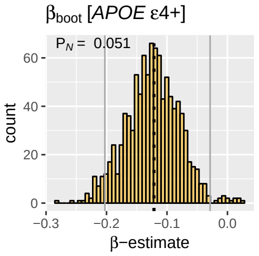	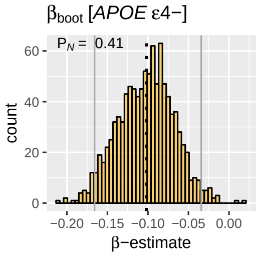
	$\mu(\beta)_{boot}$	-0.106	-0.124	-0.102
	$\mu(SE)_{boot}$	0.0296	0.046	0.0408
	P_{boot}	0.000335	0.00712	0.0123
	% $P_{bonf.sign.}$	62.3	24.1	17.1
	% $P_{nom.sign.}$	96.8	76.8	73.6

Δ *APOE* ε4±	P_{boot}	% $P_{nom.sign.}$	I^2_{boot} (% I^2_{boot} = 0)
	0.724	4.4	0 (69)

metabolite	statistic	pooled	*APOE ε4+*	*APOE ε4-*
PC aa C32:0	β_0	-0.107	-0.135	-0.0823
	P_0	0.000685	0.00439	0.0658
	bias (β_0)	-0.00132	-0.00386	0.000352
	β_{boot}	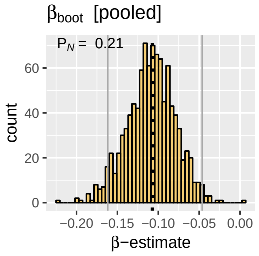	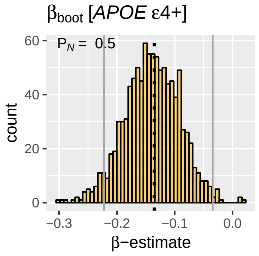	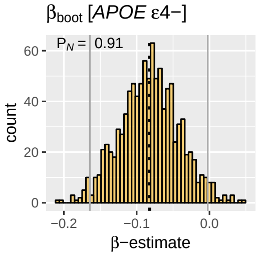
	$\mu(\beta)_{boot}$	-0.109	-0.139	-0.0819
	$\mu(SE)_{boot}$	0.0315	0.0474	0.0446
	P_{boot}	0.000563	0.0033	0.0664
	% $P_{bonf.sign.}$	54.6	34.2	5.3
	% $P_{nom.sign.}$	94.8	83.7	43.6
	Δ *APOE ε4±*	P_{boot}	% $P_{nom.sign.}$	$I^2{}_{boot}$ (% $I^2{}_{boot}$ = 0)
		0.378	14.1	0 (53)

metabolite	statistic	pooled	*APOE ε4+*	*APOE ε4-*
PC ae C42:4	β_0	-0.103	-0.131	-0.0859
	P_0	0.000856	0.00579	0.0451
	bias (β_0)	-0.00249	-0.00038	-0.00426
	β_{boot}	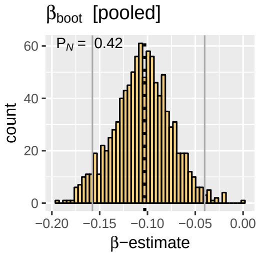	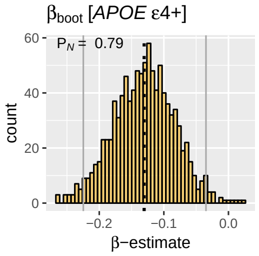	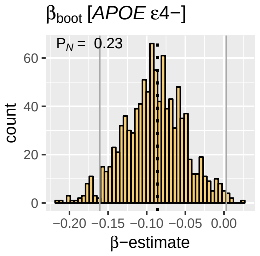
	$\mu(\beta)_{boot}$	-0.105	-0.131	-0.0901
	$\mu(SE)_{boot}$	0.0308	0.0472	0.0429
	P_{boot}	0.000623	0.00553	0.0355
	% $P_{bonf.sign.}$	53.2	29.4	9.1
	% $P_{nom.sign.}$	93.4	78.3	56.6
	Δ *APOE ε4±*	P_{boot}	% $P_{nom.sign.}$	$I^2{}_{boot}$ (% $I^2{}_{boot}$ = 0)
		0.522	9.5	0 (60)

metabolite	statistic	pooled	*APOE* ε4+	*APOE* ε4-
C10	β_0	-0.0573	0.0373	-0.135
	P_0	0.0514	0.417	0.000717
	bias (β_0)	-0.0007	-0.000221	-0.000571
	β_{boot}	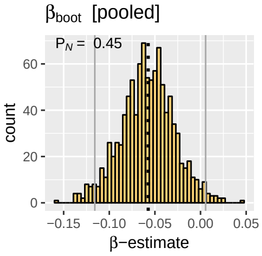	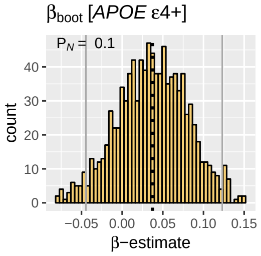	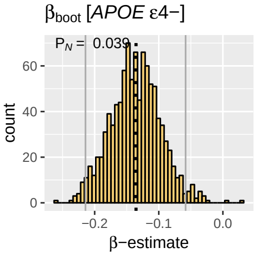
	$\mu(\beta)_{boot}$	-0.058	0.0371	-0.136
	$\mu(SE)_{boot}$	0.0294	0.0461	0.0399
	P_{boot}	0.0486	0.421	0.000655
	% $P_{bonf.sign.}$	9.2	0	53.5
	% $P_{nom.sign.}$	50.7	10.1	93.4
	Δ *APOE* ε4±	P_{boot}	% $P_{nom.sign.}$	I^2_{boot} (% I^2_{boot} = 0)
		0.00447	83.1	64.8 (3.3)

metabolite	statistic	pooled	*APOE* ε4+	*APOE* ε4-
C8	β_0	-0.0505	0.0381	-0.138
	P_0	0.0996	0.404	0.00158
	bias (β_0)	-0.00134	-0.00293	7.96E-05
	β_{boot}	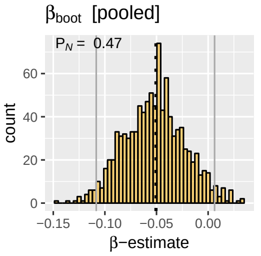	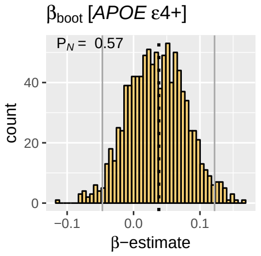	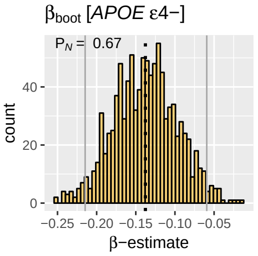
	$\mu(\beta)_{boot}$	-0.0519	0.0351	-0.138
	$\mu(SE)_{boot}$	0.0306	0.0456	0.0435
	P_{boot}	0.0907	0.441	0.00154
	% $P_{bonf.sign.}$	3.9	0.3	44.5
	% $P_{nom.sign.}$	38.7	11.1	89.2
	Δ *APOE* ε4±	P_{boot}	% $P_{nom.sign.}$	I^2_{boot} (% I^2_{boot} = 0)
		0.00602	79.1	63.6 (3.7)

metabolite	statistic	pooled	*APOE* ε4+	*APOE* ε4-
Valine	β_0	0.0364	-0.0397	0.106
	P_0	0.249	0.408	0.0168
	bias (β_0)	-0.000386	-0.00077	8.57E-05
	β_{boot}	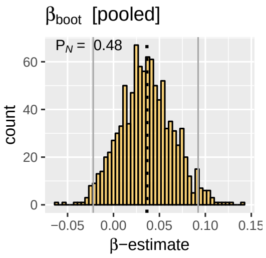	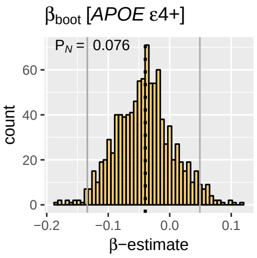	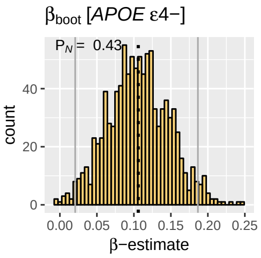
	$\mu(\beta)_{boot}$	0.036	-0.0405	0.106
	$\mu(SE)_{boot}$	0.0315	0.0479	0.0442
	P_{boot}	0.254	0.398	0.0165
	% $P_{bonf.sign.}$	1.1	0.5	16.5
	% $P_{nom.sign.}$	18.8	12.9	67.1
	Δ *APOE* ε4±	P_{boot}	% $P_{nom.sign.}$	$I^2{}_{boot}$ (% $I^2{}_{boot}$ = 0)
		0.0245	62.4	55.5 (10)

metabolite	statistic	pooled	*APOE* ε4+	*APOE* ε4-
Glycine	β_0	-0.0325	-0.14	0.059
	P_0	0.3	0.00305	0.18
	bias (β_0)	-0.000904	-0.00172	-0.000546
	β_{boot}	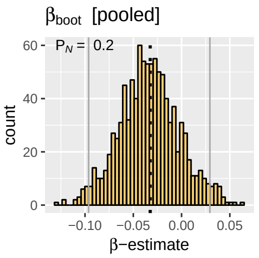	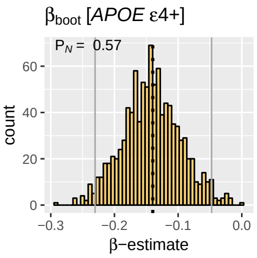	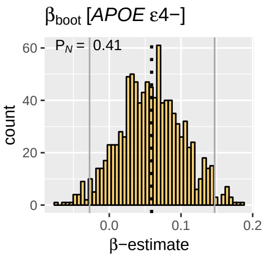
	$\mu(\beta)_{boot}$	-0.0334	-0.142	0.0585
	$\mu(SE)_{boot}$	0.0313	0.047	0.044
	P_{boot}	0.286	0.0026	0.184
	% $P_{bonf.sign.}$	1	37.3	2.6
	% $P_{nom.sign.}$	17.7	86.2	26.2
	Δ *APOE* ε4±	P_{boot}	% $P_{nom.sign.}$	$I^2{}_{boot}$ (% $I^2{}_{boot}$ = 0)
		0.00187	88.1	67.8 (1.9)

metabolite	statistic	pooled	*APOE* ε4+	*APOE* ε4-
	β_0	-0.0234	-0.1	0.0481
	P_0	0.451	0.0339	0.264
	bias (β_0)	-0.00143	-0.0019	-0.00158
	β_{boot}	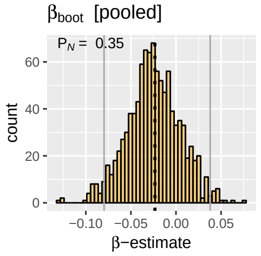	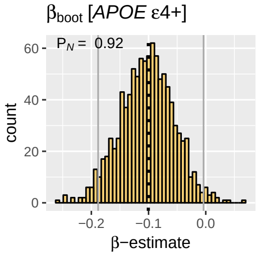	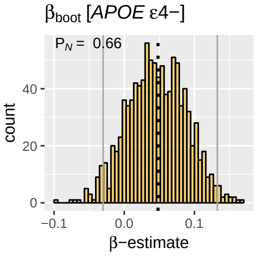
Proline	$\mu(\beta)_{boot}$	-0.0248	-0.102	0.0465
	$\mu(SE)_{boot}$	0.031	0.0473	0.0431
	P_{boot}	0.424	0.0303	0.28
	% $P_{bonf.sign.}$	0.3	12.7	0.7
	% $P_{nom.sign.}$	10.7	57.1	18.8

	Δ *APOE* ε4±	P_{boot}	% $P_{nom.sign.}$	I^2_{boot} (% I^2_{boot} = 0)
		0.0199	64	57.1 (8.4)

2-fold stratified analyses (sex * *APOE* ε4 status)

Pathological CSF A$\beta_{1\text{-}42}$

metabolite	statistic	*APOE* ε4+ males	*APOE* ε4+ females	*APOE* ε4- males	*APOE* ε4- females
	β_0	0.463	0.922	0.192	0.0998
	P_0	0.0168	0.00019	0.103	0.481
	bias (β_0)	0.0267	0.057	0.0054	-0.00329
	β_{boot}	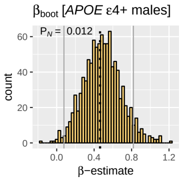	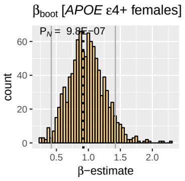	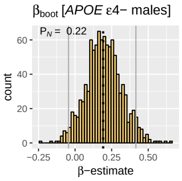	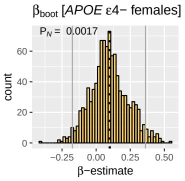
PC ae C44:6	$\mu(\beta)_{boot}$	0.49	0.979	0.197	0.0965
	$\mu(SE)_{boot}$	0.201	0.263	0.121	0.145
	P_{boot}	0.015	0.000204	0.103	0.505
	% $P_{bonf.sign.}$	16	68.8	4	0.1
	% $P_{nom.sign.}$	70	97.4	36.7	8.9

metabolite	statistic	*APOE* ε4+ males	*APOE* ε4+ females	*APOE* ε4- males	*APOE* ε4- females
	β_0	0.521	0.761	0.197	0.0223
	P_0	0.00617	0.000829	0.0879	0.87
	bias (β_0)	0.024	0.031	0.00801	-0.0084
PC ae C44:5	β_{boot}	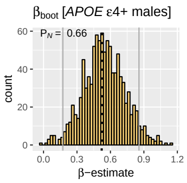	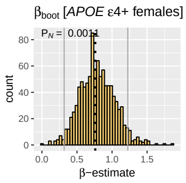	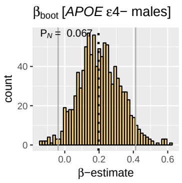	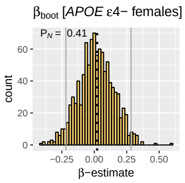
	$\mu(\beta)_{\mathrm{boot}}$	0.545	0.792	0.205	0.0139
	$\mu(\mathrm{SE})_{\mathrm{boot}}$	0.198	0.239	0.117	0.14
	$\mathrm{P}_{\mathrm{boot}}$	0.00579	0.000945	0.0815	0.921
	% $\mathrm{P}_{\mathrm{bonf.sign.}}$	25.7	50.3	5.1	0.1
	% $\mathrm{P}_{\mathrm{nom.sign.}}$	81.9	93.8	40.9	4.2

metabolite	statistic	*APOE* ε4+ males	*APOE* ε4+ females	*APOE* ε4- males	*APOE* ε4- females
	β_0	0.42	0.761	0.194	-0.00631
	P_0	0.0315	0.000865	0.106	0.965
	bias (β_0)	0.0212	0.0315	-0.0058	0.000854
PC ae C42:4	β_{boot}	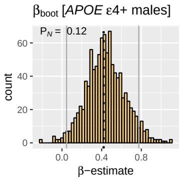	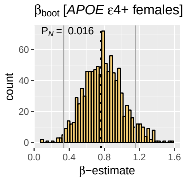	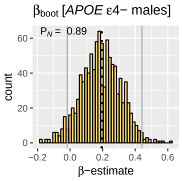	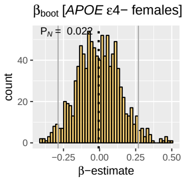
	$\mu(\beta)_{\mathrm{boot}}$	0.441	0.793	0.189	-0.00546
	$\mu(\mathrm{SE})_{\mathrm{boot}}$	0.202	0.241	0.123	0.149
	$\mathrm{P}_{\mathrm{boot}}$	0.0293	0.000977	0.124	0.971
	% $\mathrm{P}_{\mathrm{bonf.sign.}}$	9	50.9	2.2	0
	% $\mathrm{P}_{\mathrm{nom.sign.}}$	58.9	94.7	34.4	4.4

CSF p-tau

metabolite	statistic	*APOE* ε4+ males	*APOE* ε4+ females	*APOE* ε4- males	*APOE* ε4- females
	β_0	-0.0642	0.264	0.0635	0.0746
	P_0	0.324	0.000121	0.278	0.23
	bias (β_0)	0.00401	-0.00236	-0.00472	0.00132
C10	β_{boot}	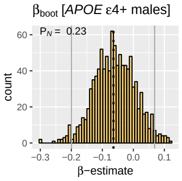	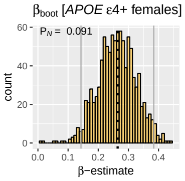	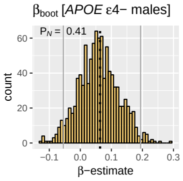	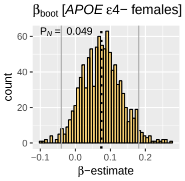
	$\mu(\beta)_{\mathrm{boot}}$	-0.0602	0.262	0.0588	0.0759
	$\mu(\mathrm{SE})_{\mathrm{boot}}$	0.0656	0.0682	0.0585	0.0623
	$\mathrm{P}_{\mathrm{boot}}$	0.359	0.000121	0.315	0.223
	% $\mathrm{P}_{\mathrm{bonf.sign.}}$	1.2	69.7	2	1.1
	% $\mathrm{P}_{\mathrm{nom.sign.}}$	14.7	97.5	19.7	19.3

FDG-PET

metabolite	statistic	*APOE* ε4+ males	*APOE* ε4+ females	*APOE* ε4- males	*APOE* ε4- females
Proline	β_0	0.0464	-0.272	0.082	0.0132
	P_0	0.476	8.22E-05	0.185	0.829
	bias (β_0)	-0.00307	-0.00254	0.000889	-0.00121
	β_{boot}	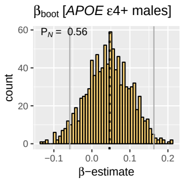	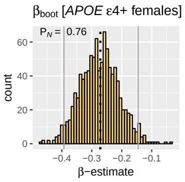	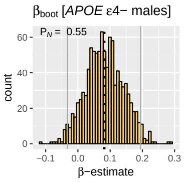	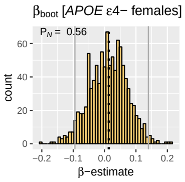
	$\mu(\beta)_{boot}$	0.0433	-0.274	0.0829	0.012
	$\mu(SE)_{boot}$	0.0652	0.0683	0.0618	0.0615
	P_{boot}	0.506	6.04E-05	0.179	0.845
	% $P_{bonf.sign.}$	0	74.1	1.6	0.1
	% $P_{nom.sign.}$	7.7	98.1	25.7	4.3
