# Supplementary material for: Sex and APOE ε4 genotype modify the Alzheimer’s disease serum metabolome
Source: Nat Commun. 2020 Mar 2;11:1148. doi: 10.1038/s41467-020-14959-w (PMC7052223; doi:10.1038/s41467-020-14959-w)
Supplement: Supplementary file 10 — Reporting Summary [file 41467_2020_14959_MOESM10_ESM.pdf]

nature research

Corresponding author(s): Gabi Kastenmüller
Rima Kaddurah-Daouk

Last updated by author(s): Oct 24, 2019

Reporting Summary

Nature Research wishes to improve the reproducibility of the work that we publish. This form provides structure for consistency and transparency in reporting. For further information on Nature Research policies, see Authors & Referees and the Editorial Policy Checklist .

Statistics

For all statistical analyses, confirm that the following items are present in the figure legend, table legend, main text, or Methods section.

n/a	Confirmed	
☐	☒	The exact sample size (*n*) for each experimental group/condition, given as a discrete number and unit of measurement
☐	☒	A statement on whether measurements were taken from distinct samples or whether the same sample was measured repeatedly
☐	☒	The statistical test(s) used AND whether they are one- or two-sided<br>*Only common tests should be described solely by name; describe more complex techniques in the Methods section.*
☐	☒	A description of all covariates tested
☐	☒	A description of any assumptions or corrections, such as tests of normality and adjustment for multiple comparisons
☐	☒	A full description of the statistical parameters including central tendency (e.g. means) or other basic estimates (e.g. regression coefficient) AND variation (e.g. standard deviation) or associated estimates of uncertainty (e.g. confidence intervals)
☐	☒	For null hypothesis testing, the test statistic (e.g. *F*, *t*, *r*) with confidence intervals, effect sizes, degrees of freedom and *P* value noted<br>*Give P values as exact values whenever suitable.*
☒	☐	For Bayesian analysis, information on the choice of priors and Markov chain Monte Carlo settings
☒	☐	For hierarchical and complex designs, identification of the appropriate level for tests and full reporting of outcomes
☐	☒	Estimates of effect sizes (e.g. Cohen's *d*, Pearson's *r*), indicating how they were calculated

Our web collection on statistics for biologists contains articles on many of the points above.

Software and code

Policy information about availability of computer code

Data collection	*Provide a description of all commercial, open source and custom code used to collect the data in this study, specifying the version used OR state that no software was used.*
Data analysis	R version 3.6.1

For manuscripts utilizing custom algorithms or software that are central to the research but not yet described in published literature, software must be made available to editors/reviewers. We strongly encourage code deposition in a community repository (e.g. GitHub). See the Nature Research guidelines for submitting code & software for further information.

Data

Policy information about availability of data

All manuscripts must include a data availability statement. This statement should provide the following information, where applicable:

- Accession codes, unique identifiers, or web links for publicly available datasets
- A list of figures that have associated raw data
- A description of any restrictions on data availability

Data availability
Metabolomics datasets from the AbsoluteIDQ-p180 metabolomics kit used in the current analyses for the ADNI-1, ADNI-GO/-2, and ROS/MAP cohorts are available via the Accelerating Medicines Partnership-Alzheimer's Disease (AMP-AD) Knowledge Portal and can be accessed at http://dx.doi.org/10.7303/syn5592519 (ADNI-1), http://dx.doi.org/10.7303/syn9705278 (ADNI-GO/-2), and http://dx.doi.org/10.7303/syn10235592 (ROS/MAP). The full complement of clinical and demographic data for the ADNI cohorts are hosted on the LONI data sharing platform and can be requested at http://adni.loni.usc.edu/data-samples/access-data/. The full complement of clinical and demographic data for the ROS/MAP cohorts are available via the Rush AD Center Resource Sharing Hub and can be requested at https://www.radc.rush.edu. AIBL data is available upon request at https://aibl.csiro.au. Source data are provided as Source Data File.

Field-specific reporting

Please select the one below that is the best fit for your research. If you are not sure, read the appropriate sections before making your selection.

- [x] Life sciences
- [ ] Behavioural & social sciences
- [ ] Ecological, evolutionary & environmental sciences

For a reference copy of the document with all sections, see nature.com/documents/nr-reporting-summary-flat.pdf

Life sciences study design

All studies must disclose on these points even when the disclosure is negative.

Sample size

We included all cohort participants from ADNI, ROS/MAP, and AIBL that had data on the investigated variables (metabolomics, biomarker profiles, forced-in and selectable covariates) available. More information is provided in the methods section and supplementary information of our manuscript.

Data exclusions

We included all cohort participants from ADNI, ROS/MAP, and AIBL that had data on the investigated variables (metabolomics, biomarker profiles, forced-in and selectable covariates) available. More information is provided in the methods section and supplementary information of our manuscript.

Replication

Internal evaluation of result robustness was assessed using bootstrap analysis on ADNI findings, which provided support for all reported results (there were three instances of borderline significance that nonetheless achieved the expected power). External replication was performed in samples from study participants of ROS/MAP and AIBL. Those validated all 3 overall significant associations reported and 1 significant heterogeneity estimate. Other findings were not significant in the replication sets, but showed trends supportive of our findings. Reasons for failed replication include: significant lack of power based on low sample sizes in the replication cohorts, investigation of proxies for both the biomarkers and metabolites investigated in ADNI (exactly the same measurements were not available in any replication cohort identifiable by us), and unbalanced sample sizes in stratified analyses. Replication of associations for FDG-PET imaging markers were impossible as we could not identify a study having both FDG-PET measures and metabolomics data available. More details and a thorough discussion of these aspects are provided in our manuscript and the supplement.

Randomization

Inclusion/exclusion criteria for ADNI, AIBL, and ROS/MAP are available in online resources. ADNI participants included were very well balanced with respect to age, gender, APOE e4 status, and other important variables. Information on diagnostic groups was not included in our study, we only investigated biomarker profiles. Significant covariates were investigated both by statistical means (backwards selection) and by prior knowledge (risk estimates from previous studies), and included in statistical models if identified as significant.

For metabolomics measurements (and other measurements such as biomarker profiles), order of analysis/assignment to batches/selection of blinded replicates/... was randomized on blinded sample identifiers.

Blinding

For metabolomics measurements (and other measurements such as biomarker profiles), order of analysis/assignment to batches/selection of blinded replicates/... was randomized on blinded sample identifiers. In the analysis, unblinding of samples was performed after primary data quality control and only influenced data selection/sample exclusion based on one variable, namely fasting status.

Reporting for specific materials, systems and methods

We require information from authors about some types of materials, experimental systems and methods used in many studies. Here, indicate whether each material, system or method listed is relevant to your study. If you are not sure if a list item applies to your research, read the appropriate section before selecting a response.

Materials & experimental systems

n/a	Involved in the study	
x		Antibodies
x		Eukaryotic cell lines
x		Palaeontology
x		Animals and other organisms
x		Human research participants
x		Clinical data

Methods

n/a	Involved in the study	
x		ChIP-seq
x		Flow cytometry
x		MRI-based neuroimaging
