# Supplementary material for: Sex and APOE ε4 genotype modify the Alzheimer’s disease serum metabolome
Source: Nat Commun. 2020 Mar 2;11:1148. doi: 10.1038/s41467-020-14959-w (PMC7052223; doi:10.1038/s41467-020-14959-w)
Supplement: Supplementary file 11 — Source Data [file 41467_2020_14959_MOESM11_ESM.zip › NatComms_source_data_file.html]

Source Data


# Source Data

#### Matthias Arnold, PhD

#### Nov 05, 2019

# The Alzheimer’s Disease Metabolome: Effects of Sex and *APOE* \(\varepsilon4\) genotype

## Figure 1

## Figure 2
